# Spatiotemporal Variability of the Pepper Mild Mottle Virus Biomarker in Wastewater

**DOI:** 10.1021/acsestwater.4c00866

**Published:** 2024-12-16

**Authors:** AnnaElaine L. Rosengart, Amanda L. Bidwell, Marlene K. Wolfe, Alexandria B. Boehm, F. William Townes

**Affiliations:** † Department of Statistics & Data Science, Dietrich College of Humanities and Social Sciences, Carnegie Mellon University, Pittsburgh, Pennsylvania 15213, United States; ‡ Department of Civil & Environmental Engineering, School of Engineering and Doerr School of Sustainability, Stanford University, Stanford, California 94305, United States; § Gangarosa Department of Environmental Health, Rollins School of Public Health, Emory University, Atlanta, Georgia 30322, United States

**Keywords:** wastewater, normalization, biomarker, variance, spatiotemporal, epidemiology

## Abstract

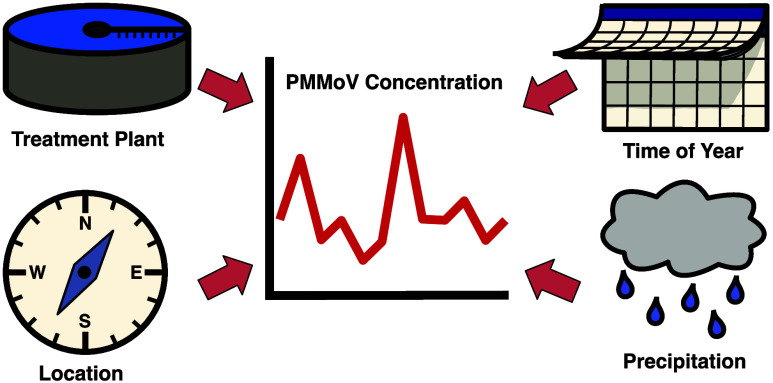

Since the start of the coronavirus-19 pandemic, the use of wastewater-based epidemiology (WBE) for disease surveillance has increased throughout the world. Because wastewater measurements are affected by external factors, processing WBE data typically includes a normalization step in order to adjust wastewater measurements (e.g., viral ribonucleic acid (RNA) concentrations) to account for variation due to dynamic population changes, sewer travel effects, or laboratory methods. Pepper mild mottle virus (PMMoV), a plant RNA virus abundant in human feces and wastewater, has been used as a fecal contamination indicator and has been used to normalize wastewater measurements extensively. However, there has been little work to characterize the spatiotemporal variability of PMMoV in wastewater, which may influence the effectiveness of PMMoV for adjusting or normalizing WBE measurements. Here, we investigate its variability across space and time using data collected over a two-year period from sewage treatment plants across the United States. We find that most variation in PMMoV measurements can be attributed to longitude and latitude followed by site-specific variables. Further research into cross-geographical and -temporal comparability of PMMoV-normalized pathogen concentrations would strengthen the utility of PMMoV in WBE.

## Abstract 2

PMMoV is a widely used proxy for human fecal content used to normalize pathogen measurements in WBE. We investigate its spatiotemporal variability using nationwide data from mid-2021 through mid-2023 from United States sewage treatment plants.

## 1 Introduction

Wastewater-based epidemiology (WBE) is now a well established method of surveilling community health over a large area.^1,2^ For certain viral illnesses, such as COVID-19 and Influenza, infected individuals shed pathogenic genetic material (analyte) in their feces, urine, and sputum, which then enters a sewer system.^3^ Quantitative and droplet digital (reverse transcription-) polymerase chain reaction (PCR) are cost- and time-effective methods for absolute quantification of viral genetic material in a wastewater sample.^4^ Under the assumption that changes in the number of viral gene copies determined by PCR are reflective of changes in the number of infections in an area, these measurements from wastewater can be used to infer trends in community disease burden.^5−8^

However, wastewater measurements are subject to other sources of variation: in-human, in-sewer, and in-lab effects.^9^ In-human effects occur prior to an analyte’s entry into the sewer system and may be caused by changes in population (e.g., for a sporting event) or fecal shedding rates for different strains of a virus. In-sewer effects occur during sewer travel and include dilution due to groundwater infiltration, degradation of genetic material caused by fluctuating temperature or pH, or the adsorption rate of genetic material to solid waste. In-lab effects occur during sampling and analysis and can be caused by differences in sampling (e.g., solid vs liquid samples) and laboratory protocols (e.g., freezing and transport of samples, instrument bias, nucleic acid extraction efficiency). These sources of variability are due to the environment, not necessarily disease dynamics, and may introduce noise that reduces the correlation between wastewater measurements and disease incidence (or prevalence, though evidence suggests shedding, at least in the case of SARS-CoV-2, is greatest in the early stages of infection, making incidence more relevant).^9,10^

Normalizing concentrations attempts to account for these effects to improve comparisons across space and time. Metadata, such as the catchment population size or the flow rate of water entering a wastewater treatment facility (site), can be used to adjust for in-human effects and certain in-sewer effects like groundwater infiltration. For example, flow normalized concentration can be calculated as
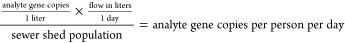
Days with greater precipitation may yield smaller raw concentrations due to dilution, not a decrease in cases. Multiplying by flow rate can adjust for this sewer effect and thereby improve the ability of the wastewater measurements to represent disease incidence. However, metadata normalization cannot adjust for in-lab effects because the information it uses is not intrinsic to a sample.

Biomarkers used for normalization are substances, such as metabolites, chemical compounds, and biological agents, present in a sample and are thought to enter the sewer system and be affected by wastewater and laboratory methods in ways similar to the analyte of interest. Biomarker normalization is performed by calculating the ratio of an analyte’s concentration to that of the chosen biomarker, for example:
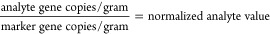
Though the result is a unitless measure, normalization by biomarkers has the potential to correct for all three types of environmental variation.^9,11−13^

A popular biomarker for wastewater normalization is pepper mild mottle virus (PMMoV), a virus infecting the genus *Capsicum*, including bell and spicy peppers, that appears in human fecal matter after the consumption of infected plants. PMMoV has previously been used as an indicator for the presence or absence of fecal contamination in ocean water, river water, and treated wastewater^14−17^ and exhibits properties that give it promise as a normalizing agent. It is one of the most abundant RNA viruses found in human feces, making it easily quantifiable.^18^ Though it is nonenveloped, PMMoV is a single-stranded RNA virus like SARS-CoV-2 and many other viruses,^19,20^ and therefore it may experience effects through sewer system travel similar to that of these pathogens of interest.^11,17,19^ However, its utility in practice has been relatively inconclusive. Several studies have shown that pathogen concentrations normalized with PMMoV have improved correlations with disease incidence compared to raw concentrations.^3,21^ Wolfe et al.^7^ showed through a mass-balance model that concentrations of SARS-CoV-2 RNA scaled by PMMoV RNA were directly proportional to COVID-19 incidence rates. In contrast, others have reported only mild increases or decreases in correlations.^8,13,22,23^

PMMoV concentrations may differ throughout the year and across different regions, which may confound its use as a WBE normalizer and explain some of the mixed results in the literature. Its dietary origin may be one source of variability as fluctuations in regional or temporal popularity and availability of foods, such as salsa, hot sauce, and certain spices, may contribute to the wide range of results characterizing its variation.^14,18^ PMMoV concentrations have been found to be relatively stable in comparison to other human fecal indicators^23^ but also to vary greatly both across regions and over time.^16,24,25^ One study reported PMMoV concentrations to exhibit little evidence of seasonal trend^11^ while another showed mild seasonality.^26^ Even within a single individual, PMMoV shedding rates can vary greatly over time.^10^

It is this uncertainty in the characteristics of PMMoV concentration over space and time that motivate this work. We use data from the ongoing wastewater sampling project, WastewaterSCAN,^27^ and fit four models that incorporate geographical, temporal, and site-specific information in order to investigate three questions of interest: (i) how does PMMoV concentration vary across locations; (ii) how does it vary over time; and (iii) what proportion of its variation can be accounted for solely by these spatiotemporal factors. We are able to quantify the variance explainable, visualize the trends in PMMoV concentration based upon variables of interest, and suggest potential contributors to the variation.

## 2 Methods

### 2.1 Data

The data comprise PMMoV concentrations taken from wastewater samples across the United States and collected as part of the WastewaterSCAN project.^27^ Viral RNA copies were quantified from wastewater solids using reverse transcription droplet digital PCR. These extraction and quantification procedures have previously been described in detail.^28−30^ Additionally, the full methods can be found in two data descriptors.^31,32^

Sites with at least 30 samples collected from May 29th, 2021, through August 18th, 2023, were included in the analysis for a total of 25,383 observations from 160 sites across 31 states. Each site was classified as having a separated or combined sewer system depending upon whether the system accepted sanitary and runoff water together. System classifications were obtained through communications with each site upon admission to the WastewaterSCAN project, though sites associated with an outfall location listed in the EPA’s National Combined Sewer Overflow Inventory were classified as having combined systems.^33^

Precipitation data were collected from the Global Historical Climatology Network daily database from the National Centers for Environmental Information of the National Oceanic and Atmospheric Administration.^34^ The average precipitation by day was calculated for each site by taking the mean of daily measurements over all stations located in all counties served by the plant. Latitude and longitude data were taken as the centroid of the ZIP code associated with each site. Most sites had over 100 samples, and most samples were taken on days with 0 in. of precipitation (Tables 1 and S14 for summary statistics by site).

**Table 1:** Summary Statistics for the Number of Observations Per Site, PMMoV Concentration Across All Sites, and the Average Daily Precipitation Across All Sites^a^

	number of observations	PMMoV (log_10_ gc/g)	average precipitation (in)
min	30	5.92	0.00
max	812	11.31	4.34
med	112	8.78	0.00
mean	158.64	8.77	0.10

a (gc = gene copies; g = grams; in = inches)

### 2.2 Modeling

We fit four different models that describe the conditional distribution of log_10_ PMMoV concentration using a linear combination of spatiotemporal factors. We use the following notation where *i* indexes the sites: *lat*_*i*_ = latitude in degrees; *lng*_*i*_ = longitude in degrees; *sewer*_*i*_ = sewer system type (1 for combined and 0 for separated); *prcp*_*i*,*t*_ = average precipitation *t* days after May 28th, 2021, in inches; *log_10_PMMoV*_*i*,*t*_ is the log_10_ PMMoV concentration *t* days after May 28th, 2021, in gene copies per gram dry weight; *siteID*_*i*_ is an indicator value for site *i* (a dummy variable encoding the site from which a sample was obtained). A level of α = 0.05 was used for determining statistical significance.

#### 2.2.1 Simple Median Model

The simple median model is a quantile regression model for the median log_10_ PMMoV concentration
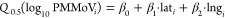
1where *Q*_τ_(·) denotes the τ-th quantile of the distribution of the random variable of interest conditional on some set of covariates, which are taken here to be latitude and longitude. This model was used for investigating the variation in PMMoV concentration solely on the basis of the geographic origin of a sample.

#### 2.2.2 Detailed Median Model

The detailed median model expands upon the simple median model by including average daily precipitation, sewer system type, and their interaction. Fourier basis functions with week- and year-long periods are also included, motivated by the potential seasonality in pepper consumption as well as evidence of a weekly pattern in the autocorrelation of the data in exploratory analysis (Figure S2a).

Each pair of basis functions is defined as
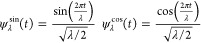
2where λ corresponds to the period of the bases in days. For weekly trends we set λ = 7, and for annual trends we set λ = 365.25. This model assumes the form
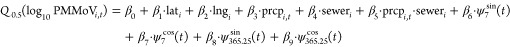
3To guard against model misspecification, we used the cluster-robust bootstrap for inference, which does not require assumptions on the distribution of the errors.^35^

#### 2.2.3 Variance Decomposition Model

We fit a variance decomposition model in order to attribute portions of the variability to spatial, temporal, and site-specific sources by partitioning the model’s coefficient of determination, *R*^2^. This model assumes the conditional mean, rather than the median, of log_10_ PMMoV can be described by the chosen covariates, and that the errors have finite variance (but not necessarily that they are normally distributed)
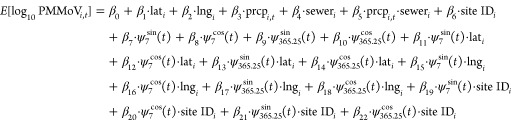
4We obtained the partition by successively adding groups of covariates until reaching the final model described in eq 4, recording the *R*^2^ at each step. These values represent the percent variation explained by the newly added covariates that remained unexplained by the covariates in the previous, smaller model fit. The order of covariate addition was: (i) latitude and longitude; (ii) precipitation, sewer system type, and their interaction; (iii) site indicator; (iv) the weekly and yearly time components; (v) interaction terms between latitude, longitude, and the temporal components; (vi) interaction terms between the site indicator and the temporal components.

#### 2.2.4 Bayesian Median Model

For each site separately, we fit a Bayesian quantile regression model using precipitation and both weekly and yearly time components. We assume for each site independently:
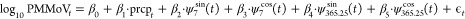
5where ϵ_*t*_ are independent and identically distributed Laplace random variables with location of 0 and scale of σ.^36^

Inference was done with Hamiltonian Monte Carlo using the No–U-Turn Sampler variant.^37,38^ The intercept, β_0_, was given a uniform prior over the real line, while every other β_*i*_ was given a Gaussian prior with 0 mean and standard deviation σ_*i*_. We used a Student’s *t* prior with 5 degrees of freedom, location of 0, and scale of 1 for σ as well as each σ_*i*_. Further details on the fitting regime can be found in the supplementary code scripts.

We used these models to visually describe the temporal variation in PMMoV concentration by comparing the model predictions with the observed values. Fitting to each site separately afforded us the ability to investigate differences in the effects of our chosen covariates for different sites.

### 2.3 Data Analysis

Data analyses were performed in R version 4.1.0 (Camp Pontanezen)^39^ using RStudio version 2024.04.0 + 764.^40^ The simple and detailed median models were fit using the*quantreg* package,^41^ and the cluster-robust wild bootstrap with site membership grouping was used for uncertainty quantification.^35^ The variance decomposition model was fit with the *stats* package,^39^ and the Bayesian median models were fit with the *rstan* package.^42^ A detailed list of packages used in the analyses can be found in Section S1.

## 3 Results and Discussion

### 3.1 Geographic Variation in PMMoV Concentration

**Figure 1:**
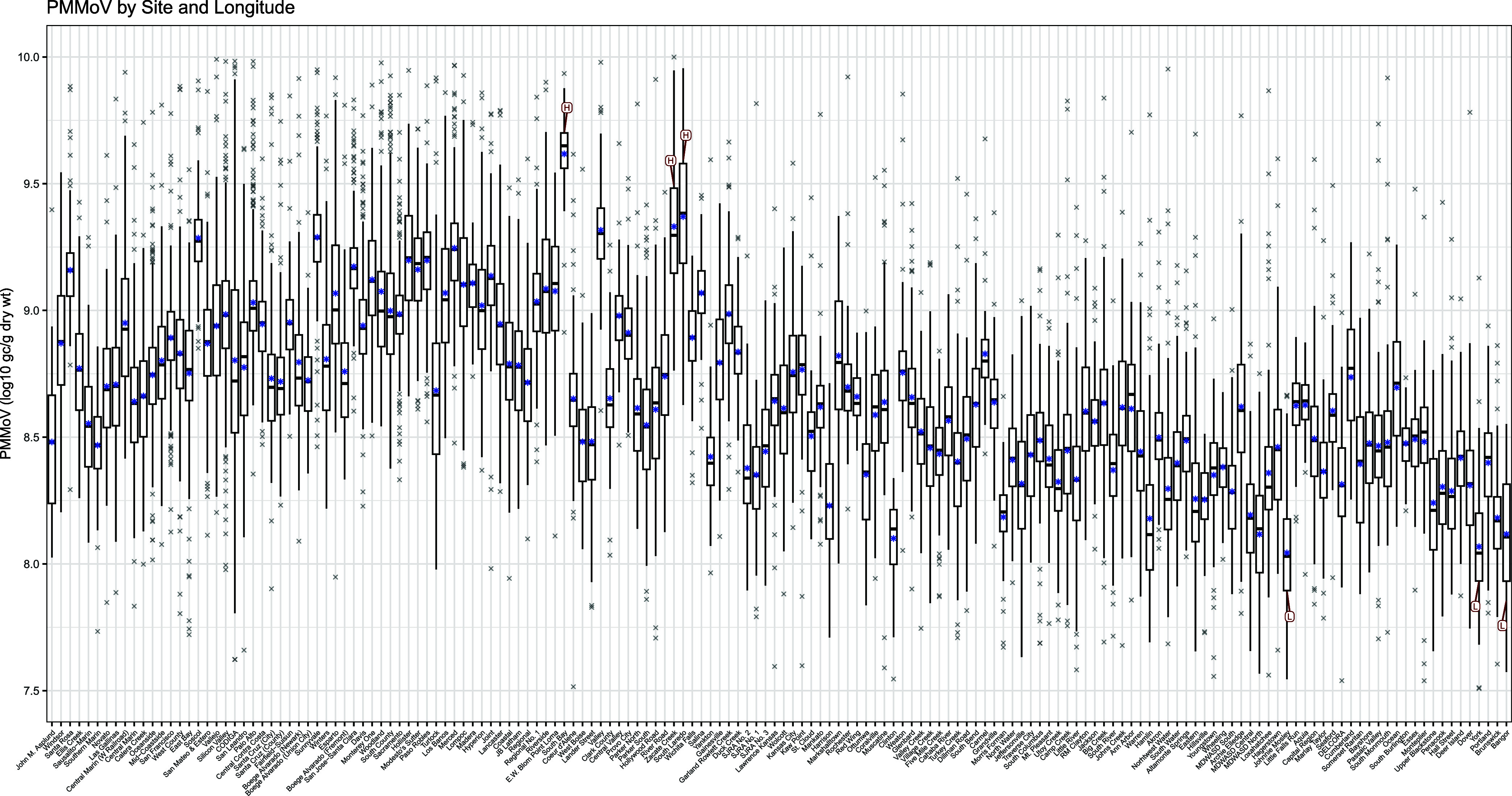
PMMoV concentration varies within and between sites. Sites ordered by longitude (left to right = west to east). Top and bottom of each box demarcate the interquartile range (75th and 25th percentiles, respectively) for concentrations taken from each site. Black horizontal lines and blue stars mark site median and mean concentrations, respectively. Observations falling outside the vertical whiskers have values exceeding 1.5 times the interquartile range and are marked with a gray *x*. The three sites with the highest median concentrations are marked with a red H, and the three sites with the lowest medians are marked with a red L. Vertical axis limited to 7.5 to 10.0 log_10_ gc/g dry wt for visibility. See Table S11 for site name abbreviations. (gene copies = gc; gram = g; weight = wt).

PMMoV concentration varies widely both within and across sites. The majority of sites have concentrations spanning at least 1 order of magnitude, and even the smallest interquartile range (South Burlington) ranges from 8.41 to 8.54 log_10_ gene copies per gram dry weight. Median concentration tends to decrease with increasing site longitude (i.e., moving east), and sites located in the west experience concentrations that are generally higher, on average, than those experienced by sites in the midwest and east (Figure 1).

**Figure 2:**
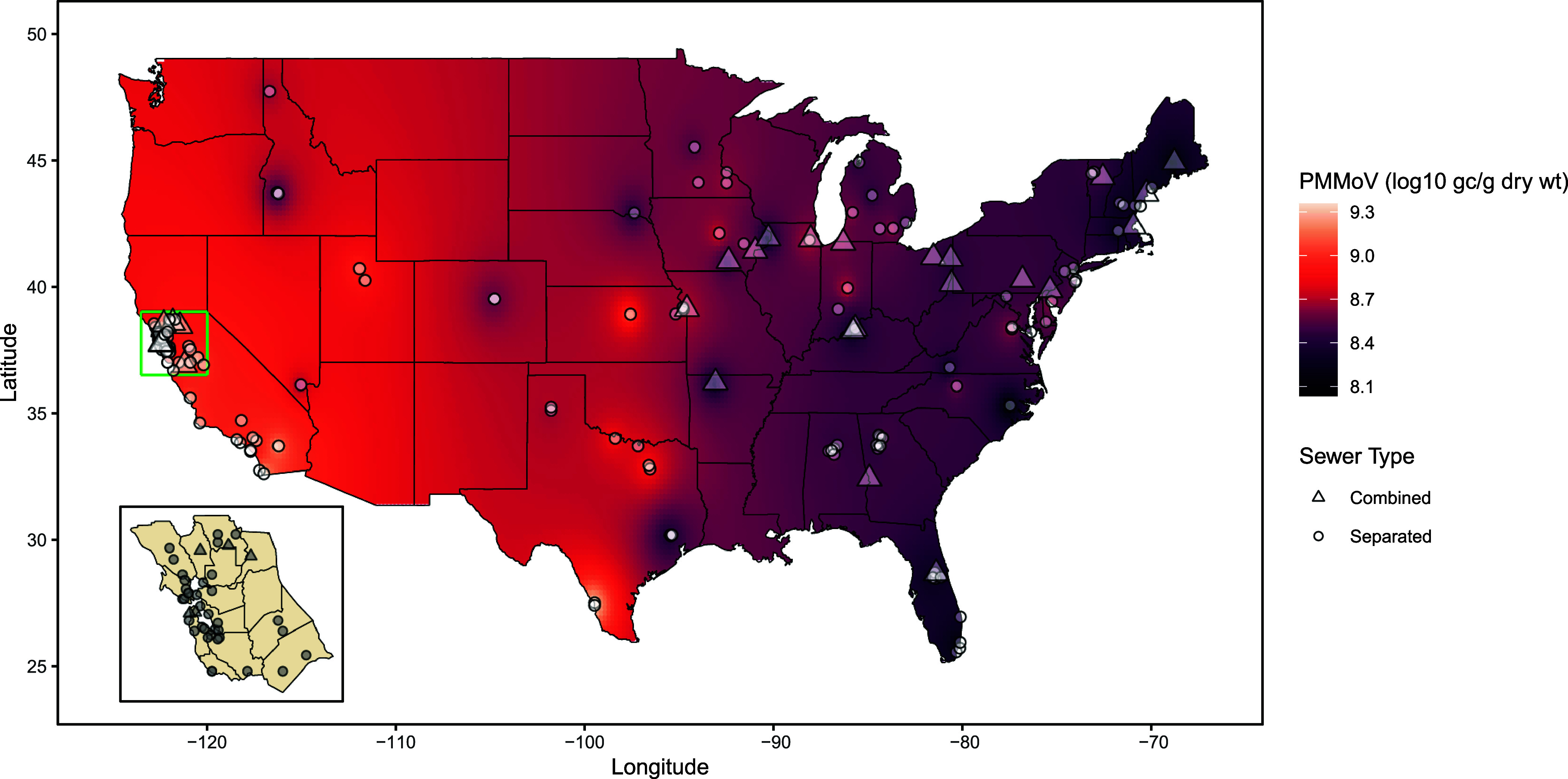
Median PMMoV concentration tends to be greater in western regions compared to northeastern areas in the contiguous United States. Sampled site locations as points shaped by sewer type are superimposed on a color gradient (main) illustrating the concentration as interpolated with inverse distance weighting with a power of 1.601 (chosen by cross-validation). Sites in the Bay Area and nearby counties in higher resolution (inset) on a neutral background. Alaska omitted for visibility. (gene copies = gc; gram = g; weight = wt).

The color shift when moving across the map reiterates the association between longitude and PMMoV concentration (Figure 2). This result is confirmed by the statistical significance in the coefficient on longitude (β = −1.29 × 10^–2^; *p* < 1.00 × 10^–15^) of the simple median model. The negative coefficients on both latitude and longitude (Table S1) indicate that predicted log_10_ PMMoV concentration decreases with more northern and more eastern sampling locations, though the north–south relationship is less pronounced due to the lack of statistical significance on latitude. Even when including additional covariates, as in the detailed median model, the sign and statistical significance of longitude remain (β = −1.28 × 10^–2^; *p* < 1.00 × 10^–15^). The coefficient for latitude is positive, though it remains small in magnitude and not statistically significant.

The three sites with the lowest median concentrations are Johnnie Mosley Regional Water Reclamation Facility in Kinston, NC; York Sewer District in York Beach, ME; and the City of Bangor Wastewater Treatment Plant in Bangor, ME (Figure 1, Ls). All three of these sites are located on the eastern coast of the United States. The three sites with the highest median concentrations are South Bay International Wastewater Treatment Plant in San Diego, CA; South Laredo Wastewater Treatment Plant in Laredo, TX; and Zacate Creek Wastewater Treatment Plant in Laredo, TX (Figure 1, Hs). South Laredo and Zacate Creek are located along the Rio Grande River on the Mexico border, and South Bay treats sewage from Tijuana, Mexico.^43^

We hypothesize that this variation may be driven by geographical differences in diet. However, there is also the possibility that this geographic trend is due to viral genetic material loss during transportation as all of the samples were processed at the same laboratory in the San Francisco Bay Area. The length of time between sampling and processing may be longer for sites located farther from the laboratory than those in closer regions, and effects due to packaging and travel may be greater for samples from those more distant sites. Though exact sample collection and processing times were not available, samples were processed within 48 h of collection, and most were processed within 24 h.^32^ We fit a modified version of the detailed median model in which the latitude and longitude terms were replaced with the distance of each site to the processing laboratory and the distance of each site to El Paso, Texas (see Section S2). The former was meant to capture any effect due to transportation of the sample to the laboratory, while the latter served as a loose proxy for how southwestern a site is.

We find that the variable for the distance to the laboratory (β = −1.26 × 10^–7^; *p* < 1.00 × 10^–15^) is statistically significant (Table S3), which suggests that it may have an effect on PMMoV concentration. However, the variable for the distance to El Paso (β = −8.78 × 10^–8^; *p* < 1.00 × 10^–15^) is also statistically significant, and the sites with the highest median PMMoV concentrations are not those closest to the processing laboratory (Figure 1). These results suggest that, though the distance to the laboratory may affect PMMoV concentration, there is still some other geographic trend.

Previous studies have found evidence that PMMoV degradation rates are lower than those of other fecal indicators in certain cases, potentially due to the structure of the virus.^15,44^ Zhang et al.^45^ report stability of PMMoV RNA concentrations even when kept at 37C for up to 50 days. Because samples were processed within 48 h and kept at 4C between collection and processing,^31,32^ we speculate that the effect of transportation is minimal, and a geographic or dietary trend may be more likely. In addition, because the processing laboratory is located in the western United States, the two added variables are highly correlated (Pearson correlation of 0.7). It could be the case that the distance to the laboratory is also a proxy for how southwestern a site is. Disentangling these two effects and whether diet plays a role would require additional data on pepper consumption, and future research may include pepper product sales data and times between sampling and processing in regression analyses.

The stability of PMMoV in wastewater implies that PMMoV normalization may not be able to adjust for effects occurring in transit. If PMMoV concentrations are stable while the analyte of interest degrades in the time between collection and processing, normalized values may be lower due to this decay rather than a corresponding decrease in disease burden. However, Zhang et al.^45^ also show that human respiratory viruses, like SARS-CoV-2, exhibit minimal RNA decay in wastewater-settled solids even after 50 days when kept at 4C. Therefore, storage and transport conditions may mitigate this potential issue.

### 3.2 Variance Partition

**Figure 3:**
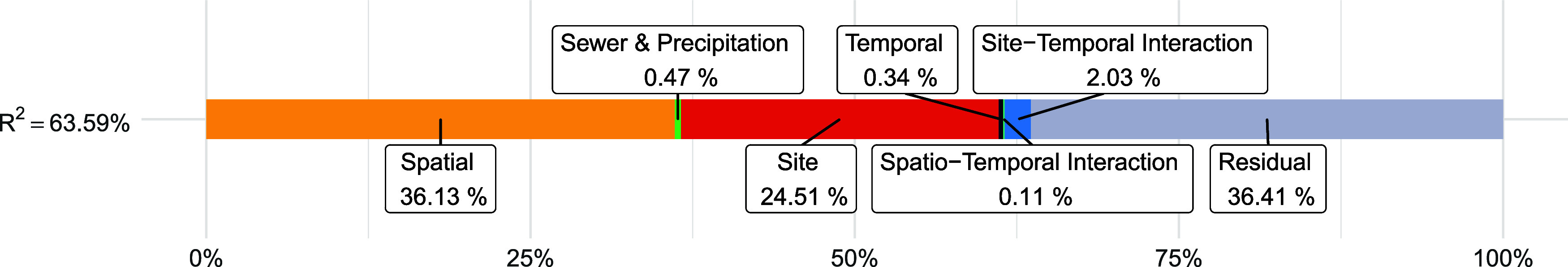
Location and site membership account for the majority of the variation in PMMoV concentration. The spatial components (latitude and longitude) account for the greatest portion at about 36%. Site membership accounts for over 24% of the variation that remains unexplained by the spatial, sewer, and precipitation variables. The temporal components, sewer system type, and precipitation altogether account for less than 1% of the variation. Site- and location-specific temporal features explain only 2.14% of the remaining variation.

The majority of the variation in PMMoV concentration can be accounted for by spatial variables and an indicator variable encoding the site from which a sample was collected (Figure 3). Moreover, geographic location (latitude and longitude) of a sample as well as site membership, both of which are sources of between-site variability, are the two greatest sources of variation in PMMoV concentration. These two groups of covariates explain over 36 and 24% of the variance, respectively, in the variance decomposition model, which agrees with the visual and statistical results from the simple median and detailed median models. Precipitation, sewer type, and temporal variables account for some of the variability as well, although the portion is quite small in comparison to geography and site membership. The interaction of the temporal components with the spatial and site indicator terms accounts for only around 2% of the variance.

The remaining variance (Figure 3, rightmost bar) is within-site variation that is unaccounted for by our chosen covariates. This may be due to microscale variation or noise; we are unable to investigate further due to data limitations.

Recent work has found evidence that factors, such as alkalinity, biochemical oxygen demand, and flow rate, can affect PMMoV concentrations through dilution, degradation, and adsorption of genetic material to solids during sewer travel. Even for sites serving the same city, physicochemical parameters can have statistically significant differences in levels.^46^ Thus, the large portion of the variation attributable to site membership in Figure 3 may be due to the physical and chemical attributes of the sewer system sampled.

### 3.3 Temporal Variation

**Figure 4:**
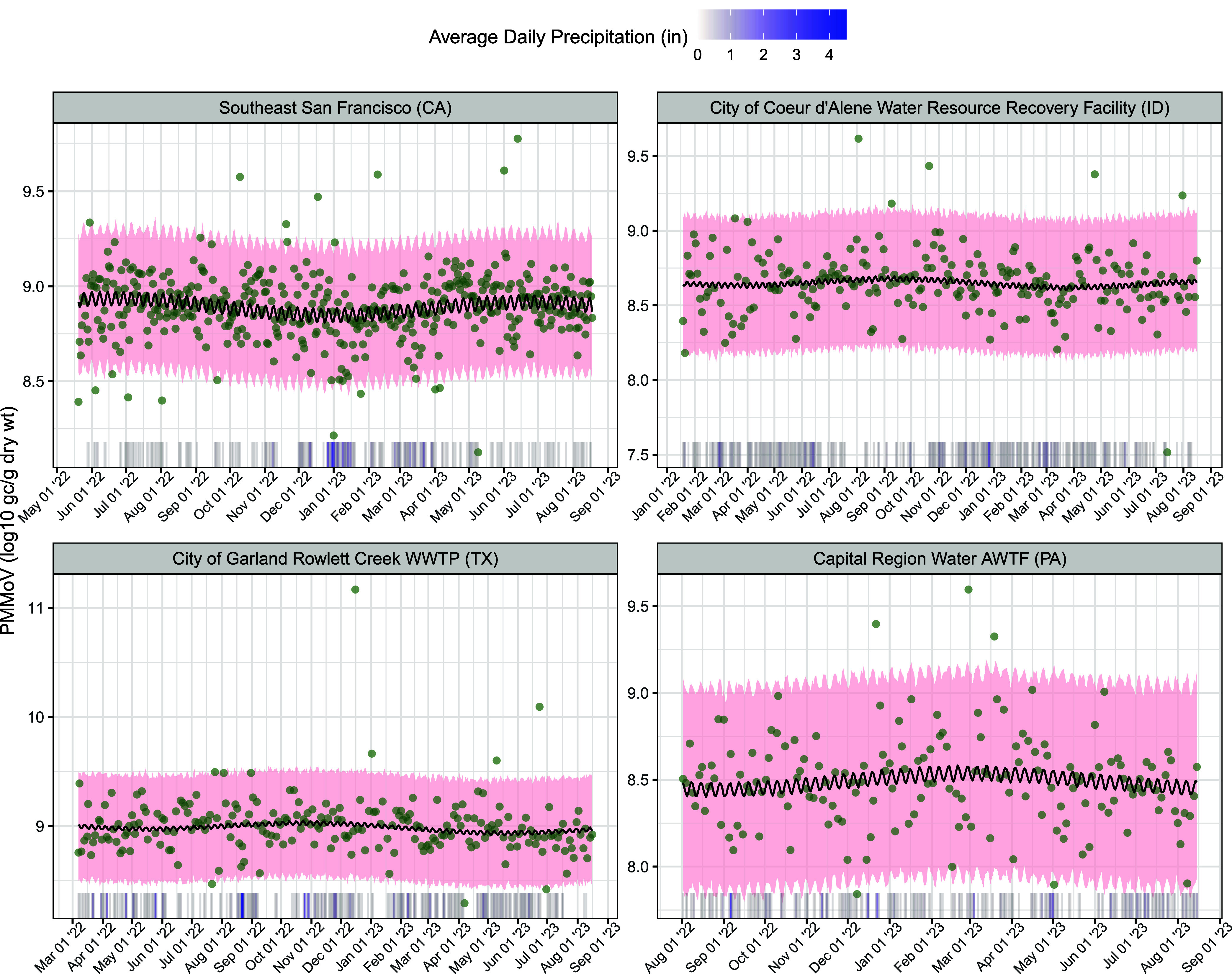
PMMoV concentration varies over time, both on a weekly and yearly scale, and in different ways for different sites. Black line is the median predicted PMMoV of 4000 posterior samples. Lower and upper limits of pink ribbon are 2.5 and 97.5% quantiles, respectively, from the samples. Green points show observed concentrations for available dates. Rug shows average daily precipitation. Sites were chosen to illustrate results for different US regions. (gene copies = gc; gram = g; weight = wt; inches = in).

Patterns in day-to-day and seasonal changes in PMMoV concentration vary by site. Weekly variation is reflected in the tight oscillation of the median predicted concentrations (Figure 4); however, the proportion of sites with statistically significant weekly terms was quite small (12/160 for the coefficient on ψ_7_^sin^ and 23/160 for the coefficient on ψ_7_^cos^; Tables S7 and S8). Seasonality is illustrated in the wider sinusoidal pattern (Figure 4). For example, Southeast San Francisco in California sees generally higher concentrations in the summer, while greater concentrations occur in late winter for Capital Region Water in Pennsylvania. Moreover, a larger proportion of yearly terms were statistically significant (51/160 for the coefficient on ψ_365.25_^sin^ and 42/160 for the coefficient on ψ_365.25_^cos^; Tables S9 and S10).

This temporal variability is in agreement with some prior research into the variation of PMMoV over time^47^ but contrasts with others that did not report evidence of seasonal patterns.^15,19^ Additional long-term data collection of PMMoV concentrations would be beneficial for defining this time-based variation with more certainty.

### 3.4 Effect of Precipitation

**Figure 5:**
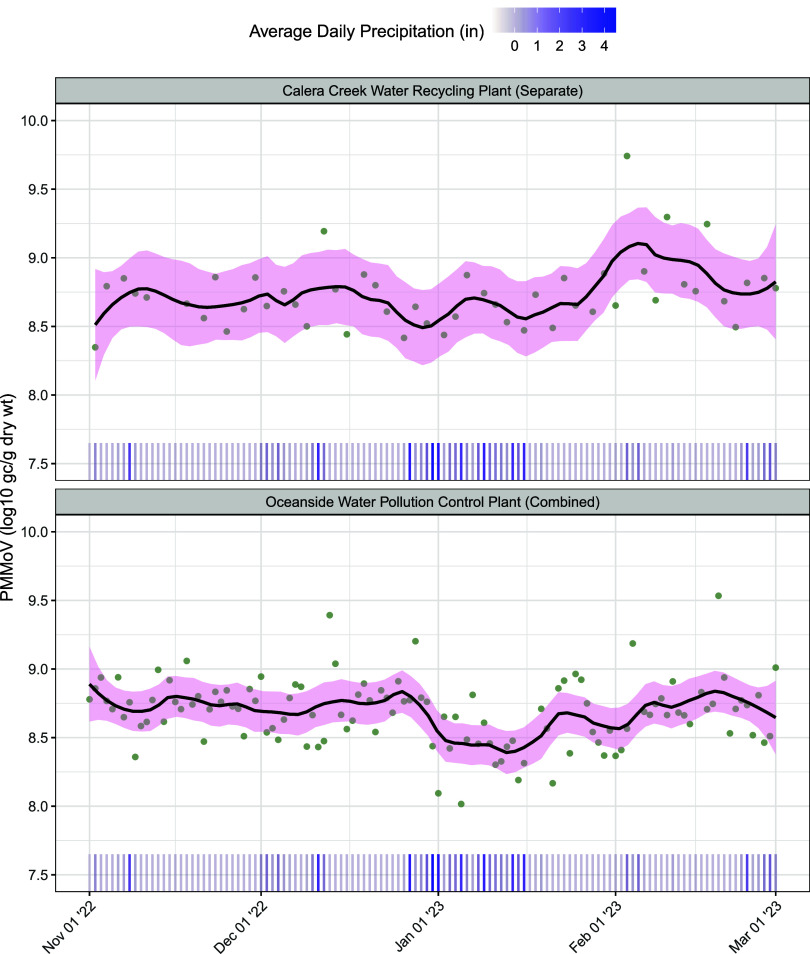
PMMoV concentration remains constant at Calera Creek (separated system) and decreases at Oceanside (combined system) over a period of increased precipitation. Black line is loess smoother with 95% confidence interval. Green points show observed concentrations for available dates. Rug shows average daily precipitation. (gene copies = gc; gram = g; weight = wt; inches = in).

PMMoV concentration drops on days of high precipitation for sites with combined sewer systems. Calera Creek Water Recycling Plant and Oceanside Water Pollution Control Plant are both located in the Bay Area of central California and serve San Mateo County. Due to their proximity, the sewer catchments of both sites experience similar levels of precipitation. However, during a period of higher precipitation, PMMoV concentration at Oceanside, which has a combined sewer system, decreased while it remained relatively constant at Calera Creek, which has a separated system (Figure 5).

When considering all sites together as in the detailed median model, the coefficient for precipitation is statistically significant (β = −7.58 × 10^–2^; *p* = 7.11 × 10^–11^). The coefficient on the sewer type term is negative, while that for the interaction between precipitation and sewer type is positive (Table S2). Moreover, both of these coefficients are not statistically significant, potentially due to the smaller sample size of combined sewer systems in our data set. The coefficient signs imply that greater levels of precipitation are associated with lower concentrations of PMMoV; sites with combined sewer systems experience lower concentrations compared to those with separated systems; and a combined sewer system modifies the effect of precipitation such that PMMoV decreases less.

The attenuation of the effect of precipitation by combined sewer types contrasts with some prior results. Greater amounts of precipitation can lead to dilution effects from groundwater infiltration,^5^ thus leading to lower measured concentrations of viral nucleic acids. In addition, sewer system type mediates the entrance of precipitation into a wastewater treatment plant by allowing dilution by runoff. Goitom et al.^46^ reported a negative association between PMMoV concentration and precipitation for one site included in their study, which they proposed was due to the combined sewers in the served city. However, it has also been suggested that periods of high precipitation can lead to higher viral concentrations, potentially due to the scouring of solid material in the sewers by the higher flow rates or due to the expedited travel time which would reduce degradation of genetic material.^46,48^ This may provide an explanation for why the coefficient for the interaction term between precipitation and sewer type is positive and for why not all sites have negative coefficients for precipitation in the individual Bayesian model fits (Table S6).

## 4 Conclusions

We describe the majority of the variation in PMMoV concentration across a large sample of United States sewer treatment plants using spatiotemporal factors. Our work provides insights into the temporal and geographical trends in PMMoV concentration, showing that it has high levels of variation both within and across sites. Differences across sites may be due to features of the wastewater matrix or sewer system at different facilities, while differences within sites may be due to weather changes or variation in pepper consumption.

Some site-to-site differences and the changes associated with fluctuating precipitation levels suggest PMMoV normalization is effective at accounting for many in-human and in-sewer effects. Furthermore, if future work should show that there is a relationship between PMMoV concentration and distance from the processing laboratory or time between collection and processing, PMMoV may also be able to adjust for transportation-related in-lab effects in cases when viral RNA degradation may be a concern.

However, other longitudinal variation may negatively impact its performance as a normalizer. For example, a location in the southwest US and a location in the northeast US experiencing similar burdens of disease could see large differences in normalized concentrations due to the fact that southwestern sites have, on average, higher concentrations of PMMoV. We hypothesize that this trend may be due to patterns in diet, and future work should consider incorporating information about pepper product consumption in order to further improve correlations.

Our work has several limitations that may affect our findings and their generalizability. These include having a small number of sites with combined sewer systems, an overrepresentation of data from California sites, and no data from outside the COVID-19 pandemic. Quantification was from only solid samples and done at the same laboratory, which enabled us to better investigate in-human and in-sewer effects by reducing confounding from in-lab effects related to variability in sampling or protocol. Consequently, we did not investigate these, but others have found PMMoV to be useful in adjusting for between-sample variation from laboratory processing.^49^ Future studies should examine whether these results hold for different sampling methods (grab vs composite, time- vs flow-proportional, liquid vs solid), which have been shown to affect viral concentrations.^5^ Replication of this study with longer time series and more participating treatment facilities would provide additional insights. However, data sets of this size containing raw concentrations rather than smoothed, prenormalized values or summary statistics are not publicly available.

In the case of SARS-CoV-2, past research shows evidence that normalizing wastewater concentrations of viral genetic material can improve correlations with reported COVID-19 cases across locations and somewhat over time,^8^ making data processing an important component of WBE. Awareness of variation in PMMoV and research into how best to correct for its geographic trend may help to improve these correlations further, thereby enhancing the benefits and accuracy of wastewater-based epidemiology methods.

## Supporting Information Available

Code repository; complete list of R packages used in analysis; distance model specification; illustration of the component effects in the Bayesian median models; autocorrelation analyses; tables for all model fits; site name abbreviations table; and site summary statistics (PDF).
