# Supplementary material for: Spatiotemporal Variability of the Pepper Mild Mottle Virus Biomarker in Wastewater
Source: ACS ES T Water. 2024 Dec 16;5(1):341–50. doi: 10.1021/acsestwater.4c00866 (PMC11731321; doi:10.1021/acsestwater.4c00866)
Supplement: Supplementary file 1 — ew4c00866_si_001.pdf [file ew4c00866_si_001.pdf]

Supplementary Information: Spatiotemporal Variability of the Pepper Mild Mottle Virus Biomarker in Wastewater

AnnaElaine L. Rosengart,[*,†] Amanda L. Bidwell,[‡] Marlene K. Wolfe,[¶] Alexandria B. Boehm,[‡] and F. William Townes[†]

†*Department of Statistics & Data Science, Dietrich College of Humanities and Social Sciences, Carnegie Mellon University, Pittsburgh, PA 15213, United States*

‡*Department of Civil & Environmental Engineering, School of Engineering and Doerr School of Sustainability, Stanford University, Stanford, CA 94305, United States*

¶*Gangarosa Department of Environmental Health, Rollins School of Public Health, Emory University, Atlanta, GA 30322, United States*

E-mail: arosenga@andrew.cmu.edu

All code to reproduce analyses is available free of charge at the accompanying GitHub repository (https://github.com/aerosengart/pmmov-manu.git).

S1 R Packages

The *xlsx* package was used for additional data reading and writing.[S1] Data manipulation was done using the *dplyr* and *magrittr* packages.[S2,S3] ZIP codes for each site were found using the *zipcodeR* package.[S4] Fourier bases were constructed with the *fda* package.[S5]

Linear regression models were fit with the *stats* package.[S6] The simple and detailed median models were fit with the *quantreg* package[S7] using the modified Barrodale and Roberts algorithm,[S8,S9] and standard errors were estimated using the cluster-robust wild bootstrap with grouping defined by site.[S10] The distance model was fit using the *lqmm* package[S11,S12] with distances calculated using the *geosphere* package.[S13] The Bayesian models were fit with the *rstan* package.[S14]

Inverse distance weighting was performed with modified code from the *phylin* package.[S15,S16] Plots were made using the *ggplot2*, *ggrepel*, *scales*, *ggpubr*, *viridis*, and *cowplot* packages.[S17–S22] The *sf* and *concaveman* packages were also used for creating maps.[S23–S25]

S2 Distance Median Model

The distance median model is a modification of the quantile regression model from Section 2.2.2 in the main text that omits latitude and longitude in favor of variables representing a sample's site's distance from the processing laboratory at Verily Life Sciences and El Paso, Texas. Distances were calculated with the Haversine formula using approximate coordinates for Verily Life Sciences in San Francisco, CA, and El Paso, TX, found using Google Maps.[S26,S27] The model has the form:

$$\begin{aligned}\log_{10}\text{PMMoV}_{i,t} = \beta_0 &+ \beta_1 \cdot \text{lab distance}_i + \beta_2 \cdot \text{El Paso distance}_i \\ &+ \beta_3 \cdot \text{prcp}_{i,t} + \beta_4 \cdot \text{sewer}_i + \beta_5 \cdot \text{prcp}_{i,t} \cdot \text{sewer}_i + \beta_6 \cdot \text{site ID}_i \\ &+ \beta_7 \cdot \psi_7^{\sin}(t) + \beta_8 \cdot \psi_7^{\cos}(t) + \beta_9 \cdot \psi_{365.25}^{\sin}(t) + \beta_{10} \cdot \psi_{365.25}^{\cos}(t) + \epsilon_{i,t}\end{aligned} \tag{S1}$$

where $\epsilon_{i,t}$ are independent and identically distributed Laplace random variables with location of 0 and scale of σ.

S3 Component Effects in Bayesian Median Models

To illustrate the effect of each component in the Bayesian median models, we take the Loxahatchee River Environmental Control District site in Florida as an example. We sample from the posterior distribution of the coefficients and use these values to construct the predicted PMMoV concentration by component. The top panel of Figure S1 shows the effect of the yearly time components in addition to the intercept. The middle panel adds the effect of the weekly components, and the bottom panel adds precipitation.

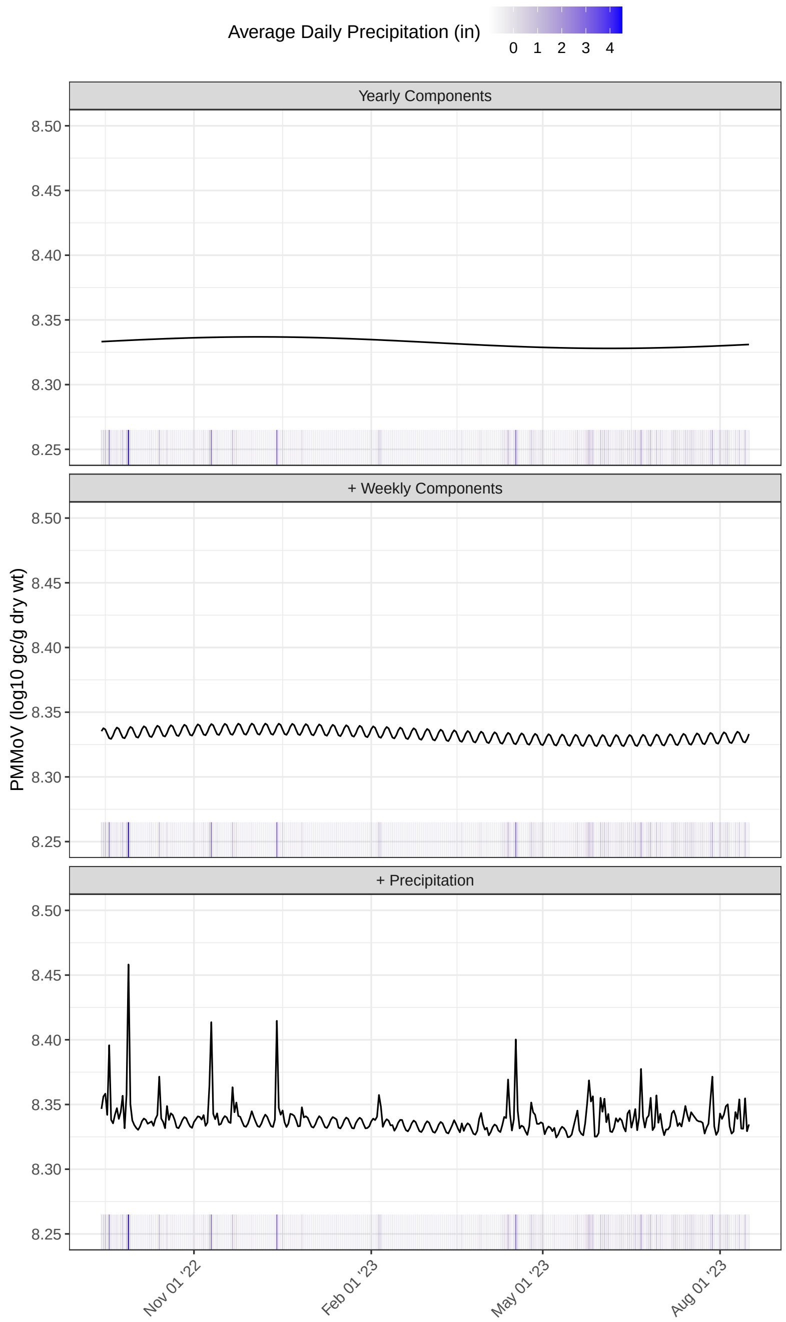

Figure S1: Predicted PMMoV concentration at Loxahatchee River Environmental Control District separated by component effects from one posterior sample. Rug shows average daily precipitation. (gc = gene copies; g = gram; wt = weight; in = inches)

S4 Autocorrelation Analysis

Unlike RNA concentrations for human viruses where autocorrelation might be expected due to the duration of infection and transmission of disease within a community, it is unclear whether PMMoV concentrations should be correlated over time. In theory, consistent and periodic changes occurring in the sewershed or sewer system may affect PMMoV concentrations. For example, samples taken on weekdays may be greater than those taken on the weekends due to commuters to work in urban areas, or weekly grocery shopping may be associated with higher levels of pepper consumption on a particular day of the week. However, this remains speculation without information on dietary habits and the sale of pepper products. Accordingly, the following analysis aims to identify whether autocorrelation is present, rather than its causes.

Figure S2a provides a general summary of the level of autocorrelation in the data. The weighted average autocorrelation is calculated by first augmenting the data by adding rows such that each site's time series is daily. Days with missing observations are filled with an *NA* value. The within-site autocorrelation is then calculated in a similar way to the standard sample autocorrelation;[S28] however, only pairs of observations for which there are data at time t and time $t + k$ (for a lag of k days) are included in the calculations. Observation pair counts for each site and each lag are included in Tables S12 and S13. For the majority of sites, samples were taken several times a week on the same days, so lags of 2-3 days, 7 days, and their multiples had the greatest number of pairs included in the autocorrelation calculations. If there are too few observations such that the lag k autocorrelation cannot be calculated for a given site, then a value of 0 is substituted.

For each value of $k = 0, \ldots, 30$, the weighted average of the lag k autocorrelations for all sites is taken with the weights determined by the number of pairs of observations used to calculate each within-site autocorrelation. Figure S2b performs the same calculations with the residuals of the Bayesian median models, which include weekly components. There is a reduction in autocorrelation, especially at weekly lags (7 days, 14 days, etc.), when

comparing the raw data to the models' residuals.

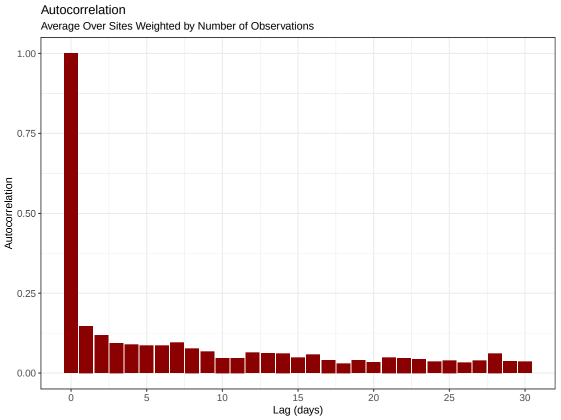

(a) Autocorrelation of $\log_{10}$ PMMoV concentration across all sites exhibits a weekly pattern with higher autocorrelation values for a 7 day lag.

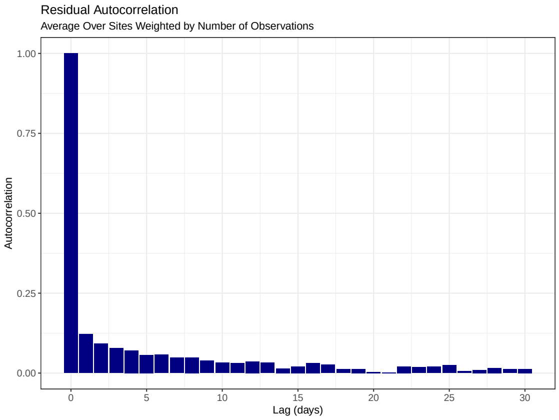

(b) Autocorrelation of residuals from Bayesian median models for all sites.

Figure S2: Summary autocorrelation across all sites.

S5 Model Fits

S5.1 Simple Median Model

Table S1: Coefficient estimates, cluster-robust wild bootstrap standard errors (200 replicates), and associated p-values for the simple median model. Coefficients with bold p-values indicate statistical signficance at level $\alpha = 0.05$.

	Estimate	Std. Error	p-value
Intercept	7.411	0.236	$< \mathbf{1.000 \times 10^{-15}}$
Lat.	-9.764×10^{-5}	5.139×10^{-3}	0.985
Lng.	-1.286×10^{-2}	1.067×10^{-3}	$< \mathbf{1.000 \times 10^{-15}}$

S5.2 Detailed Median Model

Table S2: Coefficient estimates, cluster-robust wild bootstrap standard errors (200 replicates), and associated p-values for the detailed median model. Bold p-values indicate statistical signficance at level $\alpha = 0.05$.

	Estimate	Std. Error	p-value
Intercept	7.412	0.218	$< \mathbf{1.000 \times 10^{-15}}$
Lat.	7.750×10^{-4}	4.773×10^{-3}	0.871
Lng.	-1.278×10^{-2}	1.014×10^{-3}	$< \mathbf{1.000 \times 10^{-15}}$
Avg. Prcp.	-7.578×10^{-2}	1.162×10^{-2}	$\mathbf{7.107 \times 10^{-11}}$
Sewer	-5.661×10^{-2}	4.972×10^{-2}	0.255
Avg. Prcp./Sewer	1.315×10^{-2}	2.964×10^{-2}	0.657
$\psi_{365.25}^{\sin}$	-0.194	9.989×10^{-2}	5.202×10^{-2}
$\psi_{365.25}^{\cos}$	-0.371	0.104	$\mathbf{3.745 \times 10^{-4}}$
$\psi_{7}^{\sin}$	2.460×10^{-2}	1.727×10^{-2}	0.154
$\psi_{7}^{\cos}$	-1.816×10^{-2}	1.282×10^{-2}	0.157

S5.3 Distance Median Model

Table S3: Coefficient estimates, bootstrap standard errors (100 replicates), and associated p-values for the distance median model. Bold p-values indicate statistical signficance at level $\alpha = 0.05$.

	Estimate	Std. Error	p-value
Intercept	9.139	8.309×10^{-3}	$< \mathbf{1.000 \times 10^{-15}}$
Dist. to Lab	-1.259×10^{-7}	2.151×10^{-9}	$< \mathbf{1.000 \times 10^{-15}}$
Dist. to El Paso	-8.778×10^{-8}	6.200×10^{-9}	$< \mathbf{1.000 \times 10^{-15}}$
Avg. Prcp.	-7.224×10^{-2}	8.203×10^{-3}	$\mathbf{4.371 \times 10^{-14}}$
Sewer	-4.639×10^{-2}	4.837×10^{-3}	$< \mathbf{1.000 \times 10^{-15}}$
Avg. Prcp./Sewer	-2.087×10^{-2}	2.018×10^{-2}	0.303
$\psi_{365.25}^{\sin}$	-0.227	4.254×10^{-2}	$\mathbf{6.226 \times 10^{-7}}$
$\psi_{365.25}^{\cos}$	-0.432	4.144×10^{-2}	$< \mathbf{1.000 \times 10^{-15}}$
$\psi_{7}^{\sin}$	3.288×10^{-2}	4.721×10^{-3}	$\mathbf{3.652 \times 10^{-10}}$
$\psi_{7}^{\cos}$	-1.169×10^{-2}	4.931×10^{-3}	$\mathbf{1.966 \times 10^{-2}}$

S5.4 Variance Decomposition Model

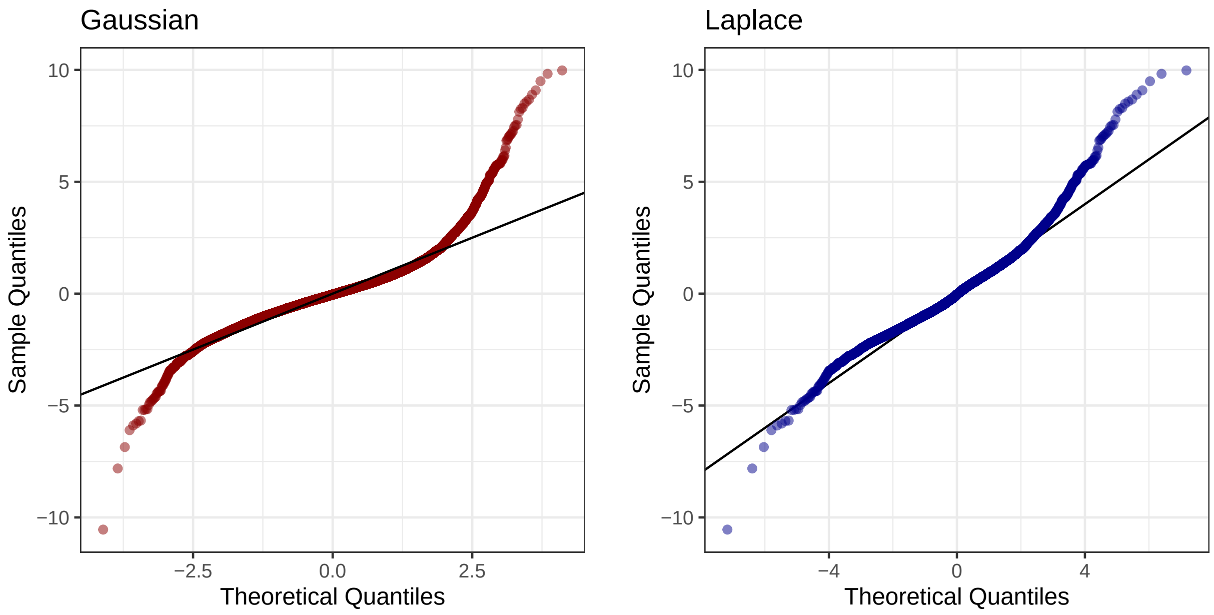

Figure S3: Sample quantiles of standardized residuals of the variance decomposition model against theoretical quantiles of standard Gaussian distribution (left) and standard Laplace distribution (right).

Coefficient estimates for the variance decomposition model are omitted due to the departure from the assumed Gaussianity of errors (Figure S3). Analysis of R^2 remains reasonable as it does not rely upon distributional assumptions.

S5.5 Bayesian Median Models

Table S4: Mean, Monte Carlo standard error, median, 95% credible interval, effective sample size, and $\hat{R}$ for the scale parameter σ calculated over 4,000 posterior samples.

Site	Mean	MCSE	Median	95% CI	ESS	$\hat{R}$
Akron	0.166	4.176×10^{-4}	0.164	$(0.133, 0.208)$	2.138×10^{3}	1.000
Altamonte Springs	0.145	3.105×10^{-4}	0.144	$(0.120, 0.176)$	2.009×10^{3}	1.002
Ann Arbor	0.207	3.449×10^{-4}	0.206	$(0.178, 0.239)$	2.032×10^{3}	1.002
Aquia	8.175×10^{-2}	2.089×10^{-4}	8.111×10^{-2}	$(6.436 \times 10^{-2}, 0.103)$	2.196×10^{3}	1.002
Archie Elledge	0.223	4.127×10^{-4}	0.222	$(0.188, 0.261)$	2.066×10^{3}	1.002
Bangor	0.334	1.321×10^{-3}	0.325	$(0.226, 0.489)$	2.681×10^{3}	1.001
Bayshore	0.163	6.060×10^{-4}	0.162	$(0.121, 0.218)$	1.651×10^{3}	1.001
Big Creek	0.215	3.410×10^{-4}	0.214	$(0.185, 0.248)$	2.230×10^{3}	1.001
Boege Alvarado (Fremont)	0.189	5.084×10^{-4}	0.186	$(0.149, 0.238)$	2.044×10^{3}	1.000
Boege Alvarado (Newark)	0.231	5.830×10^{-4}	0.229	$(0.182, 0.289)$	2.118×10^{3}	0.999

Table S4: Mean, Monte Carlo standard error, median, 95% credible interval, effective sample size, and $\hat{R}$ for the scale parameter σ calculated over 4,000 posterior samples.

Site	Mean	MCSE	Median	95% CI	ESS	$\hat{R}$
Boege Alvarado (Union City)	0.166	4.044×10^{-4}	0.165	$(0.130, 0.209)$	2.390×10^{3}	1.000
Brunswick	0.136	2.857×10^{-4}	0.135	$(0.112, 0.165)$	2.241×10^{3}	1.001
CODIGA	0.334	2.969×10^{-4}	0.333	$(0.307, 0.363)$	2.249×10^{3}	1.000
Cahaba River	0.159	3.015×10^{-4}	0.158	$(0.133, 0.189)$	2.226×10^{3}	1.001
Calera Creek	0.180	3.583×10^{-4}	0.179	$(0.151, 0.217)$	2.135×10^{3}	1.001
Camp Creek	0.212	3.332×10^{-4}	0.212	$(0.183, 0.245)$	2.278×10^{3}	1.001
Capital Region	0.193	3.384×10^{-4}	0.192	$(0.165, 0.227)$	2.122×10^{3}	1.004
Carmel	0.133	4.419×10^{-4}	0.131	$(9.810 \times 10^{-2}, 0.180)$	2.260×10^{3}	1.001
Central Contra Costa	0.131	2.249×10^{-4}	0.130	$(0.114, 0.151)$	1.838×10^{3}	1.000
Central Marin	0.207	4.326×10^{-4}	0.205	$(0.169, 0.255)$	2.564×10^{3}	1.000
Central Marin (W Railroad)	0.231	4.809×10^{-4}	0.229	$(0.189, 0.283)$	2.461×10^{3}	1.001
Central Valley	0.118	2.403×10^{-4}	0.118	$(9.906 \times 10^{-2}, 0.141)$	2.048×10^{3}	1.000
Clark County	0.173	5.035×10^{-4}	0.171	$(0.133, 0.225)$	2.219×10^{3}	1.001
Clinton	7.343×10^{-2}	2.069×10^{-4}	7.275×10^{-2}	$(5.958 \times 10^{-2}, 9.088 \times 10^{-2})$	1.481×10^{3}	1.000
Coastal	0.187	4.267×10^{-4}	0.185	$(0.154, 0.228)$	1.941×10^{3}	1.005
Coeur d'Alene	0.151	2.224×10^{-4}	0.151	$(0.132, 0.172)$	2.131×10^{3}	1.001
Coralville	0.182	4.679×10^{-4}	0.181	$(0.147, 0.225)$	1.852×10^{3}	1.003
Cumberland	0.211	6.051×10^{-4}	0.209	$(0.162, 0.271)$	2.089×10^{3}	1.001
DELCORA	0.163	3.252×10^{-4}	0.162	$(0.135, 0.196)$	2.308×10^{3}	0.999
Davis	0.140	1.338×10^{-4}	0.140	$(0.129, 0.151)$	1.745×10^{3}	1.003
Deer Island	0.137	3.007×10^{-4}	0.136	$(0.112, 0.168)$	2.154×10^{3}	1.000
Dillman Road	0.184	3.556×10^{-4}	0.183	$(0.156, 0.218)$	1.968×10^{3}	1.000
Dover	0.179	4.137×10^{-4}	0.179	$(0.147, 0.218)$	1.898×10^{3}	1.003
Duck Creek	0.116	1.917×10^{-4}	0.116	$(9.875 \times 10^{-2}, 0.135)$	2.411×10^{3}	1.001
E.W. Blom Point Loma	0.192	3.127×10^{-4}	0.191	$(0.164, 0.225)$	2.434×10^{3}	1.001
East Bay	0.198	2.934×10^{-4}	0.197	$(0.175, 0.224)$	1.923×10^{3}	1.001
Eastern	0.193	2.726×10^{-4}	0.192	$(0.168, 0.221)$	2.433×10^{3}	1.001
Ellis Creek	0.158	2.836×10^{-4}	0.157	$(0.133, 0.187)$	2.363×10^{3}	1.000
Esparto	0.261	5.320×10^{-4}	0.259	$(0.215, 0.318)$	2.390×10^{3}	0.999
Essex	0.133	4.177×10^{-4}	0.131	$(0.103, 0.172)$	1.792×10^{3}	1.003
Fairfield-Suisun	0.124	2.290×10^{-4}	0.123	$(0.105, 0.147)$	2.118×10^{3}	1.000
Five Mile Creek	0.155	2.952×10^{-4}	0.154	$(0.129, 0.185)$	2.337×10^{3}	1.000
Gainesville	0.188	4.074×10^{-4}	0.187	$(0.151, 0.231)$	2.589×10^{3}	1.000
Garland Rowlett Creek	0.162	2.379×10^{-4}	0.161	$(0.142, 0.184)$	2.043×10^{3}	1.001
Glenbard	0.156	2.986×10^{-4}	0.155	$(0.132, 0.185)$	1.935×10^{3}	1.002
Grandville	0.124	2.534×10^{-4}	0.124	$(0.103, 0.149)$	2.188×10^{3}	1.001
Hagerstown	0.231	4.927×10^{-4}	0.229	$(0.190, 0.281)$	2.307×10^{3}	1.000
Hall Street	0.162	3.243×10^{-4}	0.161	$(0.136, 0.194)$	2.102×10^{3}	1.000
Hamlin	0.218	4.722×10^{-4}	0.216	$(0.180, 0.265)$	2.109×10^{3}	1.001
Harrison	0.201	6.219×10^{-4}	0.198	$(0.151, 0.269)$	2.273×10^{3}	1.001
Hillsville	8.930×10^{-2}	1.983×10^{-4}	8.859×10^{-2}	$(7.302 \times 10^{-2}, 0.109)$	2.244×10^{3}	1.000
Hollister	0.197	3.585×10^{-4}	0.196	$(0.165, 0.233)$	2.339×10^{3}	1.002
Hollywood Road	0.278	5.799×10^{-4}	0.276	$(0.229, 0.338)$	2.281×10^{3}	1.001
Hyperion	0.182	3.085×10^{-4}	0.181	$(0.155, 0.214)$	2.366×10^{3}	1.001
JB Latham	0.198	3.656×10^{-4}	0.196	$(0.163, 0.240)$	2.867×10^{3}	1.001
Jackson	0.111	1.801×10^{-4}	0.111	$(9.614 \times 10^{-2}, 0.128)$	1.975×10^{3}	1.001
Jeffersonville	0.194	3.963×10^{-4}	0.192	$(0.161, 0.232)$	2.079×10^{3}	1.000
John M. Asplund	0.239	9.478×10^{-4}	0.234	$(0.167, 0.340)$	2.210×10^{3}	1.001
Johnnie Mosley	0.175	4.155×10^{-4}	0.174	$(0.144, 0.213)$	1.787×10^{3}	1.000
Johns Creek	0.196	2.957×10^{-4}	0.196	$(0.168, 0.228)$	2.622×10^{3}	1.000
Joint	0.146	2.143×10^{-4}	0.146	$(0.129, 0.166)$	1.897×10^{3}	1.002
Kansas City	0.172	3.561×10^{-4}	0.171	$(0.140, 0.211)$	2.586×10^{3}	1.002

Table S4: Mean, Monte Carlo standard error, median, 95% credible interval, effective sample size, and $\hat{R}$ for the scale parameter σ calculated over 4,000 posterior samples.

Site	Mean	MCSE	Median	95% CI	ESS	$\hat{R}$
Kaw Point	0.149	3.159×10^{-4}	0.148	$(0.121, 0.182)$	2.544×10^{3}	1.002
Lancaster	0.214	3.816×10^{-4}	0.212	$(0.180, 0.252)$	2.356×10^{3}	1.000
Lander Street	0.186	4.488×10^{-4}	0.184	$(0.150, 0.230)$	2.048×10^{3}	1.000
Las Gallinas	0.188	3.321×10^{-4}	0.187	$(0.160, 0.220)$	2.153×10^{3}	0.999
Lawrence Kansas	0.138	2.499×10^{-4}	0.138	$(0.117, 0.164)$	2.163×10^{3}	1.001
Little Falls Run	7.839×10^{-2}	1.925×10^{-4}	7.768×10^{-2}	$(6.114 \times 10^{-2}, 9.976 \times 10^{-2})$	2.612×10^{3}	1.000
Little River	0.200	3.285×10^{-4}	0.199	$(0.172, 0.232)$	2.205×10^{3}	1.001
Lompoc	0.213	3.459×10^{-4}	0.212	$(0.182, 0.248)$	2.416×10^{3}	1.002
Los Banos	0.207	4.141×10^{-4}	0.206	$(0.171, 0.250)$	2.448×10^{3}	1.002
Loxahatchee	0.184	3.390×10^{-4}	0.183	$(0.156, 0.216)$	2.197×10^{3}	1.001
MDWASD Central	0.175	4.414×10^{-4}	0.174	$(0.139, 0.218)$	2.029×10^{3}	1.000
MDWASD North	0.260	5.940×10^{-4}	0.259	$(0.210, 0.320)$	2.198×10^{3}	1.001
MDWASD South	0.161	4.170×10^{-4}	0.159	$(0.129, 0.200)$	1.828×10^{3}	1.001
Madera	0.118	3.261×10^{-4}	0.117	$(9.225 \times 10^{-2}, 0.151)$	2.129×10^{3}	1.000
Mankato	0.109	2.027×10^{-4}	0.109	$(9.223 \times 10^{-2}, 0.128)$	2.124×10^{3}	1.001
Markshalltown	0.202	4.125×10^{-4}	0.200	$(0.163, 0.250)$	2.839×10^{3}	1.001
Marlay Taylor	0.165	4.688×10^{-4}	0.164	$(0.132, 0.206)$	1.716×10^{3}	1.000
Merced	0.154	1.845×10^{-4}	0.153	$(0.138, 0.172)$	2.243×10^{3}	1.001
Mid-Coastside	0.198	3.098×10^{-4}	0.198	$(0.171, 0.228)$	2.191×10^{3}	1.000
Modesto's Sutter	0.136	2.005×10^{-4}	0.136	$(0.122, 0.153)$	1.630×10^{3}	1.002
Montpelier	0.150	3.688×10^{-4}	0.149	$(0.118, 0.190)$	2.624×10^{3}	1.000
Monterey One	0.209	4.416×10^{-4}	0.208	$(0.173, 0.253)$	2.114×10^{3}	0.999
Morris Forman	0.124	3.318×10^{-4}	0.123	$(9.860 \times 10^{-2}, 0.158)$	2.110×10^{3}	1.001
Mt. Pleasant	0.182	5.249×10^{-4}	0.180	$(0.138, 0.241)$	2.546×10^{3}	1.004
Muscatine	0.203	4.121×10^{-4}	0.201	$(0.168, 0.246)$	2.435×10^{3}	1.001
Norhtwest Water	0.186	2.830×10^{-4}	0.186	$(0.160, 0.214)$	2.376×10^{3}	1.000
North Water	0.143	3.085×10^{-4}	0.143	$(0.119, 0.172)$	1.858×10^{3}	1.000
Novato	0.174	2.734×10^{-4}	0.173	$(0.151, 0.202)$	2.306×10^{3}	1.002
Ocean	0.198	7.661×10^{-4}	0.194	$(0.138, 0.279)$	2.201×10^{3}	1.003
Oceanside	0.148	1.283×10^{-4}	0.148	$(0.138, 0.159)$	1.737×10^{3}	1.002
Ottumwa	0.171	3.531×10^{-4}	0.170	$(0.140, 0.209)$	2.389×10^{3}	1.001
Palo Alto	0.128	1.052×10^{-4}	0.128	$(0.120, 0.138)$	1.877×10^{3}	1.000
Parker North	0.194	3.298×10^{-4}	0.192	$(0.168, 0.223)$	1.814×10^{3}	1.001
Parker South	0.206	3.200×10^{-4}	0.206	$(0.178, 0.238)$	2.289×10^{3}	1.001
Paso Robles	0.132	1.773×10^{-4}	0.132	$(0.116, 0.150)$	2.297×10^{3}	1.000
Passaic Valley	0.188	3.437×10^{-4}	0.187	$(0.160, 0.221)$	2.101×10^{3}	1.003
Penacook	0.174	3.481×10^{-4}	0.173	$(0.146, 0.209)$	2.019×10^{3}	1.002
Portland	0.155	3.078×10^{-4}	0.153	$(0.128, 0.188)$	2.549×10^{3}	1.001
Provo City	0.136	2.959×10^{-4}	0.135	$(0.111, 0.166)$	2.242×10^{3}	1.002
RM Clayton	0.185	3.711×10^{-4}	0.184	$(0.154, 0.222)$	2.252×10^{3}	1.000
Red Wing	9.857×10^{-2}	4.078×10^{-4}	9.670×10^{-2}	$(6.937 \times 10^{-2}, 0.141)$	2.046×10^{3}	1.000
Regional	0.176	3.731×10^{-4}	0.175	$(0.144, 0.215)$	2.364×10^{3}	1.003
Regional No. 1	0.161	2.610×10^{-4}	0.160	$(0.139, 0.185)$	1.971×10^{3}	1.000
River Road	0.191	4.030×10^{-4}	0.191	$(0.157, 0.231)$	2.257×10^{3}	1.000
Riverside	0.209	4.616×10^{-4}	0.208	$(0.170, 0.257)$	2.364×10^{3}	1.000
Rochester	0.124	2.311×10^{-4}	0.123	$(0.104, 0.149)$	2.596×10^{3}	1.002
SJRA No. 1	0.172	4.147×10^{-4}	0.171	$(0.137, 0.217)$	2.450×10^{3}	1.000
SJRA No. 2	0.177	4.540×10^{-4}	0.176	$(0.141, 0.224)$	2.195×10^{3}	1.000
SJRA No. 3	0.191	5.107×10^{-4}	0.189	$(0.153, 0.239)$	1.960×10^{3}	1.002
Sacramento	9.782×10^{-2}	8.091×10^{-5}	9.771×10^{-2}	$(9.155 \times 10^{-2}, 0.105)$	1.818×10^{3}	1.000
Salina	0.115	2.692×10^{-4}	0.114	$(9.313 \times 10^{-2}, 0.141)$	2.191×10^{3}	1.001
San Francisco	0.121	1.239×10^{-4}	0.121	$(0.110, 0.133)$	2.112×10^{3}	1.001

Table S4: Mean, Monte Carlo standard error, median, 95% credible interval, effective sample size, and $\hat{R}$ for the scale parameter σ calculated over 4,000 posterior samples.

Site	Mean	MCSE	Median	95% CI	ESS	$\hat{R}$
San Jose-Santa Clara	0.103	1.131×10^{-4}	0.103	$(9.622 \times 10^{-2}, 0.110)$	1.053×10^{3}	1.003
San Leandro	0.228	4.317×10^{-4}	0.227	$(0.192, 0.272)$	2.213×10^{3}	1.000
San Mateo & Estero	0.182	3.229×10^{-4}	0.181	$(0.156, 0.212)$	2.051×10^{3}	1.001
Santa Cruz (City)	0.198	2.931×10^{-4}	0.197	$(0.174, 0.228)$	2.230×10^{3}	1.001
Santa Cruz (County)	0.171	2.883×10^{-4}	0.171	$(0.148, 0.196)$	1.764×10^{3}	1.002
Santa Rosa	0.117	2.238×10^{-4}	0.116	$(9.980 \times 10^{-2}, 0.138)$	1.880×10^{3}	1.002
Sausalito-Marin	0.181	3.001×10^{-4}	0.180	$(0.153, 0.213)$	2.737×10^{3}	1.001
Seaford	0.128	3.366×10^{-4}	0.126	$(0.102, 0.162)$	2.022×10^{3}	1.003
Silicon Valley	0.176	1.371×10^{-4}	0.176	$(0.164, 0.188)$	2.009×10^{3}	1.000
Somerset Raritan	0.227	8.420×10^{-4}	0.222	$(0.163, 0.319)$	2.191×10^{3}	1.001
Soscol	0.115	2.150×10^{-4}	0.115	$(9.626 \times 10^{-2}, 0.137)$	2.296×10^{3}	1.000
South Bay	0.132	5.426×10^{-4}	0.128	$(9.277 \times 10^{-2}, 0.188)$	2.032×10^{3}	0.999
South Bend	0.183	3.390×10^{-4}	0.182	$(0.154, 0.215)$	2.145×10^{3}	1.002
South Burlington	9.831×10^{-2}	4.328×10^{-4}	9.615×10^{-2}	$(7.040 \times 10^{-2}, 0.138)$	1.662×10^{3}	1.002
South Columbus	0.148	3.003×10^{-4}	0.147	$(0.123, 0.176)$	2.117×10^{3}	1.000
South County	0.202	1.668×10^{-4}	0.202	$(0.188, 0.217)$	1.956×10^{3}	1.002
South Laredo	0.212	4.426×10^{-4}	0.211	$(0.173, 0.263)$	2.615×10^{3}	1.000
South Monmouth	0.178	3.595×10^{-4}	0.177	$(0.147, 0.217)$	2.434×10^{3}	1.000
South River	0.173	3.732×10^{-4}	0.172	$(0.143, 0.210)$	2.019×10^{3}	1.000
South Water	0.174	2.765×10^{-4}	0.173	$(0.152, 0.201)$	2.172×10^{3}	1.000
Southern Marin	0.113	2.332×10^{-4}	0.112	$(9.207 \times 10^{-2}, 0.137)$	2.423×10^{3}	1.001
St. Cloud	0.158	4.874×10^{-4}	0.155	$(0.121, 0.208)$	2.027×10^{3}	1.001
Sunnyvale	0.124	1.005×10^{-4}	0.124	$(0.116, 0.133)$	1.744×10^{3}	1.000
Traverse City	0.178	4.502×10^{-4}	0.177	$(0.145, 0.221)$	1.898×10^{3}	1.001
Turkey Creek	0.209	4.156×10^{-4}	0.208	$(0.175, 0.248)$	2.057×10^{3}	0.999
Turlock	0.262	5.394×10^{-4}	0.260	$(0.217, 0.316)$	2.270×10^{3}	1.001
Upper Blackstone	0.180	4.836×10^{-4}	0.178	$(0.139, 0.231)$	2.332×10^{3}	1.000
Utoy Creek	0.171	3.777×10^{-4}	0.170	$(0.142, 0.206)$	1.877×10^{3}	1.000
Vallejo	0.233	5.168×10^{-4}	0.231	$(0.197, 0.278)$	1.604×10^{3}	1.003
Valley	0.165	3.242×10^{-4}	0.165	$(0.140, 0.194)$	1.867×10^{3}	1.002
Valley Creek	0.159	2.588×10^{-4}	0.158	$(0.137, 0.186)$	2.331×10^{3}	1.000
Village Creek	0.201	3.380×10^{-4}	0.200	$(0.171, 0.235)$	2.314×10^{3}	1.001
Warren	0.188	3.486×10^{-4}	0.187	$(0.158, 0.222)$	2.204×10^{3}	1.003
Weaton	0.131	2.399×10^{-4}	0.131	$(0.111, 0.154)$	2.151×10^{3}	1.001
West Boise	0.175	3.965×10^{-4}	0.174	$(0.143, 0.216)$	2.255×10^{3}	1.001
West County	0.216	3.613×10^{-4}	0.216	$(0.183, 0.252)$	2.328×10^{3}	1.001
Wheeling	0.101	3.354×10^{-4}	9.978×10^{-2}	$(7.483 \times 10^{-2}, 0.135)$	2.069×10^{3}	1.000
Wichita Falls	0.127	3.182×10^{-4}	0.126	$(0.104, 0.154)$	1.629×10^{3}	1.001
Windsor	0.225	5.085×10^{-4}	0.224	$(0.184, 0.275)$	2.197×10^{3}	1.001
Winters	0.232	4.710×10^{-4}	0.231	$(0.191, 0.283)$	2.477×10^{3}	1.000
Wolcott	0.217	5.008×10^{-4}	0.215	$(0.178, 0.265)$	2.117×10^{3}	1.000
Woodland	0.229	4.463×10^{-4}	0.228	$(0.191, 0.278)$	2.457×10^{3}	1.000
Yankton	0.150	4.915×10^{-4}	0.147	$(0.111, 0.201)$	2.267×10^{3}	1.001
York	0.169	3.932×10^{-4}	0.167	$(0.135, 0.210)$	2.386×10^{3}	0.999
Youngstown	0.163	3.568×10^{-4}	0.162	$(0.132, 0.198)$	2.287×10^{3}	1.000
Zacate Creek	0.220	4.867×10^{-4}	0.218	$(0.179, 0.271)$	2.414×10^{3}	0.999

Table S5: Mean, Monte Carlo standard error, median, 95% credible interval, effective sample size, and $\hat{R}$ for intercept calculated over 4,000 posterior samples. Bolded intervals do not contain 0.

Site	Mean	MCSE	Median	95% CI	ESS	$\hat{R}$
Akron	8.487	4.242×10^{-4}	8.487	(**8.452**, **8.523**)	1.874×10^3	1.000
Altamonte Springs	8.489	4.079×10^{-4}	8.489	(**8.454**, **8.522**)	1.790×10^3	1.003
Ann Arbor	8.654	3.908×10^{-4}	8.655	(**8.618**, **8.688**)	2.128×10^3	1.001
Aquia	8.635	2.441×10^{-4}	8.635	(**8.613**, **8.659**)	2.191×10^3	1.003
Archie Elledge	8.602	4.779×10^{-4}	8.602	(**8.560**, **8.645**)	2.129×10^3	1.002
Bangor	7.892	1.964×10^{-3}	7.884	(**7.725**, **8.083**)	2.208×10^3	1.001
Bayshore	8.493	5.867×10^{-4}	8.494	(**8.437**, **8.545**)	2.177×10^3	1.000
Big Creek	8.641	3.836×10^{-4}	8.641	(**8.608**, **8.674**)	1.935×10^3	1.000
Boege Alvarado (Fremont)	8.702	5.112×10^{-4}	8.701	(**8.655**, **8.754**)	2.367×10^3	1.000
Boege Alvarado (Newark)	8.755	5.700×10^{-4}	8.755	(**8.701**, **8.812**)	2.506×10^3	1.000
Boege Alvarado (Union City)	8.713	4.796×10^{-4}	8.713	(**8.668**, **8.756**)	2.217×10^3	1.000
Brunswick	8.163	3.212×10^{-4}	8.163	(**8.135**, **8.193**)	2.247×10^3	1.000
CODIGA	8.732	3.673×10^{-4}	8.732	(**8.698**, **8.764**)	2.041×10^3	1.000
Cahaba River	8.576	3.451×10^{-4}	8.576	(**8.543**, **8.607**)	2.301×10^3	1.001
Calera Creek	8.654	4.599×10^{-4}	8.654	(**8.613**, **8.694**)	2.085×10^3	1.005
Camp Creek	8.450	4.622×10^{-4}	8.450	(**8.410**, **8.487**)	1.840×10^3	1.000
Capital Region	8.491	3.861×10^{-4}	8.491	(**8.457**, **8.524**)	2.057×10^3	0.999
Carmel	8.806	4.906×10^{-4}	8.805	(**8.763**, **8.855**)	2.254×10^3	0.999
Central Contra Costa	8.946	2.164×10^{-4}	8.946	(**8.925**, **8.966**)	2.340×10^3	1.000
Central Marin	8.614	5.160×10^{-4}	8.614	(**8.564**, **8.663**)	2.458×10^3	1.001
Central Marin (W Railroad)	8.923	5.895×10^{-4}	8.924	(**8.870**, **8.974**)	2.035×10^3	1.003
Central Valley	8.975	2.679×10^{-4}	8.976	(**8.951**, **8.999**)	2.097×10^3	1.004
Clark County	8.644	6.364×10^{-4}	8.644	(**8.589**, **8.699**)	1.946×10^3	1.003
Clinton	8.112	2.220×10^{-4}	8.112	(**8.095**, **8.131**)	1.727×10^3	1.001
Coastal	8.791	5.227×10^{-4}	8.791	(**8.746**, **8.839**)	2.043×10^3	1.001
Coeur d'Alene	8.646	2.688×10^{-4}	8.646	(**8.624**, **8.671**)	1.968×10^3	1.000
Coralville	8.611	5.428×10^{-4}	8.611	(**8.564**, **8.658**)	2.033×10^3	1.000
Cumberland	8.762	8.807×10^{-4}	8.763	(**8.692**, **8.826**)	1.477×10^3	1.001
DELCORA	8.319	5.617×10^{-4}	8.319	(**8.275**, **8.362**)	1.608×10^3	0.999
Davis	8.927	1.243×10^{-4}	8.927	(**8.915**, **8.939**)	2.422×10^3	1.000
Deer Island	8.420	3.351×10^{-4}	8.420	(**8.389**, **8.451**)	2.173×10^3	0.999
Dillman Road	8.495	4.187×10^{-4}	8.495	(**8.457**, **8.534**)	2.247×10^3	1.000
Dover	8.309	4.710×10^{-4}	8.310	(**8.267**, **8.350**)	2.039×10^3	1.002
Duck Creek	8.843	2.348×10^{-4}	8.843	(**8.822**, **8.866**)	2.245×10^3	1.000
E.W. Blom Point Loma	9.097	4.052×10^{-4}	9.097	(**9.061**, **9.135**)	2.277×10^3	1.000
East Bay	8.770	3.168×10^{-4}	8.770	(**8.742**, **8.799**)	2.180×10^3	1.001
Eastern	8.215	3.207×10^{-4}	8.215	(**8.185**, **8.246**)	2.338×10^3	1.002
Ellis Creek	8.761	3.178×10^{-4}	8.760	(**8.728**, **8.793**)	2.706×10^3	1.001
Esparto	9.049	6.265×10^{-4}	9.049	(**8.995**, **9.105**)	2.108×10^3	1.001
Essex	8.497	3.818×10^{-4}	8.497	(**8.459**, **8.531**)	2.284×10^3	0.999
Fairfield-Suisun	8.947	2.595×10^{-4}	8.947	(**8.923**, **8.972**)	2.327×10^3	1.001
Five Mile Creek	8.446	3.429×10^{-4}	8.445	(**8.414**, **8.477**)	2.193×10^3	1.002
Gainesville	8.791	4.706×10^{-4}	8.791	(**8.749**, **8.836**)	2.209×10^3	1.002
Garland Rowlett Creek	8.982	2.740×10^{-4}	8.982	(**8.957**, **9.007**)	2.162×10^3	1.002
Glenbard	8.635	2.979×10^{-4}	8.634	(**8.609**, **8.662**)	2.098×10^3	1.001
Grandville	8.641	2.977×10^{-4}	8.641	(**8.615**, **8.669**)	2.173×10^3	1.001
Hagerstown	8.446	5.487×10^{-4}	8.446	(**8.391**, **8.499**)	2.488×10^3	1.002
Hall Street	8.267	3.790×10^{-4}	8.267	(**8.233**, **8.301**)	2.077×10^3	1.000
Hamlin	8.116	5.387×10^{-4}	8.116	(**8.067**, **8.172**)	2.418×10^3	1.001
Harrison	8.226	7.033×10^{-4}	8.226	(**8.161**, **8.295**)	2.377×10^3	1.001

Table S5: Mean, Monte Carlo standard error, median, 95% credible interval, effective sample size, and $\hat{R}$ for intercept calculated over 4,000 posterior samples. Bolded intervals do not contain 0.

Site	Mean	MCSE	Median	95% CI	ESS	$\hat{R}$
Hillsville	8.256	2.021×10^{-4}	8.256	$(\mathbf{8.236}, \mathbf{8.274})$	2.310×10^{3}	1.000
Hollister	9.199	4.682×10^{-4}	9.199	$(\mathbf{9.157}, \mathbf{9.244})$	2.223×10^{3}	1.000
Hollywood Road	8.607	6.022×10^{-4}	8.608	$(\mathbf{8.548}, \mathbf{8.663})$	2.342×10^{3}	1.000
Hyperion	8.995	3.906×10^{-4}	8.995	$(\mathbf{8.958}, \mathbf{9.033})$	2.428×10^{3}	1.001
JB Latham	8.765	5.376×10^{-4}	8.765	$(\mathbf{8.716}, \mathbf{8.818})$	2.392×10^{3}	1.001
Jackson	8.560	2.341×10^{-4}	8.560	$(\mathbf{8.540}, \mathbf{8.580})$	1.991×10^{3}	1.002
Jeffersonville	8.301	3.905×10^{-4}	8.301	$(\mathbf{8.264}, \mathbf{8.340})$	2.463×10^{3}	1.002
John M. Asplund	8.477	1.239×10^{-3}	8.478	$(\mathbf{8.367}, \mathbf{8.581})$	1.879×10^{3}	1.002
Johnnie Mosley	8.025	4.277×10^{-4}	8.025	$(\mathbf{7.985}, \mathbf{8.066})$	2.415×10^{3}	1.001
Johns Creek	8.617	4.544×10^{-4}	8.617	$(\mathbf{8.577}, \mathbf{8.657})$	1.990×10^{3}	1.000
Joint	9.131	2.616×10^{-4}	9.131	$(\mathbf{9.107}, \mathbf{9.153})$	2.107×10^{3}	1.001
Kansas City	8.751	4.548×10^{-4}	8.752	$(\mathbf{8.710}, \mathbf{8.791})$	2.088×10^{3}	1.002
Kaw Point	8.802	3.625×10^{-4}	8.802	$(\mathbf{8.765}, \mathbf{8.837})$	2.617×10^{3}	1.000
Lancaster	8.941	4.089×10^{-4}	8.941	$(\mathbf{8.904}, \mathbf{8.978})$	2.127×10^{3}	1.000
Lander Street	8.468	4.795×10^{-4}	8.469	$(\mathbf{8.421}, \mathbf{8.513})$	2.323×10^{3}	1.002
Las Gallinas	8.694	3.804×10^{-4}	8.694	$(\mathbf{8.658}, \mathbf{8.729})$	2.188×10^{3}	1.000
Lawrence Kansas	8.651	2.874×10^{-4}	8.652	$(\mathbf{8.625}, \mathbf{8.676})$	1.969×10^{3}	1.001
Little Falls Run	8.633	2.057×10^{-4}	8.633	$(\mathbf{8.611}, \mathbf{8.653})$	2.591×10^{3}	1.001
Little River	8.331	3.589×10^{-4}	8.331	$(\mathbf{8.295}, \mathbf{8.365})$	2.453×10^{3}	1.000
Lompoc	9.121	4.242×10^{-4}	9.121	$(\mathbf{9.080}, \mathbf{9.160})$	2.361×10^{3}	1.001
Los Banos	9.058	4.670×10^{-4}	9.058	$(\mathbf{9.014}, \mathbf{9.103})$	2.373×10^{3}	1.001
Loxahatchee	8.319	3.779×10^{-4}	8.319	$(\mathbf{8.285}, \mathbf{8.352})$	2.063×10^{3}	1.001
MDWASD Central	8.197	5.367×10^{-4}	8.197	$(\mathbf{8.149}, \mathbf{8.247})$	2.115×10^{3}	1.000
MDWASD North	8.084	6.121×10^{-4}	8.084	$(\mathbf{8.031}, \mathbf{8.137})$	1.967×10^{3}	1.001
MDWASD South	8.278	4.411×10^{-4}	8.279	$(\mathbf{8.237}, \mathbf{8.318})$	2.188×10^{3}	0.999
Madera	9.107	3.701×10^{-4}	9.107	$(\mathbf{9.075}, \mathbf{9.140})$	1.969×10^{3}	1.000
Mankato	8.617	2.275×10^{-4}	8.617	$(\mathbf{8.598}, \mathbf{8.636})$	1.775×10^{3}	1.001
Markshalltown	8.811	6.188×10^{-4}	8.811	$(\mathbf{8.754}, \mathbf{8.868})$	2.218×10^{3}	1.000
Marlay Taylor	8.359	4.412×10^{-4}	8.359	$(\mathbf{8.321}, \mathbf{8.399})$	2.028×10^{3}	1.000
Merced	9.236	2.481×10^{-4}	9.236	$(\mathbf{9.215}, \mathbf{9.256})$	1.842×10^{3}	1.000
Mid-Coastside	8.791	3.704×10^{-4}	8.792	$(\mathbf{8.759}, \mathbf{8.823})$	1.936×10^{3}	1.002
Modesto's Sutter	9.178	1.773×10^{-4}	9.178	$(\mathbf{9.161}, \mathbf{9.195})$	2.426×10^{3}	1.001
Montpelier	8.496	4.506×10^{-4}	8.497	$(\mathbf{8.451}, \mathbf{8.537})$	2.347×10^{3}	1.001
Monterey One	9.135	6.220×10^{-4}	9.135	$(\mathbf{9.079}, \mathbf{9.190})$	2.081×10^{3}	1.001
Morris Forman	8.203	3.922×10^{-4}	8.203	$(\mathbf{8.169}, \mathbf{8.236})$	1.908×10^{3}	1.001
Mt. Pleasant	8.401	7.008×10^{-4}	8.400	$(\mathbf{8.343}, \mathbf{8.464})$	1.919×10^{3}	1.002
Muscatine	8.613	5.134×10^{-4}	8.613	$(\mathbf{8.567}, \mathbf{8.657})$	1.948×10^{3}	1.001
Norhtwest Water	8.266	3.214×10^{-4}	8.266	$(\mathbf{8.234}, \mathbf{8.293})$	2.159×10^{3}	1.001
North Water	8.414	3.606×10^{-4}	8.414	$(\mathbf{8.383}, \mathbf{8.448})$	2.064×10^{3}	1.000
Novato	8.688	3.247×10^{-4}	8.689	$(\mathbf{8.658}, \mathbf{8.718})$	2.275×10^{3}	1.000
Ocean	8.691	8.855×10^{-4}	8.691	$(\mathbf{8.603}, \mathbf{8.783})$	2.533×10^{3}	1.000
Oceanside	8.745	1.239×10^{-4}	8.745	$(\mathbf{8.733}, \mathbf{8.757})$	2.505×10^{3}	1.000
Ottumwa	8.355	4.225×10^{-4}	8.355	$(\mathbf{8.317}, \mathbf{8.392})$	2.055×10^{3}	1.001
Palo Alto	9.010	1.122×10^{-4}	9.010	$(\mathbf{9.000}, \mathbf{9.020})$	2.084×10^{3}	1.002
Parker North	8.602	3.598×10^{-4}	8.602	$(\mathbf{8.571}, \mathbf{8.633})$	1.999×10^{3}	1.000
Parker South	8.530	4.091×10^{-4}	8.530	$(\mathbf{8.494}, \mathbf{8.566})$	2.018×10^{3}	1.001
Paso Robles	9.201	1.941×10^{-4}	9.201	$(\mathbf{9.181}, \mathbf{9.221})$	2.639×10^{3}	1.001
Passaic Valley	8.445	3.605×10^{-4}	8.446	$(\mathbf{8.413}, \mathbf{8.477})$	2.127×10^{3}	1.001
Penacook	8.273	4.247×10^{-4}	8.272	$(\mathbf{8.237}, \mathbf{8.308})$	1.888×10^{3}	1.001
Portland	8.399	3.815×10^{-4}	8.398	$(\mathbf{8.363}, \mathbf{8.438})$	2.366×10^{3}	1.001

Table S5: Mean, Monte Carlo standard error, median, 95% credible interval, effective sample size, and $\hat{R}$ for intercept calculated over 4,000 posterior samples. Bolded intervals do not contain 0.

Site	Mean	MCSE	Median	95% CI	ESS	$\hat{R}$
Provo City	8.897	3.668×10^{-4}	8.896	$(\mathbf{8.864}, \mathbf{8.933})$	2.292×10^{3}	1.001
RM Clayton	8.594	4.577×10^{-4}	8.594	$(\mathbf{8.551}, \mathbf{8.636})$	2.276×10^{3}	1.000
Red Wing	8.656	4.761×10^{-4}	8.656	$(\mathbf{8.614}, \mathbf{8.701})$	2.233×10^{3}	1.000
Regional	8.688	3.641×10^{-4}	8.688	$(\mathbf{8.648}, \mathbf{8.727})$	3.082×10^{3}	1.001
Regional No. 1	9.034	3.602×10^{-4}	9.033	$(\mathbf{9.003}, \mathbf{9.063})$	1.702×10^{3}	1.000
River Road	8.743	4.772×10^{-4}	8.743	$(\mathbf{8.698}, \mathbf{8.792})$	2.493×10^{3}	0.999
Riverside	9.083	5.542×10^{-4}	9.083	$(\mathbf{9.031}, \mathbf{9.137})$	2.334×10^{3}	1.000
Rochester	8.691	2.850×10^{-4}	8.691	$(\mathbf{8.665}, \mathbf{8.718})$	2.251×10^{3}	1.000
SJRA No. 1	8.351	5.380×10^{-4}	8.351	$(\mathbf{8.302}, \mathbf{8.397})$	1.979×10^{3}	1.001
SJRA No. 2	8.347	4.978×10^{-4}	8.347	$(\mathbf{8.301}, \mathbf{8.396})$	2.288×10^{3}	0.999
SJRA No. 3	8.460	6.615×10^{-4}	8.459	$(\mathbf{8.400}, \mathbf{8.518})$	2.035×10^{3}	1.000
Sacramento	8.984	7.364×10^{-5}	8.984	$(\mathbf{8.977}, \mathbf{8.991})$	2.678×10^{3}	1.000
Salina	9.084	3.385×10^{-4}	9.083	$(\mathbf{9.055}, \mathbf{9.114})$	1.915×10^{3}	1.001
San Francisco	8.892	1.345×10^{-4}	8.893	$(\mathbf{8.879}, \mathbf{8.905})$	2.524×10^{3}	1.000
San Jose-Santa Clara	9.165	1.133×10^{-4}	9.165	$(\mathbf{9.157}, \mathbf{9.174})$	1.617×10^{3}	1.003
San Leandro	8.796	5.694×10^{-4}	8.796	$(\mathbf{8.746}, \mathbf{8.846})$	2.078×10^{3}	1.000
San Mateo & Estero	8.876	3.694×10^{-4}	8.876	$(\mathbf{8.842}, \mathbf{8.908})$	1.988×10^{3}	1.002
Santa Cruz (City)	8.700	3.513×10^{-4}	8.700	$(\mathbf{8.669}, \mathbf{8.731})$	2.155×10^{3}	1.001
Santa Cruz (County)	8.700	3.760×10^{-4}	8.701	$(\mathbf{8.672}, \mathbf{8.728})$	1.506×10^{3}	1.002
Santa Rosa	9.149	2.336×10^{-4}	9.149	$(\mathbf{9.128}, \mathbf{9.170})$	2.204×10^{3}	1.001
Sausalito-Marin	8.547	4.356×10^{-4}	8.546	$(\mathbf{8.506}, \mathbf{8.586})$	2.183×10^{3}	1.000
Seaford	8.584	3.530×10^{-4}	8.584	$(\mathbf{8.552}, \mathbf{8.617})$	2.219×10^{3}	1.001
Silicon Valley	8.982	1.642×10^{-4}	8.982	$(\mathbf{8.968}, \mathbf{8.996})$	1.944×10^{3}	1.001
Somerset Raritan	8.388	1.206×10^{-3}	8.388	$(\mathbf{8.283}, \mathbf{8.485})$	1.865×10^{3}	1.002
Soscol	9.267	2.991×10^{-4}	9.267	$(\mathbf{9.244}, \mathbf{9.293})$	1.747×10^{3}	1.001
South Bay	9.641	5.511×10^{-4}	9.642	$(\mathbf{9.587}, \mathbf{9.692})$	2.188×10^{3}	1.001
South Bend	8.640	4.366×10^{-4}	8.640	$(\mathbf{8.598}, \mathbf{8.680})$	2.295×10^{3}	1.003
South Burlington	8.477	4.175×10^{-4}	8.476	$(\mathbf{8.440}, \mathbf{8.514})$	1.978×10^{3}	1.002
South Columbus	8.487	3.092×10^{-4}	8.487	$(\mathbf{8.459}, \mathbf{8.515})$	2.192×10^{3}	1.002
South County	8.975	1.822×10^{-4}	8.975	$(\mathbf{8.959}, \mathbf{8.992})$	2.104×10^{3}	0.999
South Laredo	9.370	4.787×10^{-4}	9.371	$(\mathbf{9.318}, \mathbf{9.421})$	2.965×10^{3}	1.001
South Monmouth	8.451	3.601×10^{-4}	8.451	$(\mathbf{8.413}, \mathbf{8.490})$	2.987×10^{3}	0.999
South River	8.389	5.204×10^{-4}	8.388	$(\mathbf{8.349}, \mathbf{8.429})$	1.613×10^{3}	1.004
South Water	8.383	3.679×10^{-4}	8.383	$(\mathbf{8.349}, \mathbf{8.416})$	2.046×10^{3}	1.001
Southern Marin	8.478	2.385×10^{-4}	8.478	$(\mathbf{8.454}, \mathbf{8.503})$	2.712×10^{3}	1.000
St. Cloud	8.487	5.908×10^{-4}	8.486	$(\mathbf{8.434}, \mathbf{8.540})$	2.148×10^{3}	1.000
Sunnyvale	9.285	1.068×10^{-4}	9.285	$(\mathbf{9.275}, \mathbf{9.295})$	2.164×10^{3}	1.000
Traverse City	8.430	5.461×10^{-4}	8.430	$(\mathbf{8.380}, \mathbf{8.478})$	2.196×10^{3}	1.000
Turkey Creek	8.397	4.811×10^{-4}	8.398	$(\mathbf{8.355}, \mathbf{8.437})$	1.878×10^{3}	1.001
Turlock	8.639	4.872×10^{-4}	8.638	$(\mathbf{8.587}, \mathbf{8.691})$	2.929×10^{3}	1.000
Upper Blackstone	8.246	7.116×10^{-4}	8.246	$(\mathbf{8.186}, \mathbf{8.305})$	1.778×10^{3}	1.001
Utoy Creek	8.298	3.969×10^{-4}	8.298	$(\mathbf{8.263}, \mathbf{8.332})$	1.947×10^{3}	1.001
Vallejo	8.940	4.577×10^{-4}	8.940	$(\mathbf{8.898}, \mathbf{8.983})$	2.204×10^{3}	1.000
Valley	9.313	2.790×10^{-4}	9.313	$(\mathbf{9.287}, \mathbf{9.339})$	2.310×10^{3}	1.000
Valley Creek	8.523	3.862×10^{-4}	8.523	$(\mathbf{8.490}, \mathbf{8.557})$	2.056×10^{3}	1.000
Village Creek	8.475	4.195×10^{-4}	8.476	$(\mathbf{8.438}, \mathbf{8.511})$	2.013×10^{3}	1.001
Warren	8.418	3.827×10^{-4}	8.418	$(\mathbf{8.382}, \mathbf{8.455})$	2.305×10^{3}	1.001
Weaton	8.749	2.468×10^{-4}	8.749	$(\mathbf{8.726}, \mathbf{8.772})$	2.214×10^{3}	0.999
West Boise	8.471	5.419×10^{-4}	8.471	$(\mathbf{8.423}, \mathbf{8.518})$	2.045×10^{3}	1.001
West County	8.814	4.063×10^{-4}	8.814	$(\mathbf{8.776}, \mathbf{8.852})$	2.336×10^{3}	1.000

Table S5: Mean, Monte Carlo standard error, median, 95% credible interval, effective sample size, and $\hat{R}$ for intercept calculated over 4,000 posterior samples. Bolded intervals do not contain 0.

Site	Mean	MCSE	Median	95% CI	ESS	$\hat{R}$
Wheeling	8.375	3.610×10^{-4}	8.375	$(\mathbf{8.345}, \mathbf{8.405})$	1.769×10^{3}	1.003
Wichita Falls	8.898	3.671×10^{-4}	8.898	$(\mathbf{8.870}, \mathbf{8.928})$	1.609×10^{3}	1.000
Windsor	8.874	5.929×10^{-4}	8.873	$(\mathbf{8.814}, \mathbf{8.934})$	2.614×10^{3}	1.001
Winters	8.761	5.543×10^{-4}	8.760	$(\mathbf{8.712}, \mathbf{8.812})$	2.139×10^{3}	1.001
Wolcott	8.598	5.376×10^{-4}	8.598	$(\mathbf{8.546}, \mathbf{8.654})$	2.593×10^{3}	1.001
Woodland	8.999	4.946×10^{-4}	8.998	$(\mathbf{8.953}, \mathbf{9.046})$	2.305×10^{3}	1.000
Yankton	8.401	5.361×10^{-4}	8.401	$(\mathbf{8.354}, \mathbf{8.449})$	2.004×10^{3}	1.000
York	8.051	4.325×10^{-4}	8.051	$(\mathbf{8.011}, \mathbf{8.089})$	2.155×10^{3}	1.001
Youngstown	8.368	4.360×10^{-4}	8.368	$(\mathbf{8.329}, \mathbf{8.405})$	1.974×10^{3}	1.002
Zacate Creek	9.307	5.639×10^{-4}	9.307	$(\mathbf{9.256}, \mathbf{9.359})$	2.178×10^{3}	1.001

Table S6: Mean, Monte Carlo standard error, median, 95% credible interval, effective sample size, and $\hat{R}$ for coefficient on precipitation calculated over 4,000 posterior samples. Bolded intervals do not contain 0.

Site	Mean	MCSE	Median	95% CI	ESS	$\hat{R}$
Akron	-1.809×10^{-2}	3.310×10^{-4}	-1.606×10^{-2}	$(-6.035 \times 10^{-2}, 1.032 \times 10^{-2})$	2.913×10^{3}	1.000
Altamonte Springs	1.808×10^{-2}	3.978×10^{-4}	1.840×10^{-2}	$(-2.094 \times 10^{-2}, 5.497 \times 10^{-2})$	2.270×10^{3}	1.000
Ann Arbor	1.240×10^{-2}	3.105×10^{-4}	1.183×10^{-2}	$(-1.707 \times 10^{-2}, 4.394 \times 10^{-2})$	2.459×10^{3}	1.000
Aquia	-6.988×10^{-3}	2.434×10^{-4}	-6.167×10^{-3}	$(-3.049 \times 10^{-2}, 1.498 \times 10^{-2})$	2.080×10^{3}	1.000
Archie Elledge	-2.059×10^{-2}	3.537×10^{-4}	-2.030×10^{-2}	$(-5.469 \times 10^{-2}, 1.106 \times 10^{-2})$	2.312×10^{3}	1.000
Bangor	7.064×10^{-3}	1.313×10^{-3}	6.212×10^{-3}	$(-0.144, 0.145)$	2.834×10^{3}	1.002
Bayshore	-7.131×10^{-3}	6.881×10^{-4}	-4.842×10^{-3}	$(-8.109 \times 10^{-2}, 6.818 \times 10^{-2})$	2.913×10^{3}	1.001
Big Creek	3.329×10^{-4}	2.812×10^{-4}	8.591×10^{-4}	$(-3.681 \times 10^{-2}, 3.095 \times 10^{-2})$	3.227×10^{3}	1.000
Boege Alvarado (Fremont)	-8.852×10^{-3}	4.165×10^{-4}	-8.352×10^{-3}	$(-5.287 \times 10^{-2}, 3.842 \times 10^{-2})$	2.830×10^{3}	1.001
Boege Alvarado (Newark)	-2.019×10^{-2}	7.421×10^{-4}	-1.416×10^{-2}	$(-0.101, 4.589 \times 10^{-2})$	2.629×10^{3}	1.002
Boege Alvarado (Union City)	3.157×10^{-2}	7.845×10^{-4}	3.091×10^{-2}	$(-3.588 \times 10^{-2}, 0.101)$	2.231×10^{3}	1.000
Brunswick	4.467×10^{-2}	4.099×10^{-4}	4.444×10^{-2}	$(\mathbf{6.986 \times 10^{-3}}, \mathbf{8.136 \times 10^{-2}})$	2.296×10^{3}	1.000
CODIGA	9.233×10^{-3}	2.421×10^{-4}	8.287×10^{-3}	$(-1.554 \times 10^{-2}, 3.724 \times 10^{-2})$	2.932×10^{3}	1.000
Cahaba River	-1.401×10^{-3}	2.145×10^{-4}	-9.767×10^{-4}	$(-2.656 \times 10^{-2}, 2.575 \times 10^{-2})$	3.403×10^{3}	1.001
Calera Creek	-1.461×10^{-2}	4.164×10^{-4}	-1.121×10^{-2}	$(-5.965 \times 10^{-2}, 2.086 \times 10^{-2})$	2.632×10^{3}	1.000
Camp Creek	-8.511×10^{-3}	3.496×10^{-4}	-7.076×10^{-3}	$(-4.422 \times 10^{-2}, 2.418 \times 10^{-2})$	2.470×10^{3}	1.000
Capital Region	2.649×10^{-3}	2.970×10^{-4}	2.896×10^{-3}	$(-2.953 \times 10^{-2}, 3.068 \times 10^{-2})$	2.678×10^{3}	1.000
Carmel	-9.424×10^{-4}	3.537×10^{-4}	-6.979×10^{-4}	$(-3.877 \times 10^{-2}, 3.923 \times 10^{-2})$	2.790×10^{3}	1.002
Central Contra Costa	-2.399×10^{-2}	2.294×10^{-4}	-2.352×10^{-2}	$(-4.891 \times 10^{-2}, 3.605 \times 10^{-6})$	2.726×10^{3}	1.000
Central Marin	-8.674×10^{-2}	6.889×10^{-4}	-8.829×10^{-2}	$(\mathbf{-0.159}, \mathbf{-1.032 \times 10^{-2}})$	3.121×10^{3}	0.999
Central Marin (W Railroad)	-8.188×10^{-2}	7.488×10^{-4}	-8.247×10^{-2}	$(\mathbf{-0.140}, \mathbf{-1.835 \times 10^{-2}})$	1.544×10^{3}	1.001
Central Valley	-1.209×10^{-2}	2.603×10^{-4}	-1.211×10^{-2}	$(-3.298 \times 10^{-2}, 8.226 \times 10^{-3})$	1.699×10^{3}	1.001
Clark County	-3.093×10^{-2}	6.192×10^{-4}	-3.256×10^{-2}	$(-8.295 \times 10^{-2}, 2.434 \times 10^{-2})$	2.056×10^{3}	1.001
Clinton	-9.949×10^{-3}	1.900×10^{-4}	-9.120×10^{-3}	$(-2.987 \times 10^{-2}, 6.029 \times 10^{-3})$	2.395×10^{3}	1.000
Coastal	1.773×10^{-2}	3.913×10^{-4}	1.643×10^{-2}	$(-1.603 \times 10^{-2}, 6.215 \times 10^{-2})$	2.705×10^{3}	0.999
Coeur d'Alene	-6.649×10^{-3}	2.118×10^{-4}	-5.963×10^{-3}	$(-3.009 \times 10^{-2}, 1.396 \times 10^{-2})$	2.665×10^{3}	1.000
Coralville	-1.229×10^{-2}	3.880×10^{-4}	-9.989×10^{-3}	$(-5.991 \times 10^{-2}, 2.570 \times 10^{-2})$	2.926×10^{3}	1.000
Cumberland	2.632×10^{-2}	6.138×10^{-4}	2.857×10^{-2}	$(-3.735 \times 10^{-2}, 8.190 \times 10^{-2})$	2.377×10^{3}	0.999
DELCORA	-5.644×10^{-2}	3.943×10^{-4}	-5.687×10^{-2}	$(\mathbf{-9.809 \times 10^{-2}}, \mathbf{-1.207 \times 10^{-2}})$	2.751×10^{3}	1.001
Davis	-3.595×10^{-3}	1.324×10^{-4}	-2.408×10^{-3}	$(-1.945 \times 10^{-2}, 7.765 \times 10^{-3})$	2.676×10^{3}	1.002
Deer Island	-9.915×10^{-3}	2.956×10^{-4}	-8.505×10^{-3}	$(-4.602 \times 10^{-2}, 2.252 \times 10^{-2})$	3.274×10^{3}	0.999
Dillman Road	-1.776×10^{-2}	3.964×10^{-4}	-1.605×10^{-2}	$(-5.981 \times 10^{-2}, 1.737 \times 10^{-2})$	2.566×10^{3}	1.000
Dover	-1.616×10^{-3}	3.044×10^{-4}	-1.961×10^{-3}	$(-3.813 \times 10^{-2}, 3.668 \times 10^{-2})$	3.614×10^{3}	1.000

Table S6: Mean, Monte Carlo standard error, median, 95% credible interval, effective sample size, and $\hat{R}$ for coefficient on precipitation calculated over 4,000 posterior samples. Bolded intervals do not contain 0.

Site	Mean	MCSE	Median	95% CI	ESS	$\hat{R}$
Duck Creek	3.677×10^{-3}	2.336×10^{-4}	2.616×10^{-3}	$(-1.950 \times 10^{-2}, 2.760 \times 10^{-2})$	2.532×10^{3}	1.002
E.W. Blom Point Loma	-2.431×10^{-2}	3.631×10^{-4}	-2.384×10^{-2}	$(-5.831 \times 10^{-2}, 6.104 \times 10^{-3})$	2.072×10^{3}	1.000
East Bay	8.823×10^{-4}	2.393×10^{-4}	3.950×10^{-4}	$(-2.476 \times 10^{-2}, 2.739 \times 10^{-2})$	2.765×10^{3}	1.000
Eastern	2.874×10^{-3}	2.252×10^{-4}	2.122×10^{-3}	$(-2.137 \times 10^{-2}, 2.971 \times 10^{-2})$	2.912×10^{3}	1.001
Ellis Creek	-1.731×10^{-3}	3.383×10^{-4}	-1.844×10^{-3}	$(-3.636 \times 10^{-2}, 3.385 \times 10^{-2})$	2.770×10^{3}	1.000
Esparto	-2.785×10^{-2}	5.918×10^{-4}	-2.783×10^{-2}	$(-8.053 \times 10^{-2}, 2.620 \times 10^{-2})$	2.120×10^{3}	1.000
Essex	-5.453×10^{-3}	3.254×10^{-4}	-3.683×10^{-3}	$(-4.790 \times 10^{-2}, 2.797 \times 10^{-2})$	3.169×10^{3}	1.000
Fairfield-Suisun	-2.026×10^{-2}	2.903×10^{-4}	-1.984×10^{-2}	$(-4.828 \times 10^{-2}, 3.728 \times 10^{-3})$	2.132×10^{3}	1.002
Five Mile Creek	3.990×10^{-3}	5.177×10^{-4}	5.407×10^{-3}	$(-4.836 \times 10^{-2}, 4.173 \times 10^{-2})$	2.026×10^{3}	1.000
Gainesville	-4.775×10^{-2}	4.202×10^{-4}	-4.926×10^{-2}	$\mathbf{(-8.430 \times 10^{-2}, -8.336 \times 10^{-4})}$	2.315×10^{3}	1.003
Garland Rowlett Creek	-1.578×10^{-2}	2.257×10^{-4}	-1.590×10^{-2}	$(-4.010 \times 10^{-2}, 5.669 \times 10^{-3})$	2.576×10^{3}	1.002
Glenbard	-1.786×10^{-2}	2.155×10^{-4}	-1.777×10^{-2}	$(-4.200 \times 10^{-2}, 5.209 \times 10^{-3})$	3.047×10^{3}	1.000
Grandville	2.568×10^{-2}	3.956×10^{-4}	2.552×10^{-2}	$(-5.676 \times 10^{-3}, 5.887 \times 10^{-2})$	1.888×10^{3}	1.004
Hagerstown	1.948×10^{-2}	6.653×10^{-4}	1.696×10^{-2}	$(-3.152 \times 10^{-2}, 7.721 \times 10^{-2})$	1.753×10^{3}	1.001
Hall Street	-1.635×10^{-2}	3.564×10^{-4}	-1.639×10^{-2}	$(-4.571 \times 10^{-2}, 1.314 \times 10^{-2})$	1.920×10^{3}	1.002
Hamlin	3.814×10^{-2}	8.360×10^{-4}	4.165×10^{-2}	$(-2.387 \times 10^{-2}, 8.890 \times 10^{-2})$	1.333×10^{3}	1.002
Harrison	3.152×10^{-2}	6.860×10^{-4}	3.094×10^{-2}	$(-3.132 \times 10^{-2}, 9.598 \times 10^{-2})$	2.238×10^{3}	1.001
Hillsville	7.801×10^{-3}	2.327×10^{-4}	8.091×10^{-3}	$(-1.192 \times 10^{-2}, 2.498 \times 10^{-2})$	1.648×10^{3}	1.000
Hollister	-1.330×10^{-2}	4.374×10^{-4}	-1.228×10^{-2}	$(-5.512 \times 10^{-2}, 3.000 \times 10^{-2})$	2.414×10^{3}	1.000
Hollywood Road	1.261×10^{-2}	5.442×10^{-4}	7.763×10^{-3}	$(-4.090 \times 10^{-2}, 8.605 \times 10^{-2})$	3.115×10^{3}	1.001
Hyperion	-1.364×10^{-2}	3.837×10^{-4}	-1.147×10^{-2}	$(-5.055 \times 10^{-2}, 1.894 \times 10^{-2})$	2.254×10^{3}	1.001
JB Latham	-6.141×10^{-3}	3.624×10^{-4}	-6.580×10^{-3}	$(-4.617 \times 10^{-2}, 4.244 \times 10^{-2})$	3.422×10^{3}	1.000
Jackson	-7.523×10^{-3}	1.868×10^{-4}	-8.142×10^{-3}	$(-2.323 \times 10^{-2}, 1.070 \times 10^{-2})$	2.109×10^{3}	1.000
Jeffersonville	-2.125×10^{-2}	4.112×10^{-4}	-2.151×10^{-2}	$(-5.662 \times 10^{-2}, 1.429 \times 10^{-2})$	1.994×10^{3}	1.003
John M. Asplund	-1.940×10^{-2}	7.819×10^{-4}	-1.647×10^{-2}	$(-0.119, 7.678 \times 10^{-2})$	3.580×10^{3}	1.000
Johnnie Mosley	2.506×10^{-2}	4.988×10^{-4}	2.254×10^{-2}	$(-1.695 \times 10^{-2}, 8.186 \times 10^{-2})$	2.583×10^{3}	1.001
Johns Creek	-1.386×10^{-2}	5.561×10^{-4}	-1.476×10^{-2}	$(-5.146 \times 10^{-2}, 3.277 \times 10^{-2})$	1.562×10^{3}	1.002
Joint	-1.619×10^{-3}	1.776×10^{-4}	-1.539×10^{-3}	$(-2.046 \times 10^{-2}, 1.976 \times 10^{-2})$	3.196×10^{3}	0.999
Kansas City	-7.011×10^{-3}	4.613×10^{-4}	-3.982×10^{-3}	$(-5.668 \times 10^{-2}, 3.445 \times 10^{-2})$	2.590×10^{3}	1.000
Kaw Point	-1.271×10^{-2}	4.878×10^{-4}	-7.911×10^{-3}	$(-7.896 \times 10^{-2}, 3.116 \times 10^{-2})$	3.121×10^{3}	1.000
Lancaster	-2.770×10^{-3}	2.797×10^{-4}	-1.499×10^{-3}	$(-3.817 \times 10^{-2}, 2.910 \times 10^{-2})$	3.445×10^{3}	1.000
Lander Street	-4.173×10^{-3}	3.621×10^{-4}	-3.899×10^{-3}	$(-4.441 \times 10^{-2}, 3.965 \times 10^{-2})$	3.218×10^{3}	0.999
Las Gallinas	3.935×10^{-3}	3.186×10^{-4}	3.488×10^{-3}	$(-3.088 \times 10^{-2}, 3.969 \times 10^{-2})$	2.931×10^{3}	1.000
Lawrence Kansas	8.447×10^{-3}	4.952×10^{-4}	5.344×10^{-3}	$(-3.195 \times 10^{-2}, 6.168 \times 10^{-2})$	2.397×10^{3}	1.000
Little Falls Run	5.143×10^{-4}	1.940×10^{-4}	5.118×10^{-4}	$(-2.161 \times 10^{-2}, 2.227 \times 10^{-2})$	3.013×10^{3}	1.000
Little River	-1.356×10^{-2}	2.884×10^{-4}	-1.185×10^{-2}	$(-4.799 \times 10^{-2}, 1.492 \times 10^{-2})$	2.999×10^{3}	1.001
Lompoc	-1.691×10^{-3}	4.453×10^{-4}	-1.153×10^{-3}	$(-5.586 \times 10^{-2}, 4.939 \times 10^{-2})$	3.212×10^{3}	0.999
Los Banos	4.481×10^{-4}	4.089×10^{-4}	-4.243×10^{-4}	$(-4.307 \times 10^{-2}, 4.658 \times 10^{-2})$	2.941×10^{3}	0.999
Loxahatchee	3.094×10^{-2}	3.429×10^{-4}	3.224×10^{-2}	$(-3.540 \times 10^{-3}, 6.082 \times 10^{-2})$	2.257×10^{3}	1.000
MDWASD Central	-5.023×10^{-3}	3.222×10^{-4}	-3.837×10^{-3}	$(-4.309 \times 10^{-2}, 3.104 \times 10^{-2})$	3.085×10^{3}	1.000
MDWASD North	-3.918×10^{-2}	5.957×10^{-4}	-3.673×10^{-2}	$(-0.104, 1.248 \times 10^{-2})$	2.617×10^{3}	0.999
MDWASD South	-2.141×10^{-2}	5.024×10^{-4}	-2.247×10^{-2}	$(-6.218 \times 10^{-2}, 2.444 \times 10^{-2})$	1.988×10^{3}	1.001
Madera	-1.334×10^{-2}	4.271×10^{-4}	-1.331×10^{-2}	$(-5.287 \times 10^{-2}, 2.761 \times 10^{-2})$	2.127×10^{3}	1.001
Mankato	-4.924×10^{-3}	1.562×10^{-4}	-4.268×10^{-3}	$(-2.216 \times 10^{-2}, 1.052 \times 10^{-2})$	2.843×10^{3}	0.999
Markshalltown	3.495×10^{-2}	6.904×10^{-4}	3.340×10^{-2}	$(-2.477 \times 10^{-2}, 0.104)$	2.367×10^{3}	1.001
Marlay Taylor	-5.086×10^{-3}	4.203×10^{-4}	-4.922×10^{-3}	$(-4.752 \times 10^{-2}, 3.876 \times 10^{-2})$	2.601×10^{3}	1.000
Merced	-1.309×10^{-2}	2.799×10^{-4}	-1.370×10^{-2}	$(-3.615 \times 10^{-2}, 1.236 \times 10^{-2})$	2.203×10^{3}	1.000
Mid-Coastside	-1.470×10^{-2}	3.501×10^{-4}	-1.449×10^{-2}	$(-4.673 \times 10^{-2}, 1.735 \times 10^{-2})$	2.118×10^{3}	1.000
Modesto's Sutter	-2.391×10^{-2}	2.173×10^{-4}	-2.379×10^{-2}	$\mathbf{(-4.439 \times 10^{-2}, -2.716 \times 10^{-3})}$	2.375×10^{3}	1.000
Montpelier	-7.273×10^{-2}	6.603×10^{-4}	-7.341×10^{-2}	$\mathbf{(-0.138, -4.511 \times 10^{-3})}$	2.780×10^{3}	1.001

Table S6: Mean, Monte Carlo standard error, median, 95% credible interval, effective sample size, and $\hat{R}$ for coefficient on precipitation calculated over 4,000 posterior samples. Bolded intervals do not contain 0.

Site	Mean	MCSE	Median	95% CI	ESS	$\hat{R}$
Monterey One	-8.727×10^{-4}	6.993×10^{-4}	-6.383×10^{-4}	$(-7.511 \times 10^{-2}, 7.129 \times 10^{-2})$	2.606×10^{3}	1.001
Morris Forman	5.715×10^{-3}	4.206×10^{-4}	7.093×10^{-3}	$(-4.445 \times 10^{-2}, 4.297 \times 10^{-2})$	2.635×10^{3}	1.002
Mt. Pleasant	-5.254×10^{-3}	5.424×10^{-4}	-2.925×10^{-3}	$(-7.226 \times 10^{-2}, 5.438 \times 10^{-2})$	3.282×10^{3}	1.001
Muscatine	-3.124×10^{-2}	4.083×10^{-4}	-3.133×10^{-2}	$(-7.290 \times 10^{-2}, 6.365 \times 10^{-3})$	2.550×10^{3}	1.000
Norhtwest Water	2.141×10^{-3}	2.585×10^{-4}	2.931×10^{-3}	$(-2.890 \times 10^{-2}, 2.652 \times 10^{-2})$	2.842×10^{3}	1.001
North Water	1.187×10^{-2}	2.898×10^{-4}	1.062×10^{-2}	$(-1.834 \times 10^{-2}, 4.469 \times 10^{-2})$	3.065×10^{3}	1.001
Novato	-1.750×10^{-3}	2.471×10^{-4}	-1.140×10^{-3}	$(-3.050 \times 10^{-2}, 2.458 \times 10^{-2})$	3.020×10^{3}	1.002
Ocean	9.866×10^{-3}	7.034×10^{-4}	7.718×10^{-3}	$(-6.852 \times 10^{-2}, 9.625 \times 10^{-2})$	3.289×10^{3}	1.001
Oceanside	-4.379×10^{-2}	1.385×10^{-4}	-4.352×10^{-2}	$\mathbf{(-6.170 \times 10^{-2}, -2.660 \times 10^{-2})}$	3.957×10^{3}	1.001
Ottumwa	-8.094×10^{-2}	2.795×10^{-4}	-8.152×10^{-2}	$\mathbf{(-0.113, -4.599 \times 10^{-2})}$	3.756×10^{3}	1.001
Palo Alto	-1.337×10^{-2}	7.659×10^{-5}	-1.324×10^{-2}	$\mathbf{(-2.204 \times 10^{-2}, -4.381 \times 10^{-3})}$	3.446×10^{3}	1.000
Parker North	2.530×10^{-2}	3.884×10^{-4}	2.624×10^{-2}	$(-1.259 \times 10^{-2}, 6.024 \times 10^{-2})$	2.166×10^{3}	1.001
Parker South	-1.008×10^{-2}	5.010×10^{-4}	-9.510×10^{-3}	$(-5.620 \times 10^{-2}, 4.113 \times 10^{-2})$	2.459×10^{3}	1.001
Paso Robles	-1.642×10^{-2}	3.659×10^{-4}	-1.692×10^{-2}	$(-4.233 \times 10^{-2}, 8.272 \times 10^{-3})$	1.441×10^{3}	1.004
Passaic Valley	-2.418×10^{-2}	4.199×10^{-4}	-2.330×10^{-2}	$(-6.384 \times 10^{-2}, 8.937 \times 10^{-3})$	2.133×10^{3}	0.999
Penacook	-3.254×10^{-2}	4.037×10^{-4}	-3.356×10^{-2}	$(-6.497 \times 10^{-2}, 3.057 \times 10^{-3})$	1.942×10^{3}	1.000
Portland	-3.512×10^{-2}	3.345×10^{-4}	-3.444×10^{-2}	$(-7.325 \times 10^{-2}, 5.016 \times 10^{-5})$	3.000×10^{3}	1.000
Provo City	-2.422×10^{-2}	4.348×10^{-4}	-2.425×10^{-2}	$(-6.059 \times 10^{-2}, 9.012 \times 10^{-3})$	1.840×10^{3}	1.002
RM Clayton	-4.994×10^{-2}	3.493×10^{-4}	-5.018×10^{-2}	$\mathbf{(-8.717 \times 10^{-2}, -1.259 \times 10^{-2})}$	2.781×10^{3}	1.001
Red Wing	-3.885×10^{-3}	3.991×10^{-4}	-2.079×10^{-3}	$(-5.420 \times 10^{-2}, 3.753 \times 10^{-2})$	2.874×10^{3}	1.000
Regional	1.900×10^{-3}	3.029×10^{-4}	-1.818×10^{-4}	$(-3.072 \times 10^{-2}, 4.525 \times 10^{-2})$	3.538×10^{3}	1.000
Regional No. 1	2.115×10^{-3}	1.972×10^{-4}	1.927×10^{-3}	$(-1.920 \times 10^{-2}, 2.146 \times 10^{-2})$	2.623×10^{3}	1.002
River Road	-1.196×10^{-2}	4.688×10^{-4}	-1.257×10^{-2}	$(-5.395 \times 10^{-2}, 3.829 \times 10^{-2})$	2.273×10^{3}	1.002
Riverside	2.020×10^{-3}	5.082×10^{-4}	1.510×10^{-3}	$(-5.330 \times 10^{-2}, 6.138 \times 10^{-2})$	3.234×10^{3}	1.002
Rochester	-8.291×10^{-3}	2.634×10^{-4}	-7.564×10^{-3}	$(-3.394 \times 10^{-2}, 1.559 \times 10^{-2})$	2.294×10^{3}	1.003
SJRA No. 1	-2.548×10^{-2}	4.208×10^{-4}	-2.591×10^{-2}	$(-6.657 \times 10^{-2}, 1.332 \times 10^{-2})$	2.429×10^{3}	1.000
SJRA No. 2	-3.814×10^{-2}	4.585×10^{-4}	-3.953×10^{-2}	$(-7.857 \times 10^{-2}, 5.759 \times 10^{-3})$	2.257×10^{3}	1.002
SJRA No. 3	-1.144×10^{-2}	5.260×10^{-4}	-8.234×10^{-3}	$(-6.987 \times 10^{-2}, 3.952 \times 10^{-2})$	2.845×10^{3}	1.000
Sacramento	-1.534×10^{-2}	8.516×10^{-5}	-1.512×10^{-2}	$\mathbf{(-2.575 \times 10^{-2}, -5.082 \times 10^{-3})}$	3.737×10^{3}	1.000
Salina	-5.759×10^{-3}	3.799×10^{-4}	-3.916×10^{-3}	$(-4.487 \times 10^{-2}, 2.898 \times 10^{-2})$	2.644×10^{3}	1.002
San Francisco	-6.360×10^{-3}	1.514×10^{-4}	-5.654×10^{-3}	$(-2.374 \times 10^{-2}, 7.345 \times 10^{-3})$	2.870×10^{3}	1.000
San Jose-Santa Clara	-5.540×10^{-3}	8.538×10^{-5}	-5.605×10^{-3}	$(-1.336 \times 10^{-2}, 2.343 \times 10^{-3})$	2.415×10^{3}	1.001
San Leandro	-4.067×10^{-2}	4.713×10^{-4}	-3.981×10^{-2}	$(-8.715 \times 10^{-2}, 3.281 \times 10^{-3})$	2.395×10^{3}	1.002
San Mateo & Estero	-6.719×10^{-3}	3.886×10^{-4}	-6.672×10^{-3}	$(-3.692 \times 10^{-2}, 2.554 \times 10^{-2})$	1.597×10^{3}	1.002
Santa Cruz (City)	-1.849×10^{-2}	4.030×10^{-4}	-1.734×10^{-2}	$(-5.389 \times 10^{-2}, 1.110 \times 10^{-2})$	1.900×10^{3}	1.002
Santa Cruz (County)	-1.274×10^{-2}	2.662×10^{-4}	-1.245×10^{-2}	$(-3.613 \times 10^{-2}, 9.318 \times 10^{-3})$	1.951×10^{3}	1.000
Santa Rosa	-3.287×10^{-3}	1.933×10^{-4}	-3.129×10^{-3}	$(-2.266 \times 10^{-2}, 1.801 \times 10^{-2})$	2.781×10^{3}	1.000
Sausalito-Marin	-3.473×10^{-2}	4.892×10^{-4}	-3.348×10^{-2}	$(-8.178 \times 10^{-2}, 7.410 \times 10^{-3})$	2.384×10^{3}	1.002
Seaford	-4.541×10^{-3}	2.763×10^{-4}	-3.534×10^{-3}	$(-3.692 \times 10^{-2}, 2.430 \times 10^{-2})$	2.906×10^{3}	1.000
Silicon Valley	-2.421×10^{-2}	1.214×10^{-4}	-2.405×10^{-2}	$\mathbf{(-3.769 \times 10^{-2}, -1.109 \times 10^{-2})}$	3.051×10^{3}	1.000
Somerset Raritan	-7.843×10^{-2}	1.041×10^{-3}	-7.130×10^{-2}	$(-0.207, 1.435 \times 10^{-2})$	2.975×10^{3}	1.000
Soscol	-1.485×10^{-2}	2.361×10^{-4}	-1.568×10^{-2}	$(-3.572 \times 10^{-2}, 7.988 \times 10^{-3})$	2.347×10^{3}	1.001
South Bay	7.779×10^{-3}	5.481×10^{-4}	8.704×10^{-3}	$(-4.819 \times 10^{-2}, 5.297 \times 10^{-2})$	2.096×10^{3}	1.000
South Bend	-1.336×10^{-2}	4.556×10^{-4}	-1.313×10^{-2}	$(-5.749 \times 10^{-2}, 2.919 \times 10^{-2})$	2.317×10^{3}	1.000
South Burlington	-1.306×10^{-2}	4.678×10^{-4}	-1.103×10^{-2}	$(-6.072 \times 10^{-2}, 3.404 \times 10^{-2})$	2.551×10^{3}	1.000
South Columbus	-5.981×10^{-3}	2.143×10^{-4}	-4.664×10^{-3}	$(-3.143 \times 10^{-2}, 1.713 \times 10^{-2})$	3.224×10^{3}	1.000
South County	-1.090×10^{-2}	1.918×10^{-4}	-1.093×10^{-2}	$(-2.858 \times 10^{-2}, 7.317 \times 10^{-3})$	2.295×10^{3}	1.000
South Laredo	2.675×10^{-2}	5.788×10^{-4}	2.892×10^{-2}	$(-3.727 \times 10^{-2}, 8.272 \times 10^{-2})$	2.543×10^{3}	1.000
South Monmouth	-8.227×10^{-3}	4.340×10^{-4}	-8.595×10^{-3}	$(-4.698 \times 10^{-2}, 3.925 \times 10^{-2})$	2.439×10^{3}	1.001
South River	5.010×10^{-3}	3.047×10^{-4}	4.374×10^{-3}	$(-2.910 \times 10^{-2}, 3.919 \times 10^{-2})$	3.038×10^{3}	1.000

Table S6: Mean, Monte Carlo standard error, median, 95% credible interval, effective sample size, and $\hat{R}$ for coefficient on precipitation calculated over 4,000 posterior samples. Bolded intervals do not contain 0.

Site	Mean	MCSE	Median	95% CI	ESS	$\hat{R}$
South Water	-1.084×10^{-2}	3.244×10^{-4}	-1.159×10^{-2}	$(-4.037 \times 10^{-2}, 2.420 \times 10^{-2})$	2.440×10^{3}	1.001
Southern Marin	-5.214×10^{-2}	1.861×10^{-4}	-5.246×10^{-2}	$(\mathbf{-7.546 \times 10^{-2}, -2.855 \times 10^{-2}})$	4.061×10^{3}	1.000
St. Cloud	1.034×10^{-2}	5.309×10^{-4}	8.011×10^{-3}	$(-3.684 \times 10^{-2}, 6.670 \times 10^{-2})$	2.237×10^{3}	1.002
Sunnyvale	-1.351×10^{-2}	9.469×10^{-5}	-1.361×10^{-2}	$(\mathbf{-2.278 \times 10^{-2}, -4.186 \times 10^{-3}})$	2.372×10^{3}	1.001
Traverse City	1.327×10^{-2}	4.055×10^{-4}	1.362×10^{-2}	$(-2.662 \times 10^{-2}, 5.029 \times 10^{-2})$	2.225×10^{3}	1.000
Turkey Creek	-2.365×10^{-3}	3.155×10^{-4}	-1.939×10^{-3}	$(-4.175 \times 10^{-2}, 3.861 \times 10^{-2})$	3.776×10^{3}	0.999
Turlock	-1.759×10^{-2}	5.604×10^{-4}	-1.323×10^{-2}	$(-8.806 \times 10^{-2}, 3.453 \times 10^{-2})$	2.973×10^{3}	1.000
Upper Blackstone	-2.768×10^{-2}	5.261×10^{-4}	-2.666×10^{-2}	$(-8.031 \times 10^{-2}, 1.838 \times 10^{-2})$	2.431×10^{3}	1.000
Utoy Creek	-9.704×10^{-3}	2.658×10^{-4}	-9.056×10^{-3}	$(-3.993 \times 10^{-2}, 1.790 \times 10^{-2})$	3.072×10^{3}	1.000
Vallejo	-1.029×10^{-2}	4.017×10^{-4}	-8.775×10^{-3}	$(-5.372 \times 10^{-2}, 3.062 \times 10^{-2})$	2.803×10^{3}	1.000
Valley	-5.775×10^{-3}	3.558×10^{-4}	-5.875×10^{-3}	$(-3.917 \times 10^{-2}, 3.376 \times 10^{-2})$	2.604×10^{3}	1.001
Valley Creek	1.032×10^{-2}	3.356×10^{-4}	1.104×10^{-2}	$(-2.463 \times 10^{-2}, 3.989 \times 10^{-2})$	2.230×10^{3}	1.001
Village Creek	-7.821×10^{-3}	2.648×10^{-4}	-6.338×10^{-3}	$(-4.279 \times 10^{-2}, 2.237 \times 10^{-2})$	3.681×10^{3}	0.999
Warren	-1.245×10^{-2}	2.963×10^{-4}	-1.086×10^{-2}	$(-4.592 \times 10^{-2}, 1.564 \times 10^{-2})$	2.745×10^{3}	1.000
Weaton	-1.442×10^{-2}	3.081×10^{-4}	-1.163×10^{-2}	$(-4.886 \times 10^{-2}, 8.584 \times 10^{-3})$	2.372×10^{3}	1.000
West Boise	-1.797×10^{-2}	4.703×10^{-4}	-1.544×10^{-2}	$(-7.254 \times 10^{-2}, 2.381 \times 10^{-2})$	2.714×10^{3}	1.001
West County	1.079×10^{-2}	3.951×10^{-4}	8.081×10^{-3}	$(-2.161 \times 10^{-2}, 5.172 \times 10^{-2})$	2.247×10^{3}	1.000
Wheeling	-3.319×10^{-2}	5.220×10^{-4}	-3.351×10^{-2}	$(-7.490 \times 10^{-2}, 6.646 \times 10^{-3})$	1.559×10^{3}	1.008
Wichita Falls	-1.369×10^{-2}	2.734×10^{-4}	-1.345×10^{-2}	$(-4.236 \times 10^{-2}, 1.216 \times 10^{-2})$	2.586×10^{3}	1.001
Windsor	-1.073×10^{-2}	5.029×10^{-4}	-9.665×10^{-3}	$(-6.044 \times 10^{-2}, 3.800 \times 10^{-2})$	2.342×10^{3}	1.001
Winters	2.524×10^{-2}	4.923×10^{-4}	2.571×10^{-2}	$(-2.440 \times 10^{-2}, 7.297 \times 10^{-2})$	2.640×10^{3}	1.001
Wolcott	3.203×10^{-2}	5.309×10^{-4}	3.149×10^{-2}	$(-1.566 \times 10^{-2}, 8.683 \times 10^{-2})$	2.315×10^{3}	1.001
Woodland	5.687×10^{-3}	3.918×10^{-4}	4.401×10^{-3}	$(-3.802 \times 10^{-2}, 4.889 \times 10^{-2})$	2.886×10^{3}	0.999
Yankton	-1.163×10^{-2}	4.883×10^{-4}	-6.040×10^{-3}	$(-7.851 \times 10^{-2}, 3.413 \times 10^{-2})$	3.302×10^{3}	1.000
York	8.489×10^{-3}	3.510×10^{-4}	8.809×10^{-3}	$(-3.470 \times 10^{-2}, 4.421 \times 10^{-2})$	3.073×10^{3}	1.001
Youngstown	-2.269×10^{-2}	3.861×10^{-4}	-2.340×10^{-2}	$(-5.561 \times 10^{-2}, 1.287 \times 10^{-2})$	2.134×10^{3}	1.000
Zacate Creek	-1.105×10^{-2}	4.592×10^{-4}	-7.508×10^{-3}	$(-7.945 \times 10^{-2}, 4.422 \times 10^{-2})$	3.916×10^{3}	0.999

Table S7: Mean, Monte Carlo standard error, median, 95% credible interval, effective sample size, and $\hat{R}$ for coefficient on $\psi_7^{\sin}$ (weekly basis function) calculated over 4,000 posterior samples. Bolded intervals do not contain 0.

Site	Mean	MCSE	Median	95% CI	ESS	$\hat{R}$
Akron	-4.483×10^{-3}	3.441×10^{-4}	-3.444×10^{-3}	$(-3.873 \times 10^{-2}, 2.945 \times 10^{-2})$	2.389×10^{3}	1.001
Altamonte Springs	-1.187×10^{-2}	3.119×10^{-4}	-1.105×10^{-2}	$(-4.302 \times 10^{-2}, 1.580 \times 10^{-2})$	2.305×10^{3}	1.001
Ann Arbor	2.803×10^{-3}	3.088×10^{-4}	1.949×10^{-3}	$(-2.912 \times 10^{-2}, 3.601 \times 10^{-2})$	2.666×10^{3}	1.000
Aquia	-7.338×10^{-4}	1.924×10^{-4}	-4.390×10^{-4}	$(-2.008 \times 10^{-2}, 1.856 \times 10^{-2})$	2.637×10^{3}	1.003
Archie Elledge	1.102×10^{-2}	4.660×10^{-4}	9.617×10^{-3}	$(-3.427 \times 10^{-2}, 5.661 \times 10^{-2})$	2.350×10^{3}	1.001
Bangor	-2.961×10^{-2}	1.507×10^{-3}	-2.264×10^{-2}	$(-0.194, 0.120)$	2.564×10^{3}	1.000
Bayshore	-8.444×10^{-4}	3.996×10^{-4}	-4.477×10^{-4}	$(-4.642 \times 10^{-2}, 4.314 \times 10^{-2})$	3.077×10^{3}	0.999
Big Creek	3.058×10^{-2}	5.391×10^{-4}	2.961×10^{-2}	$(-1.619 \times 10^{-2}, 8.457 \times 10^{-2})$	2.252×10^{3}	1.001
Boege Alvarado (Fremont)	-2.546×10^{-2}	1.124×10^{-3}	-2.638×10^{-2}	$(-0.103, 6.553 \times 10^{-2})$	1.517×10^{3}	1.000
Boege Alvarado (Newark)	-1.605×10^{-2}	1.075×10^{-3}	-1.368×10^{-2}	$(-0.119, 8.703 \times 10^{-2})$	2.115×10^{3}	1.001
Boege Alvarado (Union City)	2.559×10^{-3}	1.266×10^{-3}	2.660×10^{-3}	$(-0.137, 0.138)$	2.550×10^{3}	1.001
Brunswick	1.749×10^{-2}	3.639×10^{-4}	1.718×10^{-2}	$(-1.689 \times 10^{-2}, 5.499 \times 10^{-2})$	2.517×10^{3}	1.000
CODIGA	-3.131×10^{-2}	3.147×10^{-4}	-3.145×10^{-2}	$(-6.352 \times 10^{-2}, 3.912 \times 10^{-4})$	2.786×10^{3}	1.000
Cahaba River	-4.112×10^{-2}	3.016×10^{-4}	-4.117×10^{-2}	$(\mathbf{-7.124 \times 10^{-2}, -8.594 \times 10^{-3}})$	2.819×10^{3}	1.001
Calera Creek	7.488×10^{-3}	3.477×10^{-4}	6.303×10^{-3}	$(-2.810 \times 10^{-2}, 4.389 \times 10^{-2})$	2.628×10^{3}	1.000
Camp Creek	3.368×10^{-3}	4.550×10^{-4}	3.234×10^{-3}	$(-4.306 \times 10^{-2}, 4.624 \times 10^{-2})$	2.453×10^{3}	1.000

Table S7: Mean, Monte Carlo standard error, median, 95% credible interval, effective sample size, and $\hat{R}$ for coefficient on $\psi_7^{\sin}$ (weekly basis function) calculated over 4,000 posterior samples. Bolded intervals do not contain 0.

Site	Mean	MCSE	Median	95% CI	ESS	$\hat{R}$
Capital Region	5.148×10^{-3}	2.973×10^{-4}	4.156×10^{-3}	$(-2.562 \times 10^{-2}, 3.632 \times 10^{-2})$	2.657×10^{3}	1.000
Carmel	1.259×10^{-2}	3.843×10^{-4}	9.914×10^{-3}	$(-2.584 \times 10^{-2}, 6.075 \times 10^{-2})$	3.216×10^{3}	1.000
Central Contra Costa	-7.972×10^{-3}	2.899×10^{-4}	-6.378×10^{-3}	$(-3.613 \times 10^{-2}, 1.588 \times 10^{-2})$	2.019×10^{3}	1.001
Central Marin	-1.822×10^{-2}	4.417×10^{-4}	-1.626×10^{-2}	$(-7.012 \times 10^{-2}, 2.837 \times 10^{-2})$	3.118×10^{3}	1.000
Central Marin (W Railroad)	-2.436×10^{-3}	4.340×10^{-4}	-1.369×10^{-3}	$(-5.462 \times 10^{-2}, 4.776 \times 10^{-2})$	3.497×10^{3}	0.999
Central Valley	-6.221×10^{-3}	1.857×10^{-4}	-5.401×10^{-3}	$(-2.816 \times 10^{-2}, 1.284 \times 10^{-2})$	3.017×10^{3}	1.001
Clark County	-2.398×10^{-2}	6.444×10^{-4}	-2.052×10^{-2}	$(-8.830 \times 10^{-2}, 3.001 \times 10^{-2})$	2.290×10^{3}	1.002
Clinton	3.402×10^{-3}	1.655×10^{-4}	2.797×10^{-3}	$(-1.226 \times 10^{-2}, 2.034 \times 10^{-2})$	2.446×10^{3}	1.000
Coastal	-3.538×10^{-2}	5.768×10^{-4}	-3.496×10^{-2}	$(-8.454 \times 10^{-2}, 1.135 \times 10^{-2})$	1.874×10^{3}	1.000
Coeur d'Alene	7.318×10^{-3}	2.342×10^{-4}	6.954×10^{-3}	$(-1.470 \times 10^{-2}, 3.003 \times 10^{-2})$	2.349×10^{3}	1.000
Coralville	1.578×10^{-3}	3.821×10^{-4}	8.733×10^{-4}	$(-4.252 \times 10^{-2}, 4.362 \times 10^{-2})$	3.071×10^{3}	1.002
Cumberland	-8.526×10^{-3}	5.224×10^{-4}	-6.533×10^{-3}	$(-7.188 \times 10^{-2}, 5.294 \times 10^{-2})$	3.480×10^{3}	1.000
DELCORA	1.164×10^{-2}	4.494×10^{-4}	9.336×10^{-3}	$(-2.842 \times 10^{-2}, 5.804 \times 10^{-2})$	2.306×10^{3}	1.001
Davis	8.643×10^{-4}	1.132×10^{-4}	7.526×10^{-4}	$(-1.081 \times 10^{-2}, 1.266 \times 10^{-2})$	2.626×10^{3}	1.001
Deer Island	2.126×10^{-2}	3.251×10^{-4}	2.131×10^{-2}	$(-5.855 \times 10^{-3}, 4.988 \times 10^{-2})$	2.104×10^{3}	1.001
Dillman Road	2.671×10^{-3}	4.316×10^{-4}	1.449×10^{-3}	$(-4.496 \times 10^{-2}, 4.941 \times 10^{-2})$	2.930×10^{3}	1.000
Dover	-4.045×10^{-2}	7.038×10^{-4}	-3.990×10^{-2}	$(-0.101, 1.171 \times 10^{-2})$	1.722×10^{3}	1.001
Duck Creek	-1.161×10^{-2}	2.370×10^{-4}	-1.131×10^{-2}	$(-3.332 \times 10^{-2}, 9.224 \times 10^{-3})$	2.251×10^{3}	1.000
E.W. Blom Point Loma	-1.767×10^{-2}	3.141×10^{-4}	-1.707×10^{-2}	$(-5.178 \times 10^{-2}, 1.214 \times 10^{-2})$	2.707×10^{3}	1.001
East Bay	1.557×10^{-2}	3.473×10^{-4}	1.431×10^{-2}	$(-1.255 \times 10^{-2}, 5.009 \times 10^{-2})$	2.243×10^{3}	1.004
Eastern	5.951×10^{-2}	3.142×10^{-4}	5.997×10^{-2}	$\mathbf{(2.256 \times 10^{-2}, 9.170 \times 10^{-2})}$	3.134×10^{3}	1.000
Ellis Creek	8.071×10^{-3}	3.322×10^{-4}	6.433×10^{-3}	$(-2.401 \times 10^{-2}, 4.239 \times 10^{-2})$	2.405×10^{3}	1.001
Esparto	-2.908×10^{-2}	5.406×10^{-4}	-2.745×10^{-2}	$(-8.594 \times 10^{-2}, 1.755 \times 10^{-2})$	2.460×10^{3}	1.000
Essex	8.083×10^{-3}	3.122×10^{-4}	7.155×10^{-3}	$(-2.457 \times 10^{-2}, 4.107 \times 10^{-2})$	2.777×10^{3}	1.000
Fairfield-Suisun	1.730×10^{-2}	2.777×10^{-4}	1.730×10^{-2}	$(-8.561 \times 10^{-3}, 4.425 \times 10^{-2})$	2.411×10^{3}	0.999
Five Mile Creek	2.763×10^{-3}	2.552×10^{-4}	1.903×10^{-3}	$(-2.581 \times 10^{-2}, 3.277 \times 10^{-2})$	3.129×10^{3}	1.000
Gainesville	-1.181×10^{-2}	3.499×10^{-4}	-1.054×10^{-2}	$(-4.818 \times 10^{-2}, 2.332 \times 10^{-2})$	2.666×10^{3}	1.001
Garland Rowlett Creek	9.466×10^{-3}	2.439×10^{-4}	9.171×10^{-3}	$(-1.391 \times 10^{-2}, 3.277 \times 10^{-2})$	2.452×10^{3}	1.004
Glenbard	-9.944×10^{-3}	3.578×10^{-4}	-8.841×10^{-3}	$(-4.494 \times 10^{-2}, 2.184 \times 10^{-2})$	2.267×10^{3}	0.999
Grandville	-1.016×10^{-2}	3.139×10^{-4}	-9.061×10^{-3}	$(-3.955 \times 10^{-2}, 1.718 \times 10^{-2})$	2.108×10^{3}	1.001
Hagerstown	1.818×10^{-2}	5.284×10^{-4}	1.703×10^{-2}	$(-3.574 \times 10^{-2}, 7.586 \times 10^{-2})$	2.762×10^{3}	1.001
Hall Street	-3.521×10^{-2}	3.028×10^{-4}	-3.532×10^{-2}	$\mathbf{(-6.915 \times 10^{-2}, -1.279 \times 10^{-3})}$	3.133×10^{3}	1.000
Hamlin	1.732×10^{-3}	4.353×10^{-4}	7.609×10^{-4}	$(-4.452 \times 10^{-2}, 5.447 \times 10^{-2})$	2.980×10^{3}	1.000
Harrison	-1.442×10^{-2}	6.085×10^{-4}	-1.156×10^{-2}	$(-8.164 \times 10^{-2}, 4.814 \times 10^{-2})$	2.800×10^{3}	1.000
Hillsville	-9.148×10^{-3}	2.030×10^{-4}	-8.998×10^{-3}	$(-2.700 \times 10^{-2}, 8.928 \times 10^{-3})$	2.147×10^{3}	1.002
Hollister	9.483×10^{-3}	5.084×10^{-4}	7.947×10^{-3}	$(-3.151 \times 10^{-2}, 5.269 \times 10^{-2})$	1.796×10^{3}	1.001
Hollywood Road	3.239×10^{-2}	7.097×10^{-4}	3.125×10^{-2}	$(-3.190 \times 10^{-2}, 0.101)$	2.482×10^{3}	1.000
Hyperion	3.728×10^{-2}	4.529×10^{-4}	3.705×10^{-2}	$(-6.118 \times 10^{-4}, 7.898 \times 10^{-2})$	2.137×10^{3}	1.001
JB Latham	6.607×10^{-2}	5.746×10^{-4}	6.765×10^{-2}	$\mathbf{(8.240 \times 10^{-3}, 0.116)}$	2.207×10^{3}	1.001
Jackson	8.800×10^{-3}	1.913×10^{-4}	8.161×10^{-3}	$(-8.031 \times 10^{-3}, 2.852 \times 10^{-2})$	2.425×10^{3}	1.000
Jeffersonville	3.145×10^{-2}	5.025×10^{-4}	3.124×10^{-2}	$(-9.800 \times 10^{-3}, 7.494 \times 10^{-2})$	1.928×10^{3}	1.002
John M. Asplund	3.461×10^{-2}	9.619×10^{-4}	2.989×10^{-2}	$(-5.869 \times 10^{-2}, 0.143)$	2.855×10^{3}	1.000
Johnnie Mosley	2.442×10^{-2}	4.309×10^{-4}	2.408×10^{-2}	$(-1.263 \times 10^{-2}, 6.497 \times 10^{-2})$	2.208×10^{3}	1.000
Johns Creek	-7.085×10^{-3}	5.283×10^{-4}	-4.325×10^{-3}	$(-6.281 \times 10^{-2}, 3.954 \times 10^{-2})$	2.263×10^{3}	1.001
Joint	1.256×10^{-2}	2.450×10^{-4}	1.229×10^{-2}	$(-8.250 \times 10^{-3}, 3.532 \times 10^{-2})$	2.179×10^{3}	1.003
Kansas City	-6.855×10^{-3}	3.418×10^{-4}	-5.069×10^{-3}	$(-4.725 \times 10^{-2}, 2.839 \times 10^{-2})$	3.016×10^{3}	1.000
Kaw Point	-1.654×10^{-2}	3.456×10^{-4}	-1.615×10^{-2}	$(-5.127 \times 10^{-2}, 1.508 \times 10^{-2})$	2.409×10^{3}	1.002
Lancaster	-1.865×10^{-2}	5.455×10^{-4}	-1.699×10^{-2}	$(-6.857 \times 10^{-2}, 2.580 \times 10^{-2})$	1.958×10^{3}	1.000
Lander Street	-7.612×10^{-4}	3.699×10^{-4}	-3.461×10^{-4}	$(-4.516 \times 10^{-2}, 4.313 \times 10^{-2})$	3.293×10^{3}	1.000
Las Gallinas	-1.660×10^{-2}	3.473×10^{-4}	-1.543×10^{-2}	$(-5.369 \times 10^{-2}, 1.529 \times 10^{-2})$	2.604×10^{3}	1.000

Table S7: Mean, Monte Carlo standard error, median, 95% credible interval, effective sample size, and $\hat{R}$ for coefficient on $\psi_7^{\sin}$ (weekly basis function) calculated over 4,000 posterior samples. Bolded intervals do not contain 0.

Site	Mean	MCSE	Median	95% CI	ESS	$\hat{R}$
Lawrence Kansas	1.387×10^{-2}	2.597×10^{-4}	1.366×10^{-2}	$(-9.117 \times 10^{-3}, 3.859 \times 10^{-2})$	2.285×10^{3}	1.001
Little Falls Run	-2.510×10^{-3}	1.741×10^{-4}	-1.838×10^{-3}	$(-2.425 \times 10^{-2}, 1.792 \times 10^{-2})$	3.567×10^{3}	0.999
Little River	3.296×10^{-2}	6.586×10^{-4}	3.215×10^{-2}	$(-1.876 \times 10^{-2}, 9.103 \times 10^{-2})$	1.817×10^{3}	1.002
Lompoc	2.692×10^{-2}	4.988×10^{-4}	2.576×10^{-2}	$(-1.153 \times 10^{-2}, 7.300 \times 10^{-2})$	2.062×10^{3}	1.001
Los Banos	-1.433×10^{-2}	4.335×10^{-4}	-1.333×10^{-2}	$(-6.021 \times 10^{-2}, 2.988 \times 10^{-2})$	2.773×10^{3}	1.000
Loxahatchee	-8.156×10^{-3}	3.033×10^{-4}	-7.556×10^{-3}	$(-3.961 \times 10^{-2}, 2.411 \times 10^{-2})$	2.780×10^{3}	1.001
MDWASD Central	-8.039×10^{-3}	4.489×10^{-4}	-7.013×10^{-3}	$(-5.117 \times 10^{-2}, 3.486 \times 10^{-2})$	2.278×10^{3}	1.001
MDWASD North	1.845×10^{-2}	4.921×10^{-4}	1.594×10^{-2}	$(-2.843 \times 10^{-2}, 7.514 \times 10^{-2})$	2.744×10^{3}	1.000
MDWASD South	2.344×10^{-2}	4.008×10^{-4}	2.327×10^{-2}	$(-1.294 \times 10^{-2}, 6.229 \times 10^{-2})$	2.381×10^{3}	1.001
Madera	-3.597×10^{-3}	2.757×10^{-4}	-2.783×10^{-3}	$(-3.255 \times 10^{-2}, 2.280 \times 10^{-2})$	2.513×10^{3}	1.000
Mankato	9.237×10^{-3}	1.889×10^{-4}	8.942×10^{-3}	$(-9.564 \times 10^{-3}, 2.926 \times 10^{-2})$	2.656×10^{3}	1.001
Markshalltown	0.125	5.855×10^{-4}	0.125	$\mathbf{(5.876 \times 10^{-2}, 0.187)}$	3.099×10^{3}	1.000
Marlay Taylor	-8.655×10^{-3}	3.700×10^{-4}	-7.586×10^{-3}	$(-4.821 \times 10^{-2}, 2.822 \times 10^{-2})$	2.620×10^{3}	1.000
Merced	7.539×10^{-3}	1.876×10^{-4}	7.088×10^{-3}	$(-1.056 \times 10^{-2}, 2.769 \times 10^{-2})$	2.623×10^{3}	1.001
Mid-Coastside	2.852×10^{-3}	2.850×10^{-4}	2.391×10^{-3}	$(-2.712 \times 10^{-2}, 3.365 \times 10^{-2})$	2.792×10^{3}	1.000
Modesto's Sutter	2.158×10^{-2}	2.086×10^{-4}	2.163×10^{-2}	$\mathbf{(2.954 \times 10^{-3}, 3.877 \times 10^{-2})}$	1.903×10^{3}	1.002
Montpelier	1.738×10^{-3}	5.735×10^{-4}	1.145×10^{-3}	$(-5.605 \times 10^{-2}, 5.534 \times 10^{-2})$	2.382×10^{3}	1.000
Monterey One	-2.224×10^{-2}	5.084×10^{-4}	-2.078×10^{-2}	$(-7.404 \times 10^{-2}, 2.184 \times 10^{-2})$	2.377×10^{3}	1.000
Morris Forman	-1.125×10^{-2}	3.823×10^{-4}	-9.890×10^{-3}	$(-4.858 \times 10^{-2}, 2.237 \times 10^{-2})$	2.278×10^{3}	1.000
Mt. Pleasant	-3.025×10^{-2}	6.454×10^{-4}	-2.873×10^{-2}	$(-9.284 \times 10^{-2}, 2.555 \times 10^{-2})$	2.228×10^{3}	1.003
Muscatine	-3.309×10^{-2}	5.617×10^{-4}	-3.208×10^{-2}	$(-8.992 \times 10^{-2}, 1.507 \times 10^{-2})$	2.277×10^{3}	1.000
Norhtwest Water	4.865×10^{-3}	2.879×10^{-4}	3.775×10^{-3}	$(-2.396 \times 10^{-2}, 3.429 \times 10^{-2})$	2.508×10^{3}	0.999
North Water	-2.876×10^{-3}	2.421×10^{-4}	-2.187×10^{-3}	$(-3.202 \times 10^{-2}, 2.491 \times 10^{-2})$	3.298×10^{3}	1.000
Novato	-1.002×10^{-3}	3.111×10^{-4}	-4.461×10^{-4}	$(-3.211 \times 10^{-2}, 3.051 \times 10^{-2})$	2.531×10^{3}	1.000
Ocean	7.954×10^{-2}	1.066×10^{-2}	6.741×10^{-2}	$(-0.796, 0.967)$	1.556×10^{3}	1.003
Oceanside	2.759×10^{-3}	1.096×10^{-4}	2.270×10^{-3}	$(-8.294 \times 10^{-3}, 1.476 \times 10^{-2})$	2.753×10^{3}	1.000
Ottumwa	-6.166×10^{-3}	3.412×10^{-4}	-5.080×10^{-3}	$(-4.211 \times 10^{-2}, 2.907 \times 10^{-2})$	2.726×10^{3}	1.002
Palo Alto	9.518×10^{-3}	1.079×10^{-4}	9.587×10^{-3}	$(-3.936 \times 10^{-4}, 1.933 \times 10^{-2})$	2.215×10^{3}	1.003
Parker North	-5.374×10^{-2}	4.966×10^{-4}	-5.465×10^{-2}	$\mathbf{(-9.496 \times 10^{-2}, -8.604 \times 10^{-3})}$	1.958×10^{3}	1.001
Parker South	-1.377×10^{-2}	5.201×10^{-4}	-1.253×10^{-2}	$(-5.964 \times 10^{-2}, 2.985 \times 10^{-2})$	1.941×10^{3}	1.001
Paso Robles	-1.134×10^{-2}	3.048×10^{-4}	-1.025×10^{-2}	$(-4.033 \times 10^{-2}, 1.331 \times 10^{-2})$	2.085×10^{3}	1.001
Passaic Valley	-8.868×10^{-3}	3.761×10^{-4}	-8.240×10^{-3}	$(-4.267 \times 10^{-2}, 2.320 \times 10^{-2})$	1.862×10^{3}	1.001
Penacook	-1.525×10^{-2}	3.847×10^{-4}	-1.326×10^{-2}	$(-5.515 \times 10^{-2}, 1.621 \times 10^{-2})$	2.296×10^{3}	1.000
Portland	2.478×10^{-2}	4.861×10^{-4}	2.420×10^{-2}	$(-1.339 \times 10^{-2}, 6.820 \times 10^{-2})$	1.906×10^{3}	1.001
Provo City	1.514×10^{-2}	6.025×10^{-4}	1.334×10^{-2}	$(-4.127 \times 10^{-2}, 7.467 \times 10^{-2})$	2.300×10^{3}	1.000
RM Clayton	3.350×10^{-2}	4.518×10^{-4}	3.368×10^{-2}	$(-8.696 \times 10^{-3}, 7.769 \times 10^{-2})$	2.551×10^{3}	1.005
Red Wing	2.859×10^{-3}	3.973×10^{-4}	2.078×10^{-3}	$(-4.071 \times 10^{-2}, 4.586 \times 10^{-2})$	2.933×10^{3}	1.000
Regional	-4.946×10^{-2}	3.610×10^{-4}	-4.922×10^{-2}	$\mathbf{(-9.114 \times 10^{-2}, -8.969 \times 10^{-3})}$	3.395×10^{3}	1.000
Regional No. 1	-2.285×10^{-3}	3.752×10^{-4}	-1.227×10^{-3}	$(-4.198 \times 10^{-2}, 3.532 \times 10^{-2})$	2.599×10^{3}	1.001
River Road	-3.233×10^{-2}	6.768×10^{-4}	-3.117×10^{-2}	$(-0.102, 2.452 \times 10^{-2})$	2.255×10^{3}	1.000
Riverside	4.477×10^{-2}	4.747×10^{-4}	4.489×10^{-2}	$(-7.674 \times 10^{-4}, 9.769 \times 10^{-2})$	2.875×10^{3}	1.000
Rochester	-2.269×10^{-2}	3.011×10^{-4}	-2.248×10^{-2}	$(-5.303 \times 10^{-2}, 5.393 \times 10^{-3})$	2.629×10^{3}	1.000
SJRA No. 1	-3.623×10^{-2}	5.335×10^{-4}	-3.631×10^{-2}	$(-8.682 \times 10^{-2}, 1.163 \times 10^{-2})$	2.408×10^{3}	1.000
SJRA No. 2	9.973×10^{-3}	4.204×10^{-4}	7.833×10^{-3}	$(-3.095 \times 10^{-2}, 5.690 \times 10^{-2})$	2.772×10^{3}	1.001
SJRA No. 3	-4.146×10^{-3}	5.043×10^{-4}	-2.857×10^{-3}	$(-5.606 \times 10^{-2}, 5.077 \times 10^{-2})$	2.749×10^{3}	1.001
Sacramento	3.461×10^{-3}	7.824×10^{-5}	3.314×10^{-3}	$(-3.549 \times 10^{-3}, 1.121 \times 10^{-2})$	2.340×10^{3}	1.000
Salina	1.527×10^{-2}	3.420×10^{-4}	1.482×10^{-2}	$(-1.018 \times 10^{-2}, 4.410 \times 10^{-2})$	1.743×10^{3}	1.002
San Francisco	-2.401×10^{-2}	1.224×10^{-4}	-2.397×10^{-2}	$\mathbf{(-3.780 \times 10^{-2}, -1.025 \times 10^{-2})}$	3.292×10^{3}	1.000
San Jose-Santa Clara	-3.485×10^{-3}	9.114×10^{-5}	-3.335×10^{-3}	$(-1.198 \times 10^{-2}, 4.107 \times 10^{-3})$	1.998×10^{3}	1.001
San Leandro	-6.156×10^{-3}	5.509×10^{-4}	-4.835×10^{-3}	$(-6.873 \times 10^{-2}, 5.262 \times 10^{-2})$	2.882×10^{3}	1.000

Table S7: Mean, Monte Carlo standard error, median, 95% credible interval, effective sample size, and $\hat{R}$ for coefficient on $\psi_7^{\sin}$ (weekly basis function) calculated over 4,000 posterior samples. Bolded intervals do not contain 0.

Site	Mean	MCSE	Median	95% CI	ESS	$\hat{R}$
San Mateo & Estero	2.369×10^{-3}	2.967×10^{-4}	1.935×10^{-3}	$(-2.838 \times 10^{-2}, 3.236 \times 10^{-2})$	2.628×10^{3}	0.999
Santa Cruz (City)	-2.369×10^{-2}	3.522×10^{-4}	-2.317×10^{-2}	$(-5.869 \times 10^{-2}, 7.288 \times 10^{-3})$	2.446×10^{3}	1.001
Santa Cruz (County)	-1.608×10^{-2}	2.849×10^{-4}	-1.567×10^{-2}	$(-4.476 \times 10^{-2}, 9.655 \times 10^{-3})$	2.461×10^{3}	1.000
Santa Rosa	8.413×10^{-3}	2.265×10^{-4}	8.271×10^{-3}	$(-1.356 \times 10^{-2}, 3.006 \times 10^{-2})$	2.468×10^{3}	1.000
Sausalito-Marin	-7.924×10^{-3}	4.165×10^{-4}	-6.145×10^{-3}	$(-5.212 \times 10^{-2}, 3.134 \times 10^{-2})$	2.513×10^{3}	1.002
Seaford	2.048×10^{-2}	3.209×10^{-4}	1.978×10^{-2}	$(-8.865 \times 10^{-3}, 5.428 \times 10^{-2})$	2.687×10^{3}	1.000
Silicon Valley	-6.223×10^{-3}	1.538×10^{-4}	-6.016×10^{-3}	$(-1.971 \times 10^{-2}, 6.692 \times 10^{-3})$	1.970×10^{3}	1.001
Somerset Raritan	2.740×10^{-2}	1.186×10^{-3}	2.222×10^{-2}	$(-8.575 \times 10^{-2}, 0.146)$	2.317×10^{3}	1.000
Soscol	-5.034×10^{-3}	1.905×10^{-4}	-4.216×10^{-3}	$(-2.623 \times 10^{-2}, 1.511 \times 10^{-2})$	2.944×10^{3}	1.000
South Bay	4.004×10^{-2}	6.052×10^{-4}	3.781×10^{-2}	$(-1.130 \times 10^{-2}, 0.106)$	2.503×10^{3}	0.999
South Bend	-8.207×10^{-3}	3.789×10^{-4}	-6.901×10^{-3}	$(-4.789 \times 10^{-2}, 3.100 \times 10^{-2})$	2.653×10^{3}	1.000
South Burlington	-2.118×10^{-2}	4.573×10^{-4}	-2.042×10^{-2}	$(-6.846 \times 10^{-2}, 2.078 \times 10^{-2})$	2.422×10^{3}	1.000
South Columbus	-2.730×10^{-3}	3.158×10^{-4}	-2.211×10^{-3}	$(-3.241 \times 10^{-2}, 2.744 \times 10^{-2})$	2.240×10^{3}	1.004
South County	-1.576×10^{-2}	1.574×10^{-4}	-1.536×10^{-2}	$(-3.351 \times 10^{-2}, 6.510 \times 10^{-4})$	3.125×10^{3}	1.001
South Laredo	3.174×10^{-2}	4.897×10^{-4}	3.155×10^{-2}	$(-1.182 \times 10^{-2}, 8.013 \times 10^{-2})$	2.366×10^{3}	1.002
South Monmouth	-3.060×10^{-2}	3.752×10^{-4}	-3.040×10^{-2}	$(-7.022 \times 10^{-2}, 4.976 \times 10^{-3})$	2.810×10^{3}	0.999
South River	-3.009×10^{-2}	4.184×10^{-4}	-2.996×10^{-2}	$(-7.108 \times 10^{-2}, 9.370 \times 10^{-3})$	2.528×10^{3}	1.000
South Water	1.856×10^{-2}	3.796×10^{-4}	1.819×10^{-2}	$(-1.373 \times 10^{-2}, 5.261 \times 10^{-2})$	2.040×10^{3}	0.999
Southern Marin	-1.009×10^{-2}	2.988×10^{-4}	-8.923×10^{-3}	$(-4.056 \times 10^{-2}, 1.637 \times 10^{-2})$	2.360×10^{3}	1.000
St. Cloud	7.693×10^{-3}	3.989×10^{-4}	5.769×10^{-3}	$(-3.421 \times 10^{-2}, 5.338 \times 10^{-2})$	3.086×10^{3}	1.001
Sunnyvale	1.658×10^{-3}	1.036×10^{-4}	1.385×10^{-3}	$(-7.909 \times 10^{-3}, 1.170 \times 10^{-2})$	2.176×10^{3}	1.002
Traverse City	-2.647×10^{-2}	6.905×10^{-4}	-2.364×10^{-2}	$(-9.531 \times 10^{-2}, 3.444 \times 10^{-2})$	2.368×10^{3}	1.000
Turkey Creek	-1.473×10^{-2}	5.143×10^{-4}	-1.330×10^{-2}	$(-6.229 \times 10^{-2}, 2.873 \times 10^{-2})$	2.031×10^{3}	1.001
Turlock	8.193×10^{-2}	4.736×10^{-4}	8.239×10^{-2}	$\mathbf{(2.682 \times 10^{-2}, 0.135)}$	3.365×10^{3}	1.000
Upper Blackstone	1.471×10^{-2}	5.354×10^{-4}	1.271×10^{-2}	$(-3.811 \times 10^{-2}, 7.256 \times 10^{-2})$	2.704×10^{3}	1.001
Utoy Creek	2.014×10^{-2}	4.656×10^{-4}	1.893×10^{-2}	$(-1.190 \times 10^{-2}, 5.979 \times 10^{-2})$	1.603×10^{3}	1.003
Vallejo	-1.009×10^{-2}	4.397×10^{-4}	-8.046×10^{-3}	$(-5.845 \times 10^{-2}, 3.395 \times 10^{-2})$	2.712×10^{3}	1.000
Valley	-9.763×10^{-3}	2.370×10^{-4}	-8.998×10^{-3}	$(-3.669 \times 10^{-2}, 1.405 \times 10^{-2})$	3.014×10^{3}	1.000
Valley Creek	9.921×10^{-3}	3.261×10^{-4}	8.968×10^{-3}	$(-1.924 \times 10^{-2}, 4.244 \times 10^{-2})$	2.341×10^{3}	0.999
Village Creek	-4.865×10^{-2}	3.445×10^{-4}	-4.864×10^{-2}	$\mathbf{(-8.874 \times 10^{-2}, -8.838 \times 10^{-3})}$	3.412×10^{3}	1.001
Warren	2.134×10^{-2}	5.180×10^{-4}	1.882×10^{-2}	$(-1.916 \times 10^{-2}, 7.119 \times 10^{-2})$	2.035×10^{3}	1.002
Weaton	1.575×10^{-3}	2.020×10^{-4}	1.189×10^{-3}	$(-1.916 \times 10^{-2}, 2.258 \times 10^{-2})$	2.559×10^{3}	1.000
West Boise	-2.400×10^{-2}	5.034×10^{-4}	-2.426×10^{-2}	$(-6.948 \times 10^{-2}, 1.885 \times 10^{-2})$	2.120×10^{3}	1.002
West County	-8.832×10^{-3}	4.065×10^{-4}	-7.185×10^{-3}	$(-5.467 \times 10^{-2}, 3.181 \times 10^{-2})$	2.835×10^{3}	1.001
Wheeling	-1.082×10^{-2}	2.936×10^{-4}	-1.011×10^{-2}	$(-3.911 \times 10^{-2}, 1.546 \times 10^{-2})$	2.215×10^{3}	1.001
Wichita Falls	1.002×10^{-2}	2.669×10^{-4}	9.534×10^{-3}	$(-1.620 \times 10^{-2}, 3.694 \times 10^{-2})$	2.565×10^{3}	1.000
Windsor	1.961×10^{-3}	6.255×10^{-4}	1.312×10^{-3}	$(-5.486 \times 10^{-2}, 6.123 \times 10^{-2})$	2.090×10^{3}	1.001
Winters	1.735×10^{-2}	5.676×10^{-4}	1.497×10^{-2}	$(-3.044 \times 10^{-2}, 6.997 \times 10^{-2})$	2.008×10^{3}	1.000
Wolcott	-1.743×10^{-3}	4.290×10^{-4}	-1.129×10^{-3}	$(-5.013 \times 10^{-2}, 4.530 \times 10^{-2})$	2.939×10^{3}	1.000
Woodland	-4.051×10^{-2}	4.523×10^{-4}	-4.047×10^{-2}	$(-8.711 \times 10^{-2}, 5.210 \times 10^{-3})$	2.853×10^{3}	1.000
Yankton	7.342×10^{-2}	4.259×10^{-4}	7.308×10^{-2}	$\mathbf{(2.235 \times 10^{-2}, 0.127)}$	3.960×10^{3}	1.001
York	1.495×10^{-2}	4.925×10^{-4}	1.320×10^{-2}	$(-3.380 \times 10^{-2}, 6.744 \times 10^{-2})$	2.692×10^{3}	1.000
Youngstown	1.454×10^{-2}	3.043×10^{-4}	1.360×10^{-2}	$(-1.463 \times 10^{-2}, 4.835 \times 10^{-2})$	2.805×10^{3}	1.003
Zacate Creek	1.726×10^{-2}	4.787×10^{-4}	1.583×10^{-2}	$(-2.967 \times 10^{-2}, 6.601 \times 10^{-2})$	2.657×10^{3}	1.001

Table S8: Mean, Monte Carlo standard error, median, 95% credible interval, effective sample size, and $\hat{R}$ for coefficient on $\psi_7^{\cos}$ (weekly basis function) calculated over 4,000 posterior samples. Bolded intervals do not contain 0.

Site	Mean	MCSE	Median	95% CI	ESS	$\hat{R}$
Akron	6.893×10^{-3}	3.473×10^{-4}	5.073×10^{-3}	$(-2.468 \times 10^{-2}, 4.313 \times 10^{-2})$	2.460×10^{3}	1.000
Altamonte Springs	-1.886×10^{-2}	3.562×10^{-4}	-1.796×10^{-2}	$(-5.711 \times 10^{-2}, 1.246 \times 10^{-2})$	2.609×10^{3}	1.002
Ann Arbor	-8.505×10^{-3}	4.353×10^{-4}	-6.760×10^{-3}	$(-5.155 \times 10^{-2}, 2.963 \times 10^{-2})$	2.076×10^{3}	1.000
Aquia	2.632×10^{-2}	2.109×10^{-4}	2.578×10^{-2}	$\mathbf{(3.697 \times 10^{-3}, 5.131 \times 10^{-2})}$	3.340×10^{3}	1.000
Archie Elledge	-3.569×10^{-2}	4.994×10^{-4}	-3.543×10^{-2}	$(-8.163 \times 10^{-2}, 5.958 \times 10^{-3})$	2.124×10^{3}	1.000
Bangor	6.378×10^{-2}	1.534×10^{-3}	6.046×10^{-2}	$(-9.286 \times 10^{-2}, 0.228)$	2.681×10^{3}	1.000
Bayshore	-6.056×10^{-3}	5.074×10^{-4}	-2.655×10^{-3}	$(-6.703 \times 10^{-2}, 4.452 \times 10^{-2})$	2.955×10^{3}	1.001
Big Creek	1.450×10^{-2}	4.693×10^{-4}	1.121×10^{-2}	$(-2.453 \times 10^{-2}, 6.552 \times 10^{-2})$	2.432×10^{3}	1.001
Boege Alvarado (Fremont)	-6.108×10^{-2}	1.136×10^{-3}	-6.286×10^{-2}	$(-0.146, 2.480 \times 10^{-2})$	1.538×10^{3}	1.000
Boege Alvarado (Newark)	-2.893×10^{-2}	1.066×10^{-3}	-2.641×10^{-2}	$(-0.131, 6.776 \times 10^{-2})$	2.063×10^{3}	1.001
Boege Alvarado (Union City)	-2.047×10^{-2}	1.262×10^{-3}	-1.683×10^{-2}	$(-0.159, 0.110)$	2.561×10^{3}	1.001
Brunswick	-2.094×10^{-2}	3.636×10^{-4}	-2.052×10^{-2}	$(-5.771 \times 10^{-2}, 1.133 \times 10^{-2})$	2.466×10^{3}	1.002
CODIGA	8.574×10^{-3}	2.861×10^{-4}	7.602×10^{-3}	$(-1.989 \times 10^{-2}, 3.975 \times 10^{-2})$	2.864×10^{3}	1.001
Cahaba River	-1.187×10^{-2}	3.182×10^{-4}	-1.059×10^{-2}	$(-4.491 \times 10^{-2}, 1.681 \times 10^{-2})$	2.441×10^{3}	1.001
Calera Creek	1.960×10^{-2}	4.080×10^{-4}	1.888×10^{-2}	$(-1.925 \times 10^{-2}, 6.141 \times 10^{-2})$	2.606×10^{3}	1.000
Camp Creek	-6.064×10^{-3}	5.023×10^{-4}	-4.847×10^{-3}	$(-5.207 \times 10^{-2}, 3.833 \times 10^{-2})$	1.985×10^{3}	1.000
Capital Region	-2.924×10^{-2}	3.615×10^{-4}	-2.954×10^{-2}	$(-6.426 \times 10^{-2}, 5.371 \times 10^{-3})$	2.555×10^{3}	1.002
Carmel	-3.723×10^{-3}	4.119×10^{-4}	-2.099×10^{-3}	$(-4.729 \times 10^{-2}, 3.438 \times 10^{-2})$	2.417×10^{3}	1.000
Central Contra Costa	-1.611×10^{-2}	2.988×10^{-4}	-1.538×10^{-2}	$(-4.413 \times 10^{-2}, 6.655 \times 10^{-3})$	1.959×10^{3}	1.001
Central Marin	-5.643×10^{-2}	5.591×10^{-4}	-5.700×10^{-2}	$\mathbf{(-0.109, -5.284 \times 10^{-4})}$	2.498×10^{3}	1.001
Central Marin (W Railroad)	1.048×10^{-2}	4.836×10^{-4}	8.588×10^{-3}	$(-3.730 \times 10^{-2}, 6.006 \times 10^{-2})$	2.588×10^{3}	1.000
Central Valley	-2.474×10^{-2}	2.532×10^{-4}	-2.431×10^{-2}	$\mathbf{(-5.144 \times 10^{-2}, -9.985 \times 10^{-6})}$	2.805×10^{3}	1.001
Clark County	2.190×10^{-2}	5.262×10^{-4}	2.079×10^{-2}	$(-2.022 \times 10^{-2}, 6.968 \times 10^{-2})$	1.962×10^{3}	1.002
Clinton	-1.255×10^{-3}	1.634×10^{-4}	-9.561×10^{-4}	$(-1.753 \times 10^{-2}, 1.487 \times 10^{-2})$	2.574×10^{3}	1.002
Coastal	-3.465×10^{-3}	3.921×10^{-4}	-2.903×10^{-3}	$(-4.141 \times 10^{-2}, 3.586 \times 10^{-2})$	2.435×10^{3}	1.000
Coeur d'Alene	-8.774×10^{-3}	2.184×10^{-4}	-7.973×10^{-3}	$(-3.240 \times 10^{-2}, 1.151 \times 10^{-2})$	2.642×10^{3}	1.001
Coralville	3.289×10^{-2}	5.603×10^{-4}	3.264×10^{-2}	$(-1.460 \times 10^{-2}, 8.254 \times 10^{-2})$	1.980×10^{3}	1.000
Cumberland	-8.629×10^{-2}	5.317×10^{-4}	-8.684×10^{-2}	$\mathbf{(-0.147, -2.150 \times 10^{-2})}$	3.726×10^{3}	0.999
DELCORA	8.905×10^{-3}	3.977×10^{-4}	7.550×10^{-3}	$(-2.670 \times 10^{-2}, 4.705 \times 10^{-2})$	2.214×10^{3}	1.000
Davis	-1.896×10^{-2}	1.055×10^{-4}	-1.904×10^{-2}	$\mathbf{(-3.063 \times 10^{-2}, -6.914 \times 10^{-3})}$	3.230×10^{3}	1.001
Deer Island	-1.383×10^{-2}	2.956×10^{-4}	-1.276×10^{-2}	$(-4.608 \times 10^{-2}, 1.256 \times 10^{-2})$	2.728×10^{3}	0.999
Dillman Road	1.546×10^{-3}	4.273×10^{-4}	9.322×10^{-4}	$(-4.346 \times 10^{-2}, 4.822 \times 10^{-2})$	2.836×10^{3}	0.999
Dover	-3.595×10^{-2}	7.500×10^{-4}	-3.546×10^{-2}	$(-9.607 \times 10^{-2}, 1.724 \times 10^{-2})$	1.567×10^{3}	1.002
Duck Creek	-1.231×10^{-2}	2.564×10^{-4}	-1.178×10^{-2}	$(-3.665 \times 10^{-2}, 8.503 \times 10^{-3})$	2.156×10^{3}	1.000
E.W. Blom Point Loma	4.715×10^{-3}	3.546×10^{-4}	4.534×10^{-3}	$(-3.616 \times 10^{-2}, 4.257 \times 10^{-2})$	2.934×10^{3}	1.000
East Bay	-8.196×10^{-3}	3.034×10^{-4}	-6.893×10^{-3}	$(-3.920 \times 10^{-2}, 2.139 \times 10^{-2})$	2.497×10^{3}	1.002
Eastern	-9.561×10^{-3}	2.751×10^{-4}	-8.561×10^{-3}	$(-4.130 \times 10^{-2}, 1.760 \times 10^{-2})$	2.882×10^{3}	1.000
Ellis Creek	-7.526×10^{-2}	3.214×10^{-4}	-7.520×10^{-2}	$\mathbf{(-0.110, -3.943 \times 10^{-2})}$	3.121×10^{3}	1.000
Esparto	1.957×10^{-2}	5.427×10^{-4}	1.748×10^{-2}	$(-2.914 \times 10^{-2}, 7.516 \times 10^{-2})$	2.441×10^{3}	1.000
Essex	3.642×10^{-2}	4.596×10^{-4}	3.599×10^{-2}	$(-3.721 \times 10^{-4}, 7.573 \times 10^{-2})$	1.846×10^{3}	1.000
Fairfield-Suisun	-2.309×10^{-2}	2.295×10^{-4}	-2.293×10^{-2}	$\mathbf{(-4.683 \times 10^{-2}, -3.085 \times 10^{-4})}$	2.821×10^{3}	0.999
Five Mile Creek	-1.987×10^{-4}	2.385×10^{-4}	-2.264×10^{-4}	$(-2.707 \times 10^{-2}, 2.659 \times 10^{-2})$	3.012×10^{3}	1.000
Gainesville	-1.405×10^{-2}	5.020×10^{-4}	-1.299×10^{-2}	$(-6.120 \times 10^{-2}, 3.187 \times 10^{-2})$	2.254×10^{3}	1.000
Garland Rowlett Creek	-7.758×10^{-3}	2.416×10^{-4}	-7.017×10^{-3}	$(-3.276 \times 10^{-2}, 1.489 \times 10^{-2})$	2.547×10^{3}	1.001
Glenbard	-9.446×10^{-3}	3.884×10^{-4}	-7.671×10^{-3}	$(-4.867 \times 10^{-2}, 2.434 \times 10^{-2})$	2.188×10^{3}	0.999
Grandville	-3.075×10^{-2}	3.091×10^{-4}	-3.138×10^{-2}	$\mathbf{(-5.839 \times 10^{-2}, -1.138 \times 10^{-3})}$	2.283×10^{3}	1.001
Hagerstown	-2.495×10^{-2}	7.719×10^{-4}	-2.309×10^{-2}	$(-8.322 \times 10^{-2}, 2.647 \times 10^{-2})$	1.391×10^{3}	1.001
Hall Street	-4.093×10^{-2}	3.378×10^{-4}	-4.107×10^{-2}	$\mathbf{(-7.690 \times 10^{-2}, -3.498 \times 10^{-3})}$	3.020×10^{3}	0.999
Hamlin	-1.129×10^{-2}	5.327×10^{-4}	-9.724×10^{-3}	$(-6.135 \times 10^{-2}, 3.575 \times 10^{-2})$	2.103×10^{3}	1.000
Harrison	-8.252×10^{-3}	5.941×10^{-4}	-7.273×10^{-3}	$(-7.076 \times 10^{-2}, 5.760 \times 10^{-2})$	2.836×10^{3}	1.000

Table S8: Mean, Monte Carlo standard error, median, 95% credible interval, effective sample size, and $\hat{R}$ for coefficient on $\psi_7^{\cos}$ (weekly basis function) calculated over 4,000 posterior samples. Bolded intervals do not contain 0.

Site	Mean	MCSE	Median	95% CI	ESS	$\hat{R}$
Hillsville	-1.044×10^{-2}	1.843×10^{-4}	-1.008×10^{-2}	$(-2.862 \times 10^{-2}, 6.285 \times 10^{-3})$	2.477×10^{3}	1.001
Hollister	-2.681×10^{-2}	4.543×10^{-4}	-2.673×10^{-2}	$(-6.722 \times 10^{-2}, 1.204 \times 10^{-2})$	2.073×10^{3}	1.001
Hollywood Road	2.327×10^{-2}	6.194×10^{-4}	2.096×10^{-2}	$(-3.956 \times 10^{-2}, 9.278 \times 10^{-2})$	2.894×10^{3}	1.000
Hyperion	-4.860×10^{-4}	2.999×10^{-4}	-5.100×10^{-4}	$(-3.374 \times 10^{-2}, 3.319 \times 10^{-2})$	2.978×10^{3}	1.000
JB Latham	-2.096×10^{-2}	5.112×10^{-4}	-1.980×10^{-2}	$(-6.835 \times 10^{-2}, 2.105 \times 10^{-2})$	2.053×10^{3}	1.001
Jackson	-1.000×10^{-3}	1.734×10^{-4}	-6.494×10^{-4}	$(-1.986 \times 10^{-2}, 1.756 \times 10^{-2})$	2.840×10^{3}	1.000
Jeffersonville	-7.649×10^{-2}	3.702×10^{-4}	-7.739×10^{-2}	$\mathbf{(-0.115, -3.331 \times 10^{-2})}$	3.180×10^{3}	1.000
John M. Asplund	7.545×10^{-3}	8.932×10^{-4}	3.623×10^{-3}	$(-8.253 \times 10^{-2}, 0.108)$	2.686×10^{3}	1.001
Johnnie Mosley	-4.262×10^{-2}	5.088×10^{-4}	-4.290×10^{-2}	$(-8.602 \times 10^{-2}, 5.452 \times 10^{-4})$	2.075×10^{3}	1.001
Johns Creek	-3.364×10^{-2}	6.552×10^{-4}	-3.164×10^{-2}	$(-9.900 \times 10^{-2}, 1.858 \times 10^{-2})$	2.126×10^{3}	1.001
Joint	3.397×10^{-3}	2.189×10^{-4}	2.691×10^{-3}	$(-2.007 \times 10^{-2}, 2.821 \times 10^{-2})$	3.032×10^{3}	1.000
Kansas City	-1.036×10^{-2}	3.083×10^{-4}	-9.482×10^{-3}	$(-4.539 \times 10^{-2}, 2.277 \times 10^{-2})$	3.079×10^{3}	1.000
Kaw Point	-2.822×10^{-2}	3.906×10^{-4}	-2.839×10^{-2}	$(-6.264 \times 10^{-2}, 6.075 \times 10^{-3})$	2.067×10^{3}	1.002
Lancaster	-2.156×10^{-2}	5.545×10^{-4}	-2.031×10^{-2}	$(-7.352 \times 10^{-2}, 2.390 \times 10^{-2})$	1.993×10^{3}	1.000
Lander Street	-1.959×10^{-2}	5.360×10^{-4}	-1.931×10^{-2}	$(-6.399 \times 10^{-2}, 2.345 \times 10^{-2})$	1.799×10^{3}	1.001
Las Gallinas	-2.112×10^{-2}	4.859×10^{-4}	-2.004×10^{-2}	$(-6.213 \times 10^{-2}, 1.581 \times 10^{-2})$	1.810×10^{3}	1.001
Lawrence Kansas	1.128×10^{-2}	2.458×10^{-4}	1.103×10^{-2}	$(-1.156 \times 10^{-2}, 3.525 \times 10^{-2})$	2.332×10^{3}	1.001
Little Falls Run	-1.679×10^{-2}	2.040×10^{-4}	-1.668×10^{-2}	$(-3.675 \times 10^{-2}, 3.228 \times 10^{-3})$	2.532×10^{3}	1.001
Little River	1.350×10^{-3}	5.129×10^{-4}	3.146×10^{-4}	$(-4.804 \times 10^{-2}, 5.440 \times 10^{-2})$	2.348×10^{3}	1.000
Lompoc	-2.226×10^{-4}	3.152×10^{-4}	-4.215×10^{-4}	$(-3.357 \times 10^{-2}, 3.471 \times 10^{-2})$	2.975×10^{3}	1.001
Los Banos	-5.217×10^{-2}	4.792×10^{-4}	-5.207×10^{-2}	$\mathbf{(-0.102, -2.244 \times 10^{-3})}$	2.818×10^{3}	1.000
Loxahatchee	6.459×10^{-3}	2.859×10^{-4}	5.569×10^{-3}	$(-2.246 \times 10^{-2}, 3.663 \times 10^{-2})$	2.732×10^{3}	1.001
MDWASD Central	3.062×10^{-2}	5.223×10^{-4}	3.091×10^{-2}	$(-1.629 \times 10^{-2}, 7.895 \times 10^{-2})$	2.297×10^{3}	1.001
MDWASD North	7.049×10^{-4}	4.777×10^{-4}	7.692×10^{-4}	$(-4.827 \times 10^{-2}, 5.092 \times 10^{-2})$	2.616×10^{3}	1.002
MDWASD South	3.531×10^{-3}	3.547×10^{-4}	2.981×10^{-3}	$(-3.484 \times 10^{-2}, 4.146 \times 10^{-2})$	2.817×10^{3}	1.002
Madera	1.465×10^{-2}	3.760×10^{-4}	1.465×10^{-2}	$(-1.827 \times 10^{-2}, 4.716 \times 10^{-2})$	1.990×10^{3}	1.002
Mankato	3.843×10^{-3}	1.417×10^{-4}	3.082×10^{-3}	$(-1.168 \times 10^{-2}, 2.098 \times 10^{-2})$	3.327×10^{3}	1.000
Markshalltown	2.746×10^{-2}	6.813×10^{-4}	2.524×10^{-2}	$(-3.023 \times 10^{-2}, 9.271 \times 10^{-2})$	2.247×10^{3}	1.000
Marlay Taylor	-8.161×10^{-4}	3.045×10^{-4}	-4.858×10^{-4}	$(-3.539 \times 10^{-2}, 3.284 \times 10^{-2})$	3.110×10^{3}	1.002
Merced	-2.249×10^{-3}	1.893×10^{-4}	-2.025×10^{-3}	$(-2.134 \times 10^{-2}, 1.747 \times 10^{-2})$	2.596×10^{3}	1.001
Mid-Coastside	-3.151×10^{-2}	3.407×10^{-4}	-3.178×10^{-2}	$(-6.500 \times 10^{-2}, 1.558 \times 10^{-3})$	2.669×10^{3}	1.000
Modesto's Sutter	-4.682×10^{-3}	1.537×10^{-4}	-4.084×10^{-3}	$(-1.965 \times 10^{-2}, 9.551 \times 10^{-3})$	2.320×10^{3}	1.000
Montpelier	-2.555×10^{-2}	7.136×10^{-4}	-2.354×10^{-2}	$(-8.948 \times 10^{-2}, 3.305 \times 10^{-2})$	1.935×10^{3}	1.000
Monterey One	-2.854×10^{-2}	5.437×10^{-4}	-2.703×10^{-2}	$(-8.232 \times 10^{-2}, 1.676 \times 10^{-2})$	2.213×10^{3}	1.001
Morris Forman	-2.647×10^{-2}	5.377×10^{-4}	-2.612×10^{-2}	$(-7.388 \times 10^{-2}, 1.632 \times 10^{-2})$	1.886×10^{3}	1.000
Mt. Pleasant	-2.509×10^{-2}	5.529×10^{-4}	-2.393×10^{-2}	$(-8.174 \times 10^{-2}, 2.734 \times 10^{-2})$	2.622×10^{3}	1.000
Muscatine	-1.953×10^{-2}	5.159×10^{-4}	-1.770×10^{-2}	$(-7.564 \times 10^{-2}, 2.687 \times 10^{-2})$	2.521×10^{3}	1.000
Norhtwest Water	-6.851×10^{-2}	2.325×10^{-4}	-6.801×10^{-2}	$\mathbf{(-9.830 \times 10^{-2}, -3.948 \times 10^{-2})}$	4.092×10^{3}	1.000
North Water	1.572×10^{-3}	3.059×10^{-4}	3.598×10^{-4}	$(-2.802 \times 10^{-2}, 3.582 \times 10^{-2})$	2.605×10^{3}	1.000
Novato	-4.129×10^{-2}	2.388×10^{-4}	-4.101×10^{-2}	$\mathbf{(-6.918 \times 10^{-2}, -1.438 \times 10^{-2})}$	3.384×10^{3}	1.000
Ocean	-6.848×10^{-2}	1.061×10^{-2}	-7.389×10^{-2}	$(-0.944, 0.796)$	1.575×10^{3}	1.002
Oceanside	-3.017×10^{-3}	1.183×10^{-4}	-2.691×10^{-3}	$(-1.552 \times 10^{-2}, 8.632 \times 10^{-3})$	2.660×10^{3}	0.999
Ottumwa	-3.264×10^{-2}	3.828×10^{-4}	-3.263×10^{-2}	$(-7.182 \times 10^{-2}, 4.447 \times 10^{-3})$	2.662×10^{3}	1.001
Palo Alto	-1.237×10^{-2}	8.597×10^{-5}	-1.232×10^{-2}	$\mathbf{(-2.299 \times 10^{-2}, -2.271 \times 10^{-3})}$	3.775×10^{3}	1.000
Parker North	-3.255×10^{-2}	5.092×10^{-4}	-3.329×10^{-2}	$(-7.579 \times 10^{-2}, 9.448 \times 10^{-3})$	1.862×10^{3}	1.000
Parker South	9.482×10^{-3}	4.946×10^{-4}	8.463×10^{-3}	$(-3.644 \times 10^{-2}, 5.455 \times 10^{-2})$	2.141×10^{3}	1.000
Paso Robles	-5.197×10^{-3}	3.100×10^{-4}	-4.078×10^{-3}	$(-3.370 \times 10^{-2}, 2.031 \times 10^{-2})$	1.948×10^{3}	1.002
Passaic Valley	3.762×10^{-2}	3.290×10^{-4}	3.789×10^{-2}	$\mathbf{(2.146 \times 10^{-3}, 7.272 \times 10^{-2})}$	2.955×10^{3}	1.000
Penacook	-4.363×10^{-2}	3.539×10^{-4}	-4.419×10^{-2}	$\mathbf{(-7.886 \times 10^{-2}, -6.761 \times 10^{-3})}$	2.775×10^{3}	0.999
Portland	2.866×10^{-3}	2.715×10^{-4}	2.224×10^{-3}	$(-2.804 \times 10^{-2}, 3.314 \times 10^{-2})$	3.107×10^{3}	1.000

Table S8: Mean, Monte Carlo standard error, median, 95% credible interval, effective sample size, and $\hat{R}$ for coefficient on $\psi_7^{\cos}$ (weekly basis function) calculated over 4,000 posterior samples. Bolded intervals do not contain 0.

Site	Mean	MCSE	Median	95% CI	ESS	$\hat{R}$
Provo City	6.631×10^{-3}	5.455×10^{-4}	5.332×10^{-3}	$(-4.709 \times 10^{-2}, 5.938 \times 10^{-2})$	2.406×10^{3}	1.001
RM Clayton	-5.433×10^{-4}	3.439×10^{-4}	-6.220×10^{-5}	$(-4.153 \times 10^{-2}, 3.896 \times 10^{-2})$	3.334×10^{3}	1.000
Red Wing	-2.550×10^{-2}	4.848×10^{-4}	-2.588×10^{-2}	$(-6.558 \times 10^{-2}, 1.758 \times 10^{-2})$	1.955×10^{3}	1.000
Regional	-2.487×10^{-2}	3.946×10^{-4}	-2.412×10^{-2}	$(-6.516 \times 10^{-2}, 1.169 \times 10^{-2})$	2.600×10^{3}	1.000
Regional No. 1	-1.571×10^{-2}	3.619×10^{-4}	-1.490×10^{-2}	$(-5.180 \times 10^{-2}, 1.616 \times 10^{-2})$	2.355×10^{3}	1.000
River Road	5.115×10^{-2}	6.820×10^{-4}	5.087×10^{-2}	$(-1.045 \times 10^{-2}, 0.120)$	2.499×10^{3}	1.001
Riverside	-9.307×10^{-2}	5.165×10^{-4}	-9.307×10^{-2}	$(\mathbf{-0.148}, \mathbf{-3.471 \times 10^{-2}})$	2.987×10^{3}	0.999
Rochester	-2.377×10^{-3}	2.321×10^{-4}	-1.671×10^{-3}	$(-2.654 \times 10^{-2}, 2.111 \times 10^{-2})$	2.660×10^{3}	1.002
SJRA No. 1	2.173×10^{-3}	4.070×10^{-4}	1.593×10^{-3}	$(-4.169 \times 10^{-2}, 4.303 \times 10^{-2})$	2.596×10^{3}	1.001
SJRA No. 2	1.687×10^{-2}	4.027×10^{-4}	1.517×10^{-2}	$(-2.600 \times 10^{-2}, 6.634 \times 10^{-2})$	3.349×10^{3}	1.000
SJRA No. 3	-1.323×10^{-2}	4.576×10^{-4}	-1.206×10^{-2}	$(-6.676 \times 10^{-2}, 4.057 \times 10^{-2})$	3.357×10^{3}	0.999
Sacramento	-2.108×10^{-2}	6.077×10^{-5}	-2.106×10^{-2}	$(\mathbf{-2.808 \times 10^{-2}}, \mathbf{-1.409 \times 10^{-2}})$	3.412×10^{3}	1.000
Salina	-2.005×10^{-2}	3.527×10^{-4}	-2.015×10^{-2}	$(-4.652 \times 10^{-2}, 5.076 \times 10^{-3})$	1.472×10^{3}	1.001
San Francisco	-9.895×10^{-3}	1.277×10^{-4}	-9.744×10^{-3}	$(-2.343 \times 10^{-2}, 1.914 \times 10^{-3})$	2.660×10^{3}	1.000
San Jose-Santa Clara	-1.713×10^{-2}	7.678×10^{-5}	-1.706×10^{-2}	$(\mathbf{-2.678 \times 10^{-2}}, \mathbf{-7.819 \times 10^{-3}})$	4.018×10^{3}	1.000
San Leandro	-4.927×10^{-2}	6.212×10^{-4}	-4.899×10^{-2}	$(-0.112, 6.274 \times 10^{-3})$	2.456×10^{3}	1.000
San Mateo & Estero	-3.566×10^{-3}	2.827×10^{-4}	-2.675×10^{-3}	$(-3.694 \times 10^{-2}, 2.667 \times 10^{-2})$	3.104×10^{3}	1.000
Santa Cruz (City)	1.901×10^{-2}	2.911×10^{-4}	1.863×10^{-2}	$(-7.454 \times 10^{-3}, 4.837 \times 10^{-2})$	2.522×10^{3}	1.001
Santa Cruz (County)	-1.684×10^{-2}	2.993×10^{-4}	-1.638×10^{-2}	$(-4.570 \times 10^{-2}, 8.532 \times 10^{-3})$	2.233×10^{3}	1.000
Santa Rosa	-1.818×10^{-2}	2.883×10^{-4}	-1.787×10^{-2}	$(-4.372 \times 10^{-2}, 4.761 \times 10^{-3})$	1.933×10^{3}	1.000
Sausalito-Marin	-2.311×10^{-3}	4.104×10^{-4}	-9.514×10^{-4}	$(-4.691 \times 10^{-2}, 3.929 \times 10^{-2})$	2.638×10^{3}	1.000
Seaford	1.929×10^{-2}	3.360×10^{-4}	1.920×10^{-2}	$(-1.191 \times 10^{-2}, 5.069 \times 10^{-2})$	2.315×10^{3}	1.001
Silicon Valley	-2.236×10^{-3}	1.185×10^{-4}	-1.727×10^{-3}	$(-1.615 \times 10^{-2}, 1.037 \times 10^{-2})$	3.190×10^{3}	1.000
Somerset Raritan	2.846×10^{-2}	8.999×10^{-4}	2.272×10^{-2}	$(-5.087 \times 10^{-2}, 0.124)$	2.498×10^{3}	0.999
Soscol	-5.098×10^{-3}	2.741×10^{-4}	-4.309×10^{-3}	$(-2.961 \times 10^{-2}, 2.057 \times 10^{-2})$	2.153×10^{3}	1.001
South Bay	-6.083×10^{-3}	4.776×10^{-4}	-4.949×10^{-3}	$(-5.394 \times 10^{-2}, 4.408 \times 10^{-2})$	2.688×10^{3}	1.001
South Bend	-8.638×10^{-5}	3.756×10^{-4}	-3.021×10^{-4}	$(-4.014 \times 10^{-2}, 4.058 \times 10^{-2})$	2.788×10^{3}	1.001
South Burlington	-5.351×10^{-2}	5.165×10^{-4}	-5.383×10^{-2}	$(\mathbf{-0.103}, \mathbf{-2.811 \times 10^{-3}})$	2.508×10^{3}	1.000
South Columbus	-2.956×10^{-3}	2.424×10^{-4}	-2.513×10^{-3}	$(-2.830 \times 10^{-2}, 2.202 \times 10^{-2})$	2.667×10^{3}	1.000
South County	-3.060×10^{-2}	1.309×10^{-4}	-3.058×10^{-2}	$(\mathbf{-4.707 \times 10^{-2}}, \mathbf{-1.440 \times 10^{-2}})$	4.073×10^{3}	0.999
South Laredo	-8.132×10^{-2}	4.736×10^{-4}	-8.266×10^{-2}	$(\mathbf{-0.127}, \mathbf{-2.845 \times 10^{-2}})$	2.796×10^{3}	1.000
South Monmouth	-2.708×10^{-2}	4.246×10^{-4}	-2.678×10^{-2}	$(-6.580 \times 10^{-2}, 1.008 \times 10^{-2})$	2.230×10^{3}	1.001
South River	5.993×10^{-3}	3.670×10^{-4}	4.575×10^{-3}	$(-2.999 \times 10^{-2}, 4.480 \times 10^{-2})$	2.674×10^{3}	1.000
South Water	-1.600×10^{-2}	3.219×10^{-4}	-1.551×10^{-2}	$(-4.549 \times 10^{-2}, 1.100 \times 10^{-2})$	2.159×10^{3}	1.002
Southern Marin	-2.102×10^{-2}	2.981×10^{-4}	-2.084×10^{-2}	$(-5.071 \times 10^{-2}, 6.335 \times 10^{-3})$	2.545×10^{3}	1.000
St. Cloud	-8.216×10^{-3}	4.300×10^{-4}	-6.363×10^{-3}	$(-5.518 \times 10^{-2}, 3.565 \times 10^{-2})$	2.900×10^{3}	1.001
Sunnyvale	-1.003×10^{-2}	8.858×10^{-5}	-9.914×10^{-3}	$(\mathbf{-2.029 \times 10^{-2}}, \mathbf{-5.462 \times 10^{-4}})$	3.231×10^{3}	1.000
Traverse City	-4.069×10^{-2}	8.365×10^{-4}	-3.850×10^{-2}	$(-0.118, 2.747 \times 10^{-2})$	2.063×10^{3}	1.000
Turkey Creek	-1.217×10^{-2}	3.261×10^{-4}	-1.110×10^{-2}	$(-4.850 \times 10^{-2}, 2.123 \times 10^{-2})$	2.924×10^{3}	1.000
Turlock	-4.753×10^{-3}	4.427×10^{-4}	-3.338×10^{-3}	$(-5.402 \times 10^{-2}, 4.443 \times 10^{-2})$	2.929×10^{3}	1.000
Upper Blackstone	-3.691×10^{-2}	6.897×10^{-4}	-3.794×10^{-2}	$(-9.384 \times 10^{-2}, 2.333 \times 10^{-2})$	1.992×10^{3}	1.000
Utoy Creek	1.673×10^{-2}	3.998×10^{-4}	1.572×10^{-2}	$(-1.672 \times 10^{-2}, 5.510 \times 10^{-2})$	2.103×10^{3}	1.002
Vallejo	-1.362×10^{-2}	4.653×10^{-4}	-1.087×10^{-2}	$(-6.470 \times 10^{-2}, 2.914 \times 10^{-2})$	2.632×10^{3}	1.001
Valley	-1.590×10^{-2}	2.794×10^{-4}	-1.592×10^{-2}	$(-4.322 \times 10^{-2}, 9.708 \times 10^{-3})$	2.424×10^{3}	1.001
Valley Creek	-1.118×10^{-2}	3.412×10^{-4}	-9.966×10^{-3}	$(-4.601 \times 10^{-2}, 1.990 \times 10^{-2})$	2.434×10^{3}	1.002
Village Creek	-6.726×10^{-3}	3.671×10^{-4}	-5.765×10^{-3}	$(-4.340 \times 10^{-2}, 3.068 \times 10^{-2})$	2.568×10^{3}	1.000
Warren	2.170×10^{-2}	5.195×10^{-4}	1.925×10^{-2}	$(-2.070 \times 10^{-2}, 7.449 \times 10^{-2})$	2.252×10^{3}	1.000
Weaton	-1.418×10^{-2}	2.799×10^{-4}	-1.375×10^{-2}	$(-3.953 \times 10^{-2}, 8.672 \times 10^{-3})$	1.914×10^{3}	1.001
West Boise	-1.990×10^{-2}	3.940×10^{-4}	-1.896×10^{-2}	$(-6.238 \times 10^{-2}, 1.880 \times 10^{-2})$	2.804×10^{3}	1.000
West County	-2.963×10^{-2}	4.780×10^{-4}	-2.877×10^{-2}	$(-7.725 \times 10^{-2}, 1.056 \times 10^{-2})$	2.288×10^{3}	1.001

Table S8: Mean, Monte Carlo standard error, median, 95% credible interval, effective sample size, and $\hat{R}$ for coefficient on $\psi_7^{\cos}$ (weekly basis function) calculated over 4,000 posterior samples. Bolded intervals do not contain 0.

Site	Mean	MCSE	Median	95% CI	ESS	$\hat{R}$
Wheeling	1.379×10^{-2}	3.746×10^{-4}	1.322×10^{-2}	$(-1.711 \times 10^{-2}, 4.866 \times 10^{-2})$	1.966×10^{3}	1.005
Wichita Falls	-1.497×10^{-3}	2.942×10^{-4}	-1.129×10^{-3}	$(-2.923 \times 10^{-2}, 2.552 \times 10^{-2})$	2.011×10^{3}	1.001
Windsor	-1.213×10^{-2}	5.162×10^{-4}	-9.063×10^{-3}	$(-7.116 \times 10^{-2}, 3.960 \times 10^{-2})$	2.940×10^{3}	1.001
Winters	-5.153×10^{-2}	5.609×10^{-4}	-5.194×10^{-2}	$(-0.103, 8.324 \times 10^{-4})$	2.322×10^{3}	1.003
Wolcott	1.604×10^{-2}	4.716×10^{-4}	1.392×10^{-2}	$(-3.298 \times 10^{-2}, 7.076 \times 10^{-2})$	3.025×10^{3}	1.000
Woodland	-1.385×10^{-2}	4.374×10^{-4}	-1.187×10^{-2}	$(-6.153 \times 10^{-2}, 2.741 \times 10^{-2})$	2.733×10^{3}	1.000
Yankton	-1.623×10^{-2}	4.871×10^{-4}	-1.454×10^{-2}	$(-6.695 \times 10^{-2}, 2.981 \times 10^{-2})$	2.499×10^{3}	1.000
York	-3.660×10^{-2}	5.738×10^{-4}	-3.662×10^{-2}	$(-9.541 \times 10^{-2}, 1.904 \times 10^{-2})$	2.720×10^{3}	1.000
Youngstown	-2.903×10^{-3}	2.895×10^{-4}	-2.798×10^{-3}	$(-3.274 \times 10^{-2}, 3.025 \times 10^{-2})$	2.861×10^{3}	1.002
Zacate Creek	-1.549×10^{-3}	4.339×10^{-4}	-1.120×10^{-3}	$(-5.192 \times 10^{-2}, 4.583 \times 10^{-2})$	2.950×10^{3}	1.000

Table S9: Mean, Monte Carlo standard error, median, 95% credible interval, effective sample size, and $\hat{R}$ for coefficient on $\psi_{365.25}^{\sin}$ (yearly basis function) calculated over 4,000 posterior samples. Bolded intervals do not contain 0.

Site	Mean	MCSE	Median	95% CI	ESS	$\hat{R}$
Akron	-1.503×10^{-2}	4.690×10^{-4}	-1.377×10^{-2}	$(-6.075 \times 10^{-2}, 2.748 \times 10^{-2})$	2.240×10^{3}	1.000
Altamonte Springs	-4.926×10^{-2}	2.951×10^{-4}	-4.964×10^{-2}	$(\mathbf{-7.947 \times 10^{-2}, -1.724 \times 10^{-2}})$	2.867×10^{3}	1.001
Ann Arbor	-1.395×10^{-2}	3.048×10^{-4}	-1.323×10^{-2}	$(-4.809 \times 10^{-2}, 1.855 \times 10^{-2})$	3.013×10^{3}	0.999
Aquia	-4.564×10^{-2}	1.887×10^{-4}	-4.610×10^{-2}	$(\mathbf{-6.867 \times 10^{-2}, -2.092 \times 10^{-2}})$	4.134×10^{3}	1.000
Archie Elledge	-4.973×10^{-2}	4.214×10^{-4}	-4.885×10^{-2}	$(\mathbf{-9.789 \times 10^{-2}, -4.737 \times 10^{-3}})$	3.139×10^{3}	1.001
Bangor	0.115	3.116×10^{-3}	9.789×10^{-2}	$(-0.143, 0.439)$	2.254×10^{3}	1.000
Bayshore	-0.125	1.056×10^{-3}	-0.126	$(\mathbf{-0.216, -2.300 \times 10^{-2}})$	2.111×10^{3}	1.002
Big Creek	2.377×10^{-2}	3.263×10^{-4}	2.332×10^{-2}	$(-8.908 \times 10^{-3}, 6.020 \times 10^{-2})$	3.002×10^{3}	0.999
Boege Alvarado (Fremont)	-1.459×10^{-2}	4.787×10^{-4}	-1.241×10^{-2}	$(-6.709 \times 10^{-2}, 3.604 \times 10^{-2})$	2.828×10^{3}	1.000
Boege Alvarado (Newark)	-3.699×10^{-2}	6.581×10^{-4}	-3.534×10^{-2}	$(-9.811 \times 10^{-2}, 1.447 \times 10^{-2})$	2.113×10^{3}	1.001
Boege Alvarado (Union City)	-3.788×10^{-2}	5.523×10^{-4}	-3.714×10^{-2}	$(-8.899 \times 10^{-2}, 7.777 \times 10^{-3})$	2.014×10^{3}	1.000
Brunswick	-5.536×10^{-2}	3.008×10^{-4}	-5.520×10^{-2}	$(\mathbf{-8.950 \times 10^{-2}, -2.109 \times 10^{-2}})$	3.418×10^{3}	0.999
CODIGA	-4.250×10^{-2}	3.338×10^{-4}	-4.266×10^{-2}	$(\mathbf{-7.606 \times 10^{-2}, -8.193 \times 10^{-3}})$	2.743×10^{3}	1.000
Cahaba River	-9.861×10^{-3}	3.572×10^{-4}	-8.311×10^{-3}	$(-4.464 \times 10^{-2}, 2.098 \times 10^{-2})$	2.126×10^{3}	1.001
Calera Creek	-4.701×10^{-2}	4.139×10^{-4}	-4.711×10^{-2}	$(\mathbf{-8.722 \times 10^{-2}, -4.150 \times 10^{-3}})$	2.621×10^{3}	1.001
Camp Creek	-5.998×10^{-3}	3.306×10^{-4}	-4.459×10^{-3}	$(-4.406 \times 10^{-2}, 3.176 \times 10^{-2})$	3.212×10^{3}	1.000
Capital Region	-3.216×10^{-2}	3.521×10^{-4}	-3.188×10^{-2}	$(-7.029 \times 10^{-2}, 2.724 \times 10^{-3})$	2.834×10^{3}	1.000
Carmel	3.504×10^{-2}	8.414×10^{-4}	3.388×10^{-2}	$(-2.895 \times 10^{-2}, 0.107)$	1.761×10^{3}	1.001
Central Contra Costa	1.459×10^{-2}	2.512×10^{-4}	1.417×10^{-2}	$(-6.144 \times 10^{-3}, 3.856 \times 10^{-2})$	2.160×10^{3}	1.002
Central Marin	-4.682×10^{-2}	6.151×10^{-4}	-4.653×10^{-2}	$(-0.102, 5.602 \times 10^{-3})$	2.008×10^{3}	1.001
Central Marin (W Railroad)	-5.933×10^{-2}	8.096×10^{-4}	-6.011×10^{-2}	$(-0.124, 3.606 \times 10^{-3})$	1.632×10^{3}	1.000
Central Valley	1.956×10^{-3}	2.675×10^{-4}	1.270×10^{-3}	$(-2.206 \times 10^{-2}, 2.726 \times 10^{-2})$	2.092×10^{3}	1.002
Clark County	-7.358×10^{-2}	7.973×10^{-4}	-7.436×10^{-2}	$(\mathbf{-0.133, -8.715 \times 10^{-3}})$	1.570×10^{3}	1.001
Clinton	-0.120	1.956×10^{-4}	-0.120	$(\mathbf{-0.141, -9.889 \times 10^{-2}})$	3.003×10^{3}	0.999
Coastal	-4.273×10^{-3}	5.127×10^{-4}	-2.559×10^{-3}	$(-5.606 \times 10^{-2}, 4.007 \times 10^{-2})$	2.154×10^{3}	1.001
Coeur d'Alene	1.702×10^{-2}	2.650×10^{-4}	1.677×10^{-2}	$(-4.153 \times 10^{-3}, 3.982 \times 10^{-2})$	1.910×10^{3}	1.001
Coralville	-5.453×10^{-2}	5.191×10^{-4}	-5.561×10^{-2}	$(\mathbf{-0.104, -3.447 \times 10^{-4}})$	2.527×10^{3}	1.000
Cumberland	-6.349×10^{-3}	4.849×10^{-4}	-4.636×10^{-3}	$(-6.408 \times 10^{-2}, 5.085 \times 10^{-2})$	3.362×10^{3}	0.999
DELCORA	-1.093×10^{-2}	3.282×10^{-4}	-9.796×10^{-3}	$(-4.802 \times 10^{-2}, 2.468 \times 10^{-2})$	3.198×10^{3}	1.000
Davis	-7.328×10^{-3}	1.295×10^{-4}	-7.041×10^{-3}	$(-2.023 \times 10^{-2}, 3.309 \times 10^{-3})$	2.239×10^{3}	1.001
Deer Island	-3.650×10^{-2}	3.992×10^{-4}	-3.572×10^{-2}	$(\mathbf{-7.483 \times 10^{-2}, -8.002 \times 10^{-5}})$	2.365×10^{3}	1.000
Dillman Road	-4.806×10^{-2}	3.896×10^{-4}	-4.800×10^{-2}	$(\mathbf{-8.499 \times 10^{-2}, -8.580 \times 10^{-3}})$	2.541×10^{3}	1.000
Dover	-3.626×10^{-2}	4.289×10^{-4}	-3.619×10^{-2}	$(-7.726 \times 10^{-2}, 4.166 \times 10^{-3})$	2.410×10^{3}	1.000

Table S9: Mean, Monte Carlo standard error, median, 95% credible interval, effective sample size, and $\hat{R}$ for coefficient on $\psi^{\sin}_{365.25}$ (yearly basis function) calculated over 4,000 posterior samples. Bolded intervals do not contain 0.

Site	Mean	MCSE	Median	95% CI	ESS	$\hat{R}$
Duck Creek	1.056×10^{-2}	2.443×10^{-4}	1.021×10^{-2}	$(-1.106 \times 10^{-2}, 3.421 \times 10^{-2})$	2.304×10^{3}	1.001
E.W. Blom Point Loma	2.303×10^{-2}	4.450×10^{-4}	2.257×10^{-2}	$(-1.087 \times 10^{-2}, 6.220 \times 10^{-2})$	1.803×10^{3}	1.002
East Bay	6.108×10^{-2}	2.885×10^{-4}	6.160×10^{-2}	$(\mathbf{2.719 \times 10^{-2}}, \mathbf{9.343 \times 10^{-2}})$	3.376×10^{3}	1.001
Eastern	1.954×10^{-3}	2.228×10^{-4}	1.199×10^{-3}	$(-2.380 \times 10^{-2}, 2.944 \times 10^{-2})$	3.473×10^{3}	1.000
Ellis Creek	-6.337×10^{-3}	3.132×10^{-4}	-5.286×10^{-3}	$(-4.102 \times 10^{-2}, 2.663 \times 10^{-2})$	2.777×10^{3}	1.000
Esparto	-9.098×10^{-2}	5.424×10^{-4}	-9.156×10^{-2}	$(\mathbf{-0.150}, \mathbf{-3.045 \times 10^{-2}})$	3.095×10^{3}	1.001
Essex	-4.249×10^{-2}	3.914×10^{-4}	-4.220×10^{-2}	$(\mathbf{-8.117 \times 10^{-2}}, \mathbf{-4.710 \times 10^{-3}})$	2.448×10^{3}	1.002
Fairfield-Suisun	9.919×10^{-3}	2.452×10^{-4}	9.279×10^{-3}	$(-1.301 \times 10^{-2}, 3.474 \times 10^{-2})$	2.537×10^{3}	1.001
Five Mile Creek	8.701×10^{-3}	3.055×10^{-4}	7.480×10^{-3}	$(-2.148 \times 10^{-2}, 4.053 \times 10^{-2})$	2.632×10^{3}	1.002
Gainesville	-2.138×10^{-2}	4.643×10^{-4}	-1.943×10^{-2}	$(-7.442 \times 10^{-2}, 2.221 \times 10^{-2})$	2.946×10^{3}	1.001
Garland Rowlett Creek	8.863×10^{-3}	2.398×10^{-4}	8.442×10^{-3}	$(-1.449 \times 10^{-2}, 3.182 \times 10^{-2})$	2.451×10^{3}	1.000
Glenbard	-7.227×10^{-3}	2.777×10^{-4}	-6.606×10^{-3}	$(-3.285 \times 10^{-2}, 1.687 \times 10^{-2})$	2.061×10^{3}	1.003
Grandville	1.464×10^{-2}	2.892×10^{-4}	1.404×10^{-2}	$(-1.194 \times 10^{-2}, 4.328 \times 10^{-2})$	2.407×10^{3}	1.003
Hagerstown	-5.719×10^{-2}	8.229×10^{-4}	-5.686×10^{-2}	$(-0.126, 8.208 \times 10^{-3})$	1.870×10^{3}	1.002
Hall Street	-2.979×10^{-2}	3.784×10^{-4}	-2.935×10^{-2}	$(-6.802 \times 10^{-2}, 3.947 \times 10^{-3})$	2.395×10^{3}	1.001
Hamlin	-1.590×10^{-2}	4.889×10^{-4}	-1.288×10^{-2}	$(-7.027 \times 10^{-2}, 3.274 \times 10^{-2})$	2.721×10^{3}	1.000
Harrison	-2.874×10^{-3}	8.911×10^{-4}	-3.221×10^{-5}	$(-0.103, 8.600 \times 10^{-2})$	2.604×10^{3}	1.000
Hillsville	-5.754×10^{-2}	1.507×10^{-4}	-5.740×10^{-2}	$(\mathbf{-7.694 \times 10^{-2}}, \mathbf{-3.805 \times 10^{-2}})$	4.234×10^{3}	1.001
Hollister	-1.463×10^{-2}	3.873×10^{-4}	-1.282×10^{-2}	$(-5.732 \times 10^{-2}, 2.397 \times 10^{-2})$	2.848×10^{3}	1.000
Hollywood Road	-2.859×10^{-2}	6.873×10^{-4}	-2.746×10^{-2}	$(-9.428 \times 10^{-2}, 3.447 \times 10^{-2})$	2.327×10^{3}	1.000
Hyperion	-3.830×10^{-2}	3.651×10^{-4}	-3.829×10^{-2}	$(\mathbf{-7.867 \times 10^{-2}}, \mathbf{-3.944 \times 10^{-4}})$	3.079×10^{3}	1.001
JB Latham	1.060×10^{-2}	4.304×10^{-4}	9.012×10^{-3}	$(-3.074 \times 10^{-2}, 5.496 \times 10^{-2})$	2.549×10^{3}	1.000
Jackson	3.261×10^{-2}	2.031×10^{-4}	3.261×10^{-2}	$(\mathbf{1.171 \times 10^{-2}}, \mathbf{5.415 \times 10^{-2}})$	2.983×10^{3}	1.001
Jeffersonville	-2.910×10^{-2}	4.423×10^{-4}	-2.851×10^{-2}	$(-7.124 \times 10^{-2}, 8.348 \times 10^{-3})$	2.230×10^{3}	1.001
John M. Asplund	4.952×10^{-2}	2.907×10^{-3}	3.285×10^{-2}	$(-0.159, 0.304)$	1.663×10^{3}	1.000
Johnnie Mosley	-8.474×10^{-3}	3.662×10^{-4}	-7.759×10^{-3}	$(-4.854 \times 10^{-2}, 2.859 \times 10^{-2})$	2.715×10^{3}	1.000
Johns Creek	-1.939×10^{-2}	4.375×10^{-4}	-1.905×10^{-2}	$(-5.582 \times 10^{-2}, 1.445 \times 10^{-2})$	1.764×10^{3}	1.000
Joint	4.395×10^{-2}	1.845×10^{-4}	4.366×10^{-2}	$(\mathbf{2.161 \times 10^{-2}}, \mathbf{6.682 \times 10^{-2}})$	3.994×10^{3}	1.001
Kansas City	-5.996×10^{-2}	4.126×10^{-4}	-5.981×10^{-2}	$(\mathbf{-0.109}, \mathbf{-1.047 \times 10^{-2}})$	3.545×10^{3}	0.999
Kaw Point	6.097×10^{-2}	4.072×10^{-4}	6.056×10^{-2}	$(\mathbf{1.272 \times 10^{-2}}, \mathbf{0.109})$	3.543×10^{3}	0.999
Lancaster	-4.023×10^{-2}	3.969×10^{-4}	-4.052×10^{-2}	$(-7.964 \times 10^{-2}, 1.998 \times 10^{-4})$	2.598×10^{3}	1.002
Lander Street	-7.333×10^{-2}	4.525×10^{-4}	-7.424×10^{-2}	$(\mathbf{-0.124}, \mathbf{-1.971 \times 10^{-2}})$	3.261×10^{3}	1.000
Las Gallinas	-2.643×10^{-2}	4.031×10^{-4}	-2.677×10^{-2}	$(-6.318 \times 10^{-2}, 7.601 \times 10^{-3})$	2.153×10^{3}	1.000
Lawrence Kansas	2.994×10^{-2}	2.739×10^{-4}	2.989×10^{-2}	$(\mathbf{2.700 \times 10^{-3}}, \mathbf{5.554 \times 10^{-2}})$	2.364×10^{3}	1.000
Little Falls Run	-7.766×10^{-2}	1.732×10^{-4}	-7.720×10^{-2}	$(\mathbf{-0.101}, \mathbf{-5.796 \times 10^{-2}})$	3.981×10^{3}	1.000
Little River	7.750×10^{-3}	2.975×10^{-4}	6.297×10^{-3}	$(-2.218 \times 10^{-2}, 4.147 \times 10^{-2})$	2.965×10^{3}	1.000
Lompoc	6.828×10^{-2}	3.830×10^{-4}	6.853×10^{-2}	$(\mathbf{2.636 \times 10^{-2}}, \mathbf{0.110})$	3.104×10^{3}	1.000
Los Banos	-5.859×10^{-2}	4.729×10^{-4}	-5.871×10^{-2}	$(\mathbf{-0.106}, \mathbf{-6.136 \times 10^{-3}})$	2.888×10^{3}	1.001
Loxahatchee	3.702×10^{-3}	2.991×10^{-4}	3.363×10^{-3}	$(-2.499 \times 10^{-2}, 3.268 \times 10^{-2})$	2.346×10^{3}	1.001
MDWASD Central	-1.624×10^{-2}	4.441×10^{-4}	-1.579×10^{-2}	$(-6.045 \times 10^{-2}, 3.014 \times 10^{-2})$	2.612×10^{3}	1.001
MDWASD North	-3.969×10^{-2}	6.714×10^{-4}	-3.932×10^{-2}	$(-9.954 \times 10^{-2}, 2.059 \times 10^{-2})$	2.126×10^{3}	1.000
MDWASD South	-3.234×10^{-2}	4.954×10^{-4}	-3.291×10^{-2}	$(-7.580 \times 10^{-2}, 8.997 \times 10^{-3})$	2.010×10^{3}	1.000
Madera	-7.240×10^{-3}	3.750×10^{-4}	-5.360×10^{-3}	$(-4.437 \times 10^{-2}, 2.584 \times 10^{-2})$	2.256×10^{3}	1.001
Mankato	4.518×10^{-2}	1.803×10^{-4}	4.530×10^{-2}	$(\mathbf{2.579 \times 10^{-2}}, \mathbf{6.411 \times 10^{-2}})$	2.949×10^{3}	1.000
Markshalltown	2.867×10^{-2}	6.538×10^{-4}	2.740×10^{-2}	$(-2.799 \times 10^{-2}, 8.892 \times 10^{-2})$	2.248×10^{3}	1.001
Marlay Taylor	-7.245×10^{-3}	3.792×10^{-4}	-5.758×10^{-3}	$(-4.702 \times 10^{-2}, 3.070 \times 10^{-2})$	2.723×10^{3}	1.000
Merced	1.348×10^{-3}	1.760×10^{-4}	9.381×10^{-4}	$(-1.564 \times 10^{-2}, 1.904 \times 10^{-2})$	2.402×10^{3}	1.000
Mid-Coastside	-3.387×10^{-2}	4.117×10^{-4}	-3.348×10^{-2}	$(-7.205 \times 10^{-2}, 1.637 \times 10^{-3})$	2.148×10^{3}	1.001
Modesto's Sutter	-7.025×10^{-2}	1.428×10^{-4}	-6.974×10^{-2}	$(\mathbf{-8.968 \times 10^{-2}}, \mathbf{-5.392 \times 10^{-2}})$	4.096×10^{3}	1.000
Montpelier	-8.414×10^{-2}	3.885×10^{-4}	-8.444×10^{-2}	$(\mathbf{-0.128}, \mathbf{-4.041 \times 10^{-2}})$	3.365×10^{3}	1.001

Table S9: Mean, Monte Carlo standard error, median, 95% credible interval, effective sample size, and $\hat{R}$ for coefficient on $\psi^{\sin}_{365.25}$ (yearly basis function) calculated over 4,000 posterior samples. Bolded intervals do not contain 0.

Site	Mean	MCSE	Median	95% CI	ESS	$\hat{R}$
Monterey One	1.306×10^{-2}	4.771×10^{-4}	9.204×10^{-3}	$(-3.283 \times 10^{-2}, 7.161 \times 10^{-2})$	2.970×10^{3}	1.000
Morris Forman	-1.127×10^{-2}	3.757×10^{-4}	-1.026×10^{-2}	$(-4.643 \times 10^{-2}, 2.490 \times 10^{-2})$	2.316×10^{3}	1.001
Mt. Pleasant	-2.282×10^{-2}	9.434×10^{-4}	-1.681×10^{-2}	$(-0.115, 5.315 \times 10^{-2})$	2.014×10^{3}	1.005
Muscatine	-6.686×10^{-3}	3.594×10^{-4}	-5.621×10^{-3}	$(-4.482 \times 10^{-2}, 3.001 \times 10^{-2})$	2.606×10^{3}	0.999
Norhtwest Water	-4.222×10^{-2}	2.928×10^{-4}	-4.245×10^{-2}	$\mathbf{(-7.476 \times 10^{-2}, -9.832 \times 10^{-3})}$	3.112×10^{3}	1.000
North Water	-2.843×10^{-3}	2.733×10^{-4}	-2.261×10^{-3}	$(-3.493 \times 10^{-2}, 2.683 \times 10^{-2})$	3.114×10^{3}	1.000
Novato	2.196×10^{-2}	4.013×10^{-4}	2.184×10^{-2}	$(-7.462 \times 10^{-3}, 5.390 \times 10^{-2})$	1.589×10^{3}	1.002
Ocean	-3.209×10^{-3}	9.008×10^{-4}	-2.838×10^{-3}	$(-9.532 \times 10^{-2}, 0.100)$	2.834×10^{3}	1.000
Oceanside	1.810×10^{-2}	1.422×10^{-4}	1.823×10^{-2}	$\mathbf{(5.376 \times 10^{-3}, 3.020 \times 10^{-2})}$	1.922×10^{3}	1.001
Ottumwa	2.654×10^{-2}	3.678×10^{-4}	2.526×10^{-2}	$(-8.027 \times 10^{-3}, 6.799 \times 10^{-2})$	2.845×10^{3}	1.002
Palo Alto	-9.553×10^{-3}	1.002×10^{-4}	-9.519×10^{-3}	$(-2.005 \times 10^{-2}, 6.479 \times 10^{-4})$	2.837×10^{3}	1.000
Parker North	-2.948×10^{-3}	2.921×10^{-4}	-2.259×10^{-3}	$(-3.470 \times 10^{-2}, 2.821 \times 10^{-2})$	2.903×10^{3}	1.001
Parker South	6.280×10^{-3}	2.826×10^{-4}	4.745×10^{-3}	$(-2.314 \times 10^{-2}, 4.094 \times 10^{-2})$	3.199×10^{3}	1.001
Paso Robles	6.074×10^{-2}	1.707×10^{-4}	6.052×10^{-2}	$\mathbf{(4.169 \times 10^{-2}, 8.093 \times 10^{-2})}$	3.441×10^{3}	1.000
Passaic Valley	6.470×10^{-4}	2.597×10^{-4}	5.686×10^{-4}	$(-3.051 \times 10^{-2}, 3.122 \times 10^{-2})$	3.557×10^{3}	1.000
Penacook	1.336×10^{-2}	3.047×10^{-4}	1.222×10^{-2}	$(-1.572 \times 10^{-2}, 4.679 \times 10^{-2})$	2.827×10^{3}	1.001
Portland	-6.118×10^{-3}	4.170×10^{-4}	-4.485×10^{-3}	$(-4.938 \times 10^{-2}, 3.332 \times 10^{-2})$	2.415×10^{3}	1.002
Provo City	1.841×10^{-2}	3.863×10^{-4}	1.799×10^{-2}	$(-1.724 \times 10^{-2}, 5.509 \times 10^{-2})$	2.407×10^{3}	1.002
RM Clayton	-5.726×10^{-2}	4.141×10^{-4}	-5.731×10^{-2}	$\mathbf{(-0.102, -1.266 \times 10^{-2})}$	2.877×10^{3}	1.001
Red Wing	3.512×10^{-2}	1.229×10^{-3}	3.318×10^{-2}	$(-5.113 \times 10^{-2}, 0.128)$	1.378×10^{3}	1.002
Regional	-3.745×10^{-2}	4.885×10^{-4}	-3.727×10^{-2}	$(-8.321 \times 10^{-2}, 7.562 \times 10^{-3})$	2.356×10^{3}	1.003
Regional No. 1	3.752×10^{-2}	2.808×10^{-4}	3.788×10^{-2}	$\mathbf{(6.861 \times 10^{-3}, 6.638 \times 10^{-2})}$	2.913×10^{3}	1.000
River Road	-4.103×10^{-2}	5.168×10^{-4}	-4.151×10^{-2}	$(-9.084 \times 10^{-2}, 5.312 \times 10^{-3})$	2.437×10^{3}	1.000
Riverside	-3.241×10^{-2}	5.504×10^{-4}	-3.167×10^{-2}	$(-8.682 \times 10^{-2}, 1.727 \times 10^{-2})$	2.465×10^{3}	1.001
Rochester	3.386×10^{-2}	2.190×10^{-4}	3.324×10^{-2}	$\mathbf{(9.102 \times 10^{-3}, 6.205 \times 10^{-2})}$	3.844×10^{3}	1.000
SJRA No. 1	-2.365×10^{-2}	5.036×10^{-4}	-2.229×10^{-2}	$(-7.555 \times 10^{-2}, 2.157 \times 10^{-2})$	2.470×10^{3}	1.001
SJRA No. 2	-7.280×10^{-2}	4.847×10^{-4}	-7.168×10^{-2}	$\mathbf{(-0.131, -2.068 \times 10^{-2})}$	3.362×10^{3}	1.000
SJRA No. 3	-6.369×10^{-2}	6.929×10^{-4}	-6.395×10^{-2}	$\mathbf{(-0.126, -7.687 \times 10^{-4})}$	2.134×10^{3}	1.001
Sacramento	-5.095×10^{-3}	7.638×10^{-5}	-4.975×10^{-3}	$(-1.276 \times 10^{-2}, 1.772 \times 10^{-3})$	2.484×10^{3}	1.002
Salina	4.453×10^{-2}	3.957×10^{-4}	4.516×10^{-2}	$\mathbf{(2.980 \times 10^{-3}, 8.551 \times 10^{-2})}$	2.692×10^{3}	1.002
San Francisco	1.272×10^{-2}	1.465×10^{-4}	1.312×10^{-2}	$(-1.116 \times 10^{-3}, 2.589 \times 10^{-2})$	2.330×10^{3}	1.000
San Jose-Santa Clara	-2.434×10^{-3}	9.288×10^{-5}	-2.095×10^{-3}	$(-1.204 \times 10^{-2}, 6.325 \times 10^{-3})$	2.459×10^{3}	1.000
San Leandro	-3.177×10^{-2}	5.694×10^{-4}	-3.059×10^{-2}	$(-8.714 \times 10^{-2}, 1.546 \times 10^{-2})$	2.205×10^{3}	1.001
San Mateo & Estero	1.668×10^{-2}	3.748×10^{-4}	1.588×10^{-2}	$(-1.453 \times 10^{-2}, 5.099 \times 10^{-2})$	2.083×10^{3}	1.001
Santa Cruz (City)	1.447×10^{-2}	3.620×10^{-4}	1.355×10^{-2}	$(-1.299 \times 10^{-2}, 4.559 \times 10^{-2})$	1.739×10^{3}	1.000
Santa Cruz (County)	1.327×10^{-2}	2.976×10^{-4}	1.280×10^{-2}	$(-1.030 \times 10^{-2}, 3.977 \times 10^{-2})$	1.903×10^{3}	1.002
Santa Rosa	-2.345×10^{-2}	2.535×10^{-4}	-2.373×10^{-2}	$\mathbf{(-4.518 \times 10^{-2}, -4.971 \times 10^{-4})}$	1.913×10^{3}	1.000
Sausalito-Marin	1.972×10^{-2}	4.493×10^{-4}	1.905×10^{-2}	$(-1.710 \times 10^{-2}, 6.132 \times 10^{-2})$	1.982×10^{3}	1.000
Seaford	-6.425×10^{-2}	2.596×10^{-4}	-6.502×10^{-2}	$\mathbf{(-9.546 \times 10^{-2}, -3.066 \times 10^{-2})}$	3.858×10^{3}	0.999
Silicon Valley	-4.002×10^{-3}	1.177×10^{-4}	-3.616×10^{-3}	$(-1.788 \times 10^{-2}, 9.015 \times 10^{-3})$	3.403×10^{3}	1.001
Somerset Raritan	-0.176	3.457×10^{-3}	-0.172	$(-0.417, 2.492 \times 10^{-2})$	1.149×10^{3}	1.002
Soscol	2.078×10^{-3}	2.120×10^{-4}	1.107×10^{-3}	$(-1.859 \times 10^{-2}, 2.547 \times 10^{-2})$	2.676×10^{3}	1.002
South Bay	-4.994×10^{-3}	8.219×10^{-4}	-3.172×10^{-3}	$(-9.090 \times 10^{-2}, 7.725 \times 10^{-2})$	2.383×10^{3}	1.001
South Bend	-2.689×10^{-2}	4.531×10^{-4}	-2.727×10^{-2}	$(-6.420 \times 10^{-2}, 1.021 \times 10^{-2})$	1.900×10^{3}	1.001
South Burlington	-4.154×10^{-2}	3.397×10^{-4}	-4.151×10^{-2}	$\mathbf{(-7.779 \times 10^{-2}, -4.329 \times 10^{-3})}$	3.003×10^{3}	1.000
South Columbus	-3.056×10^{-2}	2.918×10^{-4}	-3.045×10^{-2}	$\mathbf{(-5.867 \times 10^{-2}, -9.267 \times 10^{-4})}$	2.530×10^{3}	1.001
South County	1.387×10^{-2}	1.738×10^{-4}	1.392×10^{-2}	$(-1.871 \times 10^{-3}, 3.032 \times 10^{-2})$	2.378×10^{3}	1.000
South Laredo	-9.793×10^{-2}	4.485×10^{-4}	-9.764×10^{-2}	$\mathbf{(-0.153, -4.266 \times 10^{-2})}$	3.882×10^{3}	1.001
South Monmouth	-0.111	3.218×10^{-4}	-0.111	$\mathbf{(-0.151, -7.408 \times 10^{-2})}$	3.702×10^{3}	1.000
South River	-6.173×10^{-3}	3.285×10^{-4}	-4.312×10^{-3}	$(-4.702 \times 10^{-2}, 2.919 \times 10^{-2})$	3.087×10^{3}	1.000

Table S9: Mean, Monte Carlo standard error, median, 95% credible interval, effective sample size, and $\hat{R}$ for coefficient on $\psi^{\sin}_{365.25}$ (yearly basis function) calculated over 4,000 posterior samples. Bolded intervals do not contain 0.

Site	Mean	MCSE	Median	95% CI	ESS	$\hat{R}$
South Water	2.217×10^{-2}	3.739×10^{-4}	2.270×10^{-2}	$(-9.115 \times 10^{-3}, 5.296 \times 10^{-2})$	1.953×10^{3}	1.001
Southern Marin	-3.378×10^{-2}	2.568×10^{-4}	-3.403×10^{-2}	$(\mathbf{-6.094 \times 10^{-2}}, \mathbf{-5.511 \times 10^{-3}})$	2.982×10^{3}	1.001
St. Cloud	-9.850×10^{-3}	5.931×10^{-4}	-7.958×10^{-3}	$(-6.736 \times 10^{-2}, 4.384 \times 10^{-2})$	2.232×10^{3}	1.000
Sunnyvale	7.201×10^{-3}	9.774×10^{-5}	7.146×10^{-3}	$(-1.959 \times 10^{-3}, 1.743 \times 10^{-2})$	2.754×10^{3}	1.001
Traverse City	-3.711×10^{-2}	5.418×10^{-4}	-3.711×10^{-2}	$(-8.654 \times 10^{-2}, 1.065 \times 10^{-2})$	2.252×10^{3}	1.001
Turkey Creek	2.253×10^{-2}	4.260×10^{-4}	2.196×10^{-2}	$(-1.644 \times 10^{-2}, 6.438 \times 10^{-2})$	2.469×10^{3}	1.000
Turlock	-0.108	4.619×10^{-4}	-0.109	$(\mathbf{-0.158}, \mathbf{-5.126 \times 10^{-2}})$	3.552×10^{3}	1.000
Upper Blackstone	-0.127	4.985×10^{-4}	-0.126	$(\mathbf{-0.189}, \mathbf{-7.052 \times 10^{-2}})$	3.711×10^{3}	0.999
Utoy Creek	-2.671×10^{-2}	3.821×10^{-4}	-2.740×10^{-2}	$(-6.292 \times 10^{-2}, 8.376 \times 10^{-3})$	2.367×10^{3}	1.000
Vallejo	2.640×10^{-2}	4.605×10^{-4}	2.507×10^{-2}	$(-1.539 \times 10^{-2}, 7.331 \times 10^{-2})$	2.607×10^{3}	1.001
Valley	-3.894×10^{-2}	2.289×10^{-4}	-3.917×10^{-2}	$(\mathbf{-6.529 \times 10^{-2}}, \mathbf{-1.204 \times 10^{-2}})$	3.481×10^{3}	1.000
Valley Creek	2.807×10^{-2}	4.445×10^{-4}	2.785×10^{-2}	$(-4.730 \times 10^{-3}, 6.312 \times 10^{-2})$	1.604×10^{3}	1.000
Village Creek	1.978×10^{-2}	4.394×10^{-4}	1.897×10^{-2}	$(-1.667 \times 10^{-2}, 6.207 \times 10^{-2})$	2.150×10^{3}	1.000
Warren	-5.429×10^{-2}	3.119×10^{-4}	-5.450×10^{-2}	$(\mathbf{-9.089 \times 10^{-2}}, \mathbf{-1.769 \times 10^{-2}})$	3.524×10^{3}	1.000
Weaton	3.820×10^{-2}	2.559×10^{-4}	3.807×10^{-2}	$(\mathbf{1.196 \times 10^{-2}}, \mathbf{6.346 \times 10^{-2}})$	2.551×10^{3}	1.000
West Boise	-3.096×10^{-2}	6.236×10^{-4}	-3.044×10^{-2}	$(-8.325 \times 10^{-2}, 1.754 \times 10^{-2})$	1.807×10^{3}	1.001
West County	2.864×10^{-2}	4.309×10^{-4}	2.837×10^{-2}	$(-9.226 \times 10^{-3}, 6.696 \times 10^{-2})$	2.050×10^{3}	0.999
Wheeling	-2.408×10^{-2}	3.619×10^{-4}	-2.449×10^{-2}	$(-5.453 \times 10^{-2}, 6.580 \times 10^{-3})$	2.032×10^{3}	1.001
Wichita Falls	-1.355×10^{-2}	3.245×10^{-4}	-1.239×10^{-2}	$(-4.572 \times 10^{-2}, 1.266 \times 10^{-2})$	2.097×10^{3}	1.000
Windsor	-1.451×10^{-2}	6.647×10^{-4}	-1.236×10^{-2}	$(-7.880 \times 10^{-2}, 5.116 \times 10^{-2})$	2.433×10^{3}	1.001
Winters	-5.515×10^{-2}	6.639×10^{-4}	-5.483×10^{-2}	$(-0.120, 5.730 \times 10^{-3})$	2.559×10^{3}	1.000
Wolcott	-2.050×10^{-2}	6.630×10^{-4}	-1.883×10^{-2}	$(-7.618 \times 10^{-2}, 3.023 \times 10^{-2})$	1.674×10^{3}	0.999
Woodland	-1.826×10^{-2}	5.275×10^{-4}	-1.626×10^{-2}	$(-7.596 \times 10^{-2}, 3.089 \times 10^{-2})$	2.581×10^{3}	1.001
Yankton	2.538×10^{-2}	8.384×10^{-4}	2.136×10^{-2}	$(-3.982 \times 10^{-2}, 0.108)$	1.997×10^{3}	1.001
York	-2.755×10^{-2}	4.206×10^{-4}	-2.762×10^{-2}	$(-6.630 \times 10^{-2}, 9.844 \times 10^{-3})$	2.221×10^{3}	1.001
Youngstown	-1.166×10^{-2}	5.533×10^{-4}	-9.937×10^{-3}	$(-5.851 \times 10^{-2}, 3.268 \times 10^{-2})$	1.680×10^{3}	1.002
Zacate Creek	-5.941×10^{-2}	4.565×10^{-4}	-6.040×10^{-2}	$(\mathbf{-0.111}, \mathbf{-5.608 \times 10^{-3}})$	3.422×10^{3}	1.000

Table S10: Mean, Monte Carlo standard error, median, 95% credible interval, effective sample size, and $\hat{R}$ for coefficient on $\psi^{\cos}_{365.25}$ (yearly basis function) calculated over 4,000 posterior samples. Bolded intervals do not contain 0.

Site	Mean	MCSE	Median	95% CI	ESS	$\hat{R}$
Akron	3.438×10^{-2}	4.013×10^{-4}	3.432×10^{-2}	$(-1.282 \times 10^{-3}, 7.545 \times 10^{-2})$	2.470×10^{3}	1.000
Altamonte Springs	-7.636×10^{-3}	2.961×10^{-4}	-6.691×10^{-3}	$(-3.699 \times 10^{-2}, 1.877 \times 10^{-2})$	2.188×10^{3}	1.001
Ann Arbor	-4.687×10^{-2}	4.478×10^{-4}	-4.625×10^{-2}	$(\mathbf{-8.826 \times 10^{-2}}, \mathbf{-7.572 \times 10^{-3}})$	2.085×10^{3}	1.001
Aquia	1.300×10^{-4}	1.955×10^{-4}	1.362×10^{-4}	$(-2.108 \times 10^{-2}, 2.161 \times 10^{-2})$	3.041×10^{3}	1.000
Archie Elledge	1.273×10^{-3}	4.101×10^{-4}	5.586×10^{-4}	$(-4.204 \times 10^{-2}, 4.537 \times 10^{-2})$	2.686×10^{3}	0.999
Bangor	0.389	4.429×10^{-3}	0.386	$(\mathbf{1.794 \times 10^{-2}}, \mathbf{0.772})$	1.809×10^{3}	1.001
Bayshore	-7.737×10^{-2}	9.617×10^{-4}	-7.778×10^{-2}	$(-0.165, 3.147 \times 10^{-3})$	1.991×10^{3}	1.001
Big Creek	-1.479×10^{-2}	3.266×10^{-4}	-1.372×10^{-2}	$(-4.808 \times 10^{-2}, 1.414 \times 10^{-2})$	2.346×10^{3}	1.000
Boege Alvarado (Fremont)	-3.830×10^{-2}	5.423×10^{-4}	-3.788×10^{-2}	$(-9.067 \times 10^{-2}, 9.053 \times 10^{-3})$	2.270×10^{3}	1.002
Boege Alvarado (Newark)	-2.578×10^{-2}	5.489×10^{-4}	-2.452×10^{-2}	$(-8.157 \times 10^{-2}, 2.603 \times 10^{-2})$	2.584×10^{3}	1.000
Boege Alvarado (Union City)	-6.220×10^{-3}	3.424×10^{-4}	-4.302×10^{-3}	$(-5.107 \times 10^{-2}, 3.262 \times 10^{-2})$	3.462×10^{3}	1.000
Brunswick	-1.745×10^{-2}	3.264×10^{-4}	-1.629×10^{-2}	$(-5.343 \times 10^{-2}, 1.375 \times 10^{-2})$	2.940×10^{3}	1.000
CODIGA	-7.949×10^{-2}	2.787×10^{-4}	-7.989×10^{-2}	$(\mathbf{-0.111}, \mathbf{-4.555 \times 10^{-2}})$	3.665×10^{3}	0.999
Cahaba River	-3.849×10^{-2}	3.433×10^{-4}	-3.881×10^{-2}	$(\mathbf{-6.926 \times 10^{-2}}, \mathbf{-5.604 \times 10^{-3}})$	2.105×10^{3}	1.001
Calera Creek	-2.187×10^{-2}	4.805×10^{-4}	-2.087×10^{-2}	$(-6.495 \times 10^{-2}, 1.587 \times 10^{-2})$	1.898×10^{3}	1.002
Camp Creek	-2.293×10^{-2}	4.015×10^{-4}	-2.190×10^{-2}	$(-6.089 \times 10^{-2}, 1.128 \times 10^{-2})$	2.219×10^{3}	1.001

Table S10: Mean, Monte Carlo standard error, median, 95% credible interval, effective sample size, and $\hat{R}$ for coefficient on $\psi^{\cos}_{365.25}$ (yearly basis function) calculated over 4,000 posterior samples. Bolded intervals do not contain 0.

Site	Mean	MCSE	Median	95% CI	ESS	$\hat{R}$
Capital Region	3.189×10^{-4}	2.625×10^{-4}	2.999×10^{-5}	$(-2.999 \times 10^{-2}, 3.017 \times 10^{-2})$	3.109×10^{3}	1.000
Carmel	3.420×10^{-2}	9.641×10^{-4}	3.461×10^{-2}	$(-4.326 \times 10^{-2}, 0.106)$	1.620×10^{3}	1.001
Central Contra Costa	7.505×10^{-3}	2.295×10^{-4}	6.716×10^{-3}	$(-1.228 \times 10^{-2}, 3.017 \times 10^{-2})$	2.185×10^{3}	1.000
Central Marin	-4.250×10^{-3}	4.290×10^{-4}	-2.359×10^{-3}	$(-5.334 \times 10^{-2}, 4.015 \times 10^{-2})$	2.934×10^{3}	1.000
Central Marin (W Railroad)	-4.189×10^{-2}	5.547×10^{-4}	-4.191×10^{-2}	$(-9.651 \times 10^{-2}, 8.633 \times 10^{-3})$	2.509×10^{3}	1.002
Central Valley	-3.828×10^{-2}	2.160×10^{-4}	-3.856×10^{-2}	$\mathbf{(-5.945 \times 10^{-2}, -1.475 \times 10^{-2})}$	2.732×10^{3}	1.000
Clark County	-3.783×10^{-2}	7.344×10^{-4}	-3.796×10^{-2}	$(-9.439 \times 10^{-2}, 1.362 \times 10^{-2})$	1.438×10^{3}	1.001
Clinton	2.067×10^{-2}	2.096×10^{-4}	2.051×10^{-2}	$\mathbf{(4.569 \times 10^{-4}, 4.095 \times 10^{-2})}$	2.522×10^{3}	1.001
Coastal	3.671×10^{-2}	5.212×10^{-4}	3.615×10^{-2}	$(-7.619 \times 10^{-3}, 8.691 \times 10^{-2})$	2.282×10^{3}	0.999
Coeur d'Alene	-6.142×10^{-3}	2.343×10^{-4}	-5.343×10^{-3}	$(-3.071 \times 10^{-2}, 1.650 \times 10^{-2})$	2.577×10^{3}	1.001
Coralville	2.732×10^{-3}	4.832×10^{-4}	1.296×10^{-3}	$(-4.635 \times 10^{-2}, 5.343 \times 10^{-2})$	2.530×10^{3}	1.000
Cumberland	-1.787×10^{-2}	5.978×10^{-4}	-1.548×10^{-2}	$(-7.611 \times 10^{-2}, 3.572 \times 10^{-2})$	2.304×10^{3}	1.001
DELCORA	1.328×10^{-4}	3.704×10^{-4}	2.457×10^{-5}	$(-3.972 \times 10^{-2}, 4.177 \times 10^{-2})$	2.969×10^{3}	1.001
Davis	4.066×10^{-4}	1.148×10^{-4}	3.662×10^{-4}	$(-1.109 \times 10^{-2}, 1.132 \times 10^{-2})$	2.315×10^{3}	1.002
Deer Island	2.180×10^{-2}	3.117×10^{-4}	2.147×10^{-2}	$(-7.062 \times 10^{-3}, 5.314 \times 10^{-2})$	2.556×10^{3}	1.000
Dillman Road	-2.260×10^{-2}	4.626×10^{-4}	-2.246×10^{-2}	$(-6.001 \times 10^{-2}, 1.525 \times 10^{-2})$	1.868×10^{3}	1.000
Dover	-1.940×10^{-2}	4.424×10^{-4}	-1.845×10^{-2}	$(-6.391 \times 10^{-2}, 2.073 \times 10^{-2})$	2.482×10^{3}	0.999
Duck Creek	2.421×10^{-2}	2.024×10^{-4}	2.429×10^{-2}	$\mathbf{(3.097 \times 10^{-3}, 4.497 \times 10^{-2})}$	2.873×10^{3}	1.000
E.W. Blom Point Loma	-5.101×10^{-2}	3.130×10^{-4}	-5.120×10^{-2}	$\mathbf{(-8.691 \times 10^{-2}, -1.473 \times 10^{-2})}$	3.586×10^{3}	1.000
East Bay	-1.606×10^{-3}	2.788×10^{-4}	-9.638×10^{-4}	$(-2.877 \times 10^{-2}, 2.507 \times 10^{-2})$	2.308×10^{3}	1.001
Eastern	-3.720×10^{-2}	4.124×10^{-4}	-3.708×10^{-2}	$\mathbf{(-7.350 \times 10^{-2}, -2.145 \times 10^{-3})}$	1.864×10^{3}	1.003
Ellis Creek	3.335×10^{-2}	3.250×10^{-4}	3.390×10^{-2}	$(-7.594 \times 10^{-5}, 6.502 \times 10^{-2})$	2.699×10^{3}	1.000
Esparto	-2.800×10^{-2}	6.507×10^{-4}	-2.653×10^{-2}	$(-8.975 \times 10^{-2}, 2.785 \times 10^{-2})$	2.242×10^{3}	1.001
Essex	3.487×10^{-2}	4.815×10^{-4}	3.409×10^{-2}	$(-4.601 \times 10^{-3}, 8.249 \times 10^{-2})$	2.150×10^{3}	1.000
Fairfield-Suisun	-1.023×10^{-2}	2.528×10^{-4}	-9.742×10^{-3}	$(-3.542 \times 10^{-2}, 1.371 \times 10^{-2})$	2.474×10^{3}	1.000
Five Mile Creek	-2.836×10^{-3}	2.603×10^{-4}	-2.426×10^{-3}	$(-2.882 \times 10^{-2}, 2.403 \times 10^{-2})$	2.536×10^{3}	1.003
Gainesville	-5.043×10^{-2}	5.456×10^{-4}	-5.203×10^{-2}	$(-0.100, 8.031 \times 10^{-4})$	2.282×10^{3}	1.001
Garland Rowlett Creek	-2.299×10^{-2}	2.292×10^{-4}	-2.328×10^{-2}	$(-4.794 \times 10^{-2}, 8.770 \times 10^{-4})$	2.996×10^{3}	1.000
Glenbard	-3.619×10^{-2}	2.699×10^{-4}	-3.614×10^{-2}	$\mathbf{(-6.511 \times 10^{-2}, -6.851 \times 10^{-3})}$	3.003×10^{3}	1.001
Grandville	1.066×10^{-2}	2.915×10^{-4}	9.408×10^{-3}	$(-1.728 \times 10^{-2}, 4.199 \times 10^{-2})$	2.672×10^{3}	1.000
Hagerstown	-6.366×10^{-2}	6.521×10^{-4}	-6.286×10^{-2}	$(-0.134, 2.241 \times 10^{-4})$	2.838×10^{3}	1.002
Hall Street	-5.766×10^{-3}	2.790×10^{-4}	-4.652×10^{-3}	$(-3.743 \times 10^{-2}, 2.344 \times 10^{-2})$	2.993×10^{3}	1.000
Hamlin	-6.616×10^{-2}	6.367×10^{-4}	-6.686×10^{-2}	$\mathbf{(-0.121, -8.865 \times 10^{-3})}$	2.016×10^{3}	1.001
Harrison	-1.871×10^{-2}	9.484×10^{-4}	-1.242×10^{-2}	$(-0.127, 7.195 \times 10^{-2})$	2.594×10^{3}	1.000
Hillsville	-1.044×10^{-2}	2.047×10^{-4}	-9.936×10^{-3}	$(-3.407 \times 10^{-2}, 1.092 \times 10^{-2})$	3.102×10^{3}	1.000
Hollister	-5.594×10^{-2}	4.664×10^{-4}	-5.521×10^{-2}	$\mathbf{(-0.104, -8.312 \times 10^{-3})}$	2.637×10^{3}	1.000
Hollywood Road	2.602×10^{-2}	6.301×10^{-4}	2.490×10^{-2}	$(-2.768 \times 10^{-2}, 8.679 \times 10^{-2})$	2.142×10^{3}	1.000
Hyperion	-5.986×10^{-2}	3.999×10^{-4}	-6.050×10^{-2}	$\mathbf{(-9.736 \times 10^{-2}, -1.956 \times 10^{-2})}$	2.370×10^{3}	1.000
JB Latham	-7.180×10^{-2}	5.539×10^{-4}	-7.114×10^{-2}	$\mathbf{(-0.134, -1.011 \times 10^{-2})}$	3.463×10^{3}	1.000
Jackson	1.912×10^{-2}	1.972×10^{-4}	1.924×10^{-2}	$\mathbf{(1.398 \times 10^{-4}, 3.783 \times 10^{-2})}$	2.449×10^{3}	1.002
Jeffersonville	-4.537×10^{-2}	4.439×10^{-4}	-4.592×10^{-2}	$\mathbf{(-8.954 \times 10^{-2}, -1.377 \times 10^{-4})}$	2.602×10^{3}	0.999
John M. Asplund	0.141	3.021×10^{-3}	0.131	$(-6.036 \times 10^{-2}, 0.401)$	1.589×10^{3}	1.000
Johnnie Mosley	-1.236×10^{-2}	4.228×10^{-4}	-1.090×10^{-2}	$(-5.025 \times 10^{-2}, 2.284 \times 10^{-2})$	1.922×10^{3}	1.000
Johns Creek	-6.680×10^{-2}	2.948×10^{-4}	-6.639×10^{-2}	$\mathbf{(-0.105, -2.697 \times 10^{-2})}$	4.400×10^{3}	1.001
Joint	-8.857×10^{-3}	2.546×10^{-4}	-7.970×10^{-3}	$(-3.404 \times 10^{-2}, 1.335 \times 10^{-2})$	2.300×10^{3}	1.002
Kansas City	-6.646×10^{-2}	4.181×10^{-4}	-6.615×10^{-2}	$\mathbf{(-0.115, -1.829 \times 10^{-2})}$	3.592×10^{3}	1.000
Kaw Point	-7.805×10^{-2}	4.089×10^{-4}	-7.820×10^{-2}	$\mathbf{(-0.122, -3.226 \times 10^{-2})}$	3.175×10^{3}	1.000
Lancaster	-4.555×10^{-2}	3.641×10^{-4}	-4.496×10^{-2}	$\mathbf{(-8.796 \times 10^{-2}, -4.766 \times 10^{-3})}$	3.372×10^{3}	1.001
Lander Street	5.456×10^{-2}	4.871×10^{-4}	5.491×10^{-2}	$\mathbf{(3.236 \times 10^{-3}, 0.107)}$	2.877×10^{3}	1.000
Las Gallinas	-3.971×10^{-2}	3.403×10^{-4}	-3.941×10^{-2}	$\mathbf{(-7.664 \times 10^{-2}, -4.481 \times 10^{-3})}$	2.905×10^{3}	1.000

Table S10: Mean, Monte Carlo standard error, median, 95% credible interval, effective sample size, and $\hat{R}$ for coefficient on $\psi^{\cos}_{365.25}$ (yearly basis function) calculated over 4,000 posterior samples. Bolded intervals do not contain 0.

Site	Mean	MCSE	Median	95% CI	ESS	$\hat{R}$
Lawrence Kansas	4.735×10^{-2}	2.176×10^{-4}	4.791×10^{-2}	$(\mathbf{2.096 \times 10^{-2}}, \mathbf{7.068 \times 10^{-2}})$	3.375×10^{3}	1.001
Little Falls Run	4.278×10^{-2}	2.047×10^{-4}	4.284×10^{-2}	$(\mathbf{1.905 \times 10^{-2}}, \mathbf{6.708 \times 10^{-2}})$	3.633×10^{3}	1.000
Little River	-3.596×10^{-3}	3.142×10^{-4}	-3.154×10^{-3}	$(-3.872 \times 10^{-2}, 2.994 \times 10^{-2})$	2.808×10^{3}	1.001
Lompoc	-6.615×10^{-2}	3.653×10^{-4}	-6.626×10^{-2}	$(\mathbf{-0.108}, \mathbf{-2.241 \times 10^{-2}})$	3.508×10^{3}	1.000
Los Banos	-8.492×10^{-2}	4.166×10^{-4}	-8.306×10^{-2}	$(\mathbf{-0.138}, \mathbf{-3.679 \times 10^{-2}})$	3.976×10^{3}	1.001
Loxahatchee	-8.264×10^{-2}	2.779×10^{-4}	-8.274×10^{-2}	$(\mathbf{-0.116}, \mathbf{-4.867 \times 10^{-2}})$	3.901×10^{3}	1.000
MDWASD Central	-6.171×10^{-2}	5.616×10^{-4}	-6.214×10^{-2}	$(\mathbf{-0.118}, \mathbf{-5.187 \times 10^{-3}})$	2.598×10^{3}	1.001
MDWASD North	-1.228×10^{-2}	5.779×10^{-4}	-1.027×10^{-2}	$(-7.043 \times 10^{-2}, 4.236 \times 10^{-2})$	2.253×10^{3}	1.000
MDWASD South	1.013×10^{-2}	3.901×10^{-4}	8.265×10^{-3}	$(-2.948 \times 10^{-2}, 5.416 \times 10^{-2})$	2.805×10^{3}	0.999
Madera	-1.165×10^{-3}	2.996×10^{-4}	-8.443×10^{-4}	$(-3.290 \times 10^{-2}, 3.070 \times 10^{-2})$	2.733×10^{3}	1.000
Mankato	-2.088×10^{-2}	1.875×10^{-4}	-2.096×10^{-2}	$(\mathbf{-4.056 \times 10^{-2}}, \mathbf{-1.933 \times 10^{-3}})$	2.718×10^{3}	1.000
Markshalltown	3.816×10^{-2}	7.722×10^{-4}	3.512×10^{-2}	$(-2.702 \times 10^{-2}, 0.118)$	2.433×10^{3}	1.000
Marlay Taylor	-4.331×10^{-2}	3.959×10^{-4}	-4.371×10^{-2}	$(\mathbf{-8.456 \times 10^{-2}}, \mathbf{-2.298 \times 10^{-3}})$	2.754×10^{3}	1.000
Merced	-5.776×10^{-3}	2.009×10^{-4}	-5.075×10^{-3}	$(-2.828 \times 10^{-2}, 1.454 \times 10^{-2})$	2.851×10^{3}	1.001
Mid-Coastside	-3.936×10^{-2}	3.196×10^{-4}	-3.921×10^{-2}	$(\mathbf{-7.401 \times 10^{-2}}, \mathbf{-5.823 \times 10^{-3}})$	2.906×10^{3}	1.001
Modesto's Sutter	-2.935×10^{-4}	1.525×10^{-4}	-1.968×10^{-4}	$(-1.608 \times 10^{-2}, 1.541 \times 10^{-2})$	2.687×10^{3}	1.003
Montpelier	2.365×10^{-2}	4.577×10^{-4}	2.286×10^{-2}	$(-1.391 \times 10^{-2}, 6.704 \times 10^{-2})$	2.147×10^{3}	1.002
Monterey One	-2.358×10^{-3}	4.791×10^{-4}	-1.724×10^{-3}	$(-4.913 \times 10^{-2}, 4.538 \times 10^{-2})$	2.443×10^{3}	1.001
Morris Forman	8.286×10^{-3}	3.560×10^{-4}	7.774×10^{-3}	$(-2.440 \times 10^{-2}, 3.977 \times 10^{-2})$	1.989×10^{3}	1.001
Mt. Pleasant	-4.491×10^{-3}	6.226×10^{-4}	-3.394×10^{-3}	$(-6.710 \times 10^{-2}, 5.665 \times 10^{-2})$	2.338×10^{3}	1.003
Muscatine	-4.578×10^{-2}	5.750×10^{-4}	-4.640×10^{-2}	$(-8.963 \times 10^{-2}, 6.540 \times 10^{-4})$	1.698×10^{3}	1.001
Norhtwest Water	-5.152×10^{-2}	2.582×10^{-4}	-5.204×10^{-2}	$(\mathbf{-7.909 \times 10^{-2}}, \mathbf{-2.210 \times 10^{-2}})$	3.225×10^{3}	1.001
North Water	1.236×10^{-2}	3.144×10^{-4}	1.163×10^{-2}	$(-1.501 \times 10^{-2}, 4.184 \times 10^{-2})$	2.219×10^{3}	1.000
Novato	-2.629×10^{-2}	3.420×10^{-4}	-2.628×10^{-2}	$(-5.688 \times 10^{-2}, 2.783 \times 10^{-3})$	2.109×10^{3}	1.001
Ocean	4.484×10^{-2}	8.909×10^{-4}	4.163×10^{-2}	$(-4.398 \times 10^{-2}, 0.148)$	3.028×10^{3}	1.001
Oceanside	3.613×10^{-3}	1.230×10^{-4}	3.143×10^{-3}	$(-7.635 \times 10^{-3}, 1.614 \times 10^{-2})$	2.414×10^{3}	1.002
Ottumwa	1.282×10^{-2}	4.182×10^{-4}	1.162×10^{-2}	$(-2.594 \times 10^{-2}, 5.464 \times 10^{-2})$	2.301×10^{3}	1.002
Palo Alto	1.906×10^{-2}	7.980×10^{-5}	1.898×10^{-2}	$(\mathbf{8.876 \times 10^{-3}}, \mathbf{2.934 \times 10^{-2}})$	4.261×10^{3}	1.000
Parker North	8.262×10^{-3}	2.797×10^{-4}	7.332×10^{-3}	$(-2.102 \times 10^{-2}, 3.762 \times 10^{-2})$	2.685×10^{3}	1.001
Parker South	-5.130×10^{-3}	2.982×10^{-4}	-4.088×10^{-3}	$(-3.888 \times 10^{-2}, 2.705 \times 10^{-2})$	2.864×10^{3}	1.000
Paso Robles	-1.157×10^{-2}	1.908×10^{-4}	-1.121×10^{-2}	$(-3.104 \times 10^{-2}, 6.076 \times 10^{-3})$	2.595×10^{3}	1.000
Passaic Valley	-2.885×10^{-2}	3.319×10^{-4}	-2.817×10^{-2}	$(-6.666 \times 10^{-2}, 1.921 \times 10^{-3})$	2.965×10^{3}	0.999
Penacook	2.364×10^{-2}	4.494×10^{-4}	2.369×10^{-2}	$(-1.380 \times 10^{-2}, 6.228 \times 10^{-2})$	1.985×10^{3}	1.001
Portland	-3.303×10^{-2}	3.933×10^{-4}	-3.308×10^{-2}	$(-7.165 \times 10^{-2}, 2.008 \times 10^{-3})$	2.368×10^{3}	1.000
Provo City	-1.506×10^{-3}	2.940×10^{-4}	-1.212×10^{-3}	$(-3.315 \times 10^{-2}, 3.139 \times 10^{-2})$	2.980×10^{3}	0.999
RM Clayton	-1.072×10^{-2}	4.006×10^{-4}	-9.457×10^{-3}	$(-5.176 \times 10^{-2}, 2.692 \times 10^{-2})$	2.421×10^{3}	1.002
Red Wing	4.146×10^{-2}	1.102×10^{-3}	3.927×10^{-2}	$(-2.925 \times 10^{-2}, 0.123)$	1.332×10^{3}	1.003
Regional	-6.640×10^{-2}	4.359×10^{-4}	-6.692×10^{-2}	$(\mathbf{-0.114}, \mathbf{-1.924 \times 10^{-2}})$	2.991×10^{3}	1.001
Regional No. 1	-4.159×10^{-3}	2.330×10^{-4}	-3.398×10^{-3}	$(-3.096 \times 10^{-2}, 2.159 \times 10^{-2})$	3.158×10^{3}	1.000
River Road	-1.641×10^{-2}	5.601×10^{-4}	-1.401×10^{-2}	$(-7.376 \times 10^{-2}, 3.689 \times 10^{-2})$	2.541×10^{3}	1.000
Riverside	-1.600×10^{-2}	5.024×10^{-4}	-1.392×10^{-2}	$(-6.963 \times 10^{-2}, 3.243 \times 10^{-2})$	2.623×10^{3}	1.001
Rochester	-1.469×10^{-2}	2.987×10^{-4}	-1.443×10^{-2}	$(-4.349 \times 10^{-2}, 1.173 \times 10^{-2})$	2.301×10^{3}	1.002
SJRA No. 1	-4.052×10^{-4}	3.521×10^{-4}	-1.992×10^{-4}	$(-4.387 \times 10^{-2}, 4.095 \times 10^{-2})$	3.498×10^{3}	0.999
SJRA No. 2	-4.937×10^{-3}	4.437×10^{-4}	-3.859×10^{-3}	$(-5.475 \times 10^{-2}, 4.479 \times 10^{-2})$	3.128×10^{3}	1.001
SJRA No. 3	5.795×10^{-3}	4.899×10^{-4}	3.615×10^{-3}	$(-4.352 \times 10^{-2}, 5.960 \times 10^{-2})$	2.751×10^{3}	1.000
Sacramento	4.974×10^{-3}	7.567×10^{-5}	4.949×10^{-3}	$(-2.100 \times 10^{-3}, 1.238 \times 10^{-2})$	2.470×10^{3}	1.000
Salina	-4.429×10^{-2}	2.887×10^{-4}	-4.361×10^{-2}	$(\mathbf{-7.832 \times 10^{-2}}, \mathbf{-1.471 \times 10^{-2}})$	3.251×10^{3}	1.001
San Francisco	2.491×10^{-2}	1.299×10^{-4}	2.502×10^{-2}	$(\mathbf{1.006 \times 10^{-2}}, \mathbf{3.960 \times 10^{-2}})$	3.352×10^{3}	1.000
San Jose-Santa Clara	8.667×10^{-3}	8.676×10^{-5}	8.708×10^{-3}	$(-1.748 \times 10^{-4}, 1.767 \times 10^{-2})$	2.886×10^{3}	1.000
San Leandro	-3.335×10^{-2}	6.283×10^{-4}	-3.326×10^{-2}	$(-8.828 \times 10^{-2}, 2.033 \times 10^{-2})$	2.019×10^{3}	1.001

Table S10: Mean, Monte Carlo standard error, median, 95% credible interval, effective sample size, and $\hat{R}$ for coefficient on $\psi^{\cos}_{365.25}$ (yearly basis function) calculated over 4,000 posterior samples. Bolded intervals do not contain 0.

Site	Mean	MCSE	Median	95% CI	ESS	$\hat{R}$
San Mateo & Estero	-8.908×10^{-3}	3.038×10^{-4}	-7.281×10^{-3}	$(-4.426 \times 10^{-2}, 2.091 \times 10^{-2})$	2.833×10^{3}	1.000
Santa Cruz (City)	-8.960×10^{-3}	3.059×10^{-4}	-7.682×10^{-3}	$(-4.216 \times 10^{-2}, 1.839 \times 10^{-2})$	2.428×10^{3}	1.001
Santa Cruz (County)	3.897×10^{-4}	2.854×10^{-4}	5.605×10^{-4}	$(-2.824 \times 10^{-2}, 2.770 \times 10^{-2})$	2.399×10^{3}	1.001
Santa Rosa	-3.538×10^{-3}	1.901×10^{-4}	-3.097×10^{-3}	$(-2.437 \times 10^{-2}, 1.624 \times 10^{-2})$	2.892×10^{3}	1.000
Sausalito-Marin	-2.084×10^{-2}	4.062×10^{-4}	-1.973×10^{-2}	$(-6.247 \times 10^{-2}, 1.480 \times 10^{-2})$	2.349×10^{3}	1.003
Seaford	-1.620×10^{-2}	3.093×10^{-4}	-1.573×10^{-2}	$(-5.068 \times 10^{-2}, 1.613 \times 10^{-2})$	3.064×10^{3}	0.999
Silicon Valley	5.868×10^{-3}	1.581×10^{-4}	5.443×10^{-3}	$(-8.304 \times 10^{-3}, 2.123 \times 10^{-2})$	2.254×10^{3}	1.002
Somerset Raritan	-0.128	3.397×10^{-3}	-0.125	$(-0.362, 7.795 \times 10^{-2})$	1.204×10^{3}	1.003
Soscol	-3.977×10^{-2}	2.236×10^{-4}	-3.977×10^{-2}	$(\mathbf{-6.295 \times 10^{-2}, -1.585 \times 10^{-2}})$	2.846×10^{3}	1.001
South Bay	1.245×10^{-2}	8.572×10^{-4}	1.070×10^{-2}	$(-7.648 \times 10^{-2}, 0.104)$	2.430×10^{3}	1.001
South Bend	7.718×10^{-3}	3.540×10^{-4}	6.604×10^{-3}	$(-3.046 \times 10^{-2}, 4.589 \times 10^{-2})$	2.924×10^{3}	1.000
South Burlington	2.672×10^{-3}	2.895×10^{-4}	2.257×10^{-3}	$(-3.117 \times 10^{-2}, 3.621 \times 10^{-2})$	3.319×10^{3}	1.000
South Columbus	-1.437×10^{-2}	3.194×10^{-4}	-1.401×10^{-2}	$(-4.247 \times 10^{-2}, 1.067 \times 10^{-2})$	1.823×10^{3}	1.003
South County	5.480×10^{-3}	1.587×10^{-4}	5.110×10^{-3}	$(-1.042 \times 10^{-2}, 2.239 \times 10^{-2})$	2.755×10^{3}	1.000
South Laredo	1.405×10^{-2}	4.626×10^{-4}	1.220×10^{-2}	$(-3.152 \times 10^{-2}, 6.400 \times 10^{-2})$	2.571×10^{3}	1.001
South Monmouth	-1.290×10^{-2}	3.855×10^{-4}	-1.161×10^{-2}	$(-5.526 \times 10^{-2}, 2.518 \times 10^{-2})$	2.718×10^{3}	1.001
South River	2.620×10^{-3}	3.734×10^{-4}	1.668×10^{-3}	$(-3.609 \times 10^{-2}, 4.310 \times 10^{-2})$	2.753×10^{3}	1.000
South Water	2.133×10^{-2}	4.138×10^{-4}	2.017×10^{-2}	$(-7.742 \times 10^{-3}, 5.731 \times 10^{-2})$	1.708×10^{3}	1.003
Southern Marin	3.294×10^{-2}	2.390×10^{-4}	3.309×10^{-2}	$(\mathbf{6.592 \times 10^{-3}, 5.923 \times 10^{-2}})$	3.165×10^{3}	0.999
St. Cloud	4.272×10^{-2}	9.079×10^{-4}	4.269×10^{-2}	$(-1.998 \times 10^{-2}, 0.110)$	1.475×10^{3}	1.003
Sunnyvale	1.673×10^{-3}	1.003×10^{-4}	1.316×10^{-3}	$(-8.097 \times 10^{-3}, 1.186 \times 10^{-2})$	2.486×10^{3}	1.000
Traverse City	4.684×10^{-2}	6.955×10^{-4}	4.684×10^{-2}	$(-6.186 \times 10^{-3}, 0.104)$	1.749×10^{3}	1.005
Turkey Creek	5.592×10^{-3}	3.752×10^{-4}	4.330×10^{-3}	$(-3.137 \times 10^{-2}, 4.418 \times 10^{-2})$	2.540×10^{3}	1.000
Turlock	-4.056×10^{-2}	6.444×10^{-4}	-4.013×10^{-2}	$(-0.106, 1.725 \times 10^{-2})$	2.545×10^{3}	1.001
Upper Blackstone	2.703×10^{-2}	5.485×10^{-4}	2.651×10^{-2}	$(-2.621 \times 10^{-2}, 8.411 \times 10^{-2})$	2.672×10^{3}	0.999
Utoy Creek	2.704×10^{-2}	3.971×10^{-4}	2.660×10^{-2}	$(-5.616 \times 10^{-3}, 6.351 \times 10^{-2})$	2.081×10^{3}	1.000
Vallejo	-7.728×10^{-2}	3.374×10^{-4}	-7.726×10^{-2}	$(\mathbf{-0.118, -3.450 \times 10^{-2}})$	3.921×10^{3}	1.001
Valley	-2.797×10^{-2}	2.699×10^{-4}	-2.826×10^{-2}	$(\mathbf{-5.483 \times 10^{-2}, -2.952 \times 10^{-4}})$	2.744×10^{3}	1.001
Valley Creek	3.449×10^{-3}	3.282×10^{-4}	2.576×10^{-3}	$(-2.856 \times 10^{-2}, 3.618 \times 10^{-2})$	2.385×10^{3}	1.001
Village Creek	-6.679×10^{-2}	2.959×10^{-4}	-6.733×10^{-2}	$(\mathbf{-0.102, -3.004 \times 10^{-2}})$	3.684×10^{3}	1.001
Warren	-2.505×10^{-2}	3.636×10^{-4}	-2.512×10^{-2}	$(-6.126 \times 10^{-2}, 8.720 \times 10^{-3})$	2.617×10^{3}	1.000
Weaton	-1.735×10^{-2}	2.308×10^{-4}	-1.719×10^{-2}	$(-4.016 \times 10^{-2}, 2.997 \times 10^{-3})$	2.404×10^{3}	1.002
West Boise	6.563×10^{-2}	7.720×10^{-4}	6.682×10^{-2}	$(\mathbf{5.924 \times 10^{-4}, 0.127})$	1.832×10^{3}	1.000
West County	-2.200×10^{-2}	3.936×10^{-4}	-2.172×10^{-2}	$(-6.015 \times 10^{-2}, 1.421 \times 10^{-2})$	2.371×10^{3}	1.001
Wheeling	-1.852×10^{-3}	3.267×10^{-4}	-1.435×10^{-3}	$(-3.694 \times 10^{-2}, 3.249 \times 10^{-2})$	2.760×10^{3}	1.000
Wichita Falls	1.434×10^{-2}	3.617×10^{-4}	1.368×10^{-2}	$(-1.483 \times 10^{-2}, 4.626 \times 10^{-2})$	1.882×10^{3}	1.001
Windsor	-8.700×10^{-3}	5.312×10^{-4}	-6.464×10^{-3}	$(-6.629 \times 10^{-2}, 4.578 \times 10^{-2})$	2.639×10^{3}	1.000
Winters	-4.004×10^{-2}	6.098×10^{-4}	-4.069×10^{-2}	$(-9.268 \times 10^{-2}, 1.274 \times 10^{-2})$	2.035×10^{3}	1.001
Wolcott	1.522×10^{-2}	6.204×10^{-4}	1.177×10^{-2}	$(-4.634 \times 10^{-2}, 8.456 \times 10^{-2})$	2.689×10^{3}	1.000
Woodland	-3.565×10^{-3}	4.286×10^{-4}	-2.290×10^{-3}	$(-5.181 \times 10^{-2}, 4.264 \times 10^{-2})$	2.881×10^{3}	1.000
Yankton	7.202×10^{-2}	9.825×10^{-4}	7.242×10^{-2}	$(-5.259 \times 10^{-3}, 0.153)$	1.782×10^{3}	1.002
York	-1.703×10^{-2}	5.068×10^{-4}	-1.579×10^{-2}	$(-6.678 \times 10^{-2}, 2.901 \times 10^{-2})$	2.332×10^{3}	1.000
Youngstown	-1.060×10^{-2}	3.481×10^{-4}	-9.524×10^{-3}	$(-4.549 \times 10^{-2}, 2.324 \times 10^{-2})$	2.426×10^{3}	1.000
Zacate Creek	-2.637×10^{-2}	5.210×10^{-4}	-2.425×10^{-2}	$(-8.348 \times 10^{-2}, 1.889 \times 10^{-2})$	2.608×10^{3}	1.000

Table S11: Site name abbreviations. Sites ordered alphabetically by abbreviation.

Site Name	Abbreviation
[Fremont Basin] - Raymond A. Boege Alvarado WWTP	Boege Alvarado (Fremont)

Table S11: Site name abbreviations. Sites ordered alphabetically by abbreviation.

Site Name	Abbreviation
[Newark Basin] - Raymond A. Boege Alvarado WWTP	Boege Alvarado (Newark)
[Union City Basin] - Raymond A. Boege Alvarado WWTP	Boege Alvarado (Union City)
Akron Water Reclamation Facility	Akron
Altamonte Springs Regional Water Reclamation Facility	Altamonte Springs
Aquia Wastewater Treatment Facility	Aquia
Archie Elledge WWTP	Archie Elledge
Bayshore Regional Sewerage Authority	Bayshore
Big Creek Water Reclamation Facility	Big Creek
Brunswick Sewer District	Brunswick
Cahaba River Water Reclamation Facility	Cahaba River
Calera Creek Water Recycling Plant	Calera Creek
Camp Creek Water Reclamation Facility	Camp Creek
Capital Region Water AWTF	Capital Region
Central Contra Costa Sanitary District	Central Contra Costa
Central Marin Sanitation Agency	Central Marin
Central Marin Sanitation Agency - West Railroad	Central Marin (W Railroad)
Central Valley Water Reclamation Facility	Central Valley
City of Ann Arbor Wastewater Treatment Plant	Ann Arbor
City of Bangor Wastewater Treatment Plant	Bangor
City of Carmel WWTP	Carmel
City of Clinton	Clinton
City of Coeur d'Alene Water Resource Recovery Facility	Coeur d'Alene
City of Davis Wastewater Treatment Plant	Davis
City of Dover Wastewater Treatment Facility	Dover
City of Essex Junction Wastewater Treatment Facility	Essex
City of Gainesville Wastewater Treatment Plant	Gainesville
City of Garland Rowlett Creek WWTP	Garland Rowlett Creek
City of Harrison Wastewater Treatment Plant	Harrison
City of Hollister Domestic Water Recycling Facility	Hollister
City of Madera, Wastewater Treatment Plant	Madera
City of Mankato Water Resource Recovery Facility (WRRF)	Mankato
City of Marshalltown Water Pollution Control Plant	Markshalltown
City of Paso Robles Wastewater Treatment Plant	Paso Robles
City Of Rochester MN Water Reclamation Plant	Rochester
City of San Leandro Water Pollution Control Plant	San Leandro
City of San Mateo & Estero M.I.D. Water Quality Control Plant	San Mateo & Estero
City of Santa Cruz WTF - City Influent	Santa Cruz (City)
City of Santa Cruz WTF - County Influent	Santa Cruz (County)
City of Santa Rosa, Laguna Treatment Plant	Santa Rosa
City of South Bend Wastewater Treatment Plant	South Bend
City of Sunnyvale Water Pollution Control Plant	Sunnyvale
City of Warren Wastewater Treatment Plant	Warren
City of Wheeling, Water Pollution Control Division	Wheeling
City of Yankton Wastewater Treatment Facility	Yankton
City of Youngstown Wastewater Treatment Plant	Youngstown
Clark County Water Reclamation District (CCWRD) Flamingo Water Resource Center (FWRC)	Clark County
Coastal Treatment Plant	Coastal
CODIGA	CODIGA
Coralville Wastewater Treatment Facility	Coralville
Cumberland County Utilities Authority	Cumberland
Deer Island Treatment Plant	Deer Island
DELCORA Western Regional Treatment Plant	DELCORA
Dillman Road WWTP	Dillman Road

Table S11: Site name abbreviations. Sites ordered alphabetically by abbreviation.

Site Name	Abbreviation
Duck Creek Wastewater Treatment Plant	Duck Creek
E.W. Blom Point Loma Wastewater Treatment Plant	E.W. Blom Point Loma
East Bay Municipal Utility District	East Bay
Eastern Water Reclamation Facility	Eastern
Ellis Creek Water Recycling Facility	Ellis Creek
Esparto Wastewater Treatment Facility	Esparto
Fairfield-Suisun Sewer District	Fairfield-Suisun
Five Mile Creek Water Reclamation Facility	Five Mile Creek
Glenbard Wastewater Authority	Glenbard
Grandville Clean Water Plant	Grandville
Hagerstown Wastewater Treatment Plant	Hagerstown
Hall Street Wastewater Treatment Plant	Hall Street
Hamlin Water Reclamation Facility	Hamlin
Hollywood Road WWTP	Hollywood Road
Hyperion Water Reclamation Plant (HWRP)	Hyperion
Jackson Wastewater Treatment Plant	Jackson
JB Latham Treatment Plant	JB Latham
Jeffersonville Downtown WWTP	Jeffersonville
John M. Asplund Water Pollution Control Facility	John M. Asplund
Johnnie Mosley Regional Water Reclamation Facility	Johnnie Mosley
Johns Creek Environmental Campus	Johns Creek
Joint Water Pollution Control Plant	Joint
Kansas City Treatment Plant #20	Kansas City
Lancaster Water Reclamation Plant	Lancaster
Lander Street Water Renewal Facility	Lander Street
Las Gallinas Valley Sanitary District	Las Gallinas
Lawrence Kansas River Wastewater Treatment Facility	Lawrence Kansas
Little Falls Run Wastewater Treatment Facility	Little Falls Run
Little River Water Reclamation Facility	Little River
Lompoc Regional Wastewater Reclamation Plant	Lompoc
Los Banos Wastewater Treatment Plant	Los Banos
Loxahatchee River Environmental Control District	Loxahatchee
Marlay Taylor Water Reclamation Facility	Marlay Taylor
MDWASD Central District WWTP	MDWASD Central
MDWASD North District WWTF	MDWASD North
MDWASD South District WWTF	MDWASD South
Merced Wastewater Treatment Plant	Merced
Modesto's Sutter Primary Treatment Facility	Modesto's Sutter
Monterey One Water - Regional Treatment Plant	Monterey One
Montpelier Water Resource Recovery Facility	Montpelier
Morris Forman Water Quality Treatment Center	Morris Forman
Mt. Pleasant WRRF	Mt. Pleasant
Municipal Wastewater Treatment Plant No. 1 (Kaw Point)	Kaw Point
Muscatine STP	Muscatine
North Water Reclamation Facility	North Water
Northwest Water Reclamation Facility	Norhtwest Water
Novato Sanitary District	Novato
Oceanside Water Pollution Control Plant	Oceanside
Ottumwa WPCF	Ottumwa
Palo Alto Regional Water Quality Control Plant	Palo Alto
Parker Water and Sanitation District North Water Reclamation Facility	Parker North
Parker Water and Sanitation District South Water Reclamation Facility	Parker South
Passaic Valley Sewerage Commission	Passaic Valley

Table S11: Site name abbreviations. Sites ordered alphabetically by abbreviation.

Site Name	Abbreviation
Penacook Wastewater Treatment Facility	Penacook
Portland Water District (East End Wastewater Treatment Facility)	Portland
Provo City Water Reclamation Facility	Provo City
Red Wing Wastewater Treatment Facility	Red Wing
Regional Treatment Plant	Regional
Regional Water Recycling Plant No.1 (RP-1)	Regional No. 1
River Road WWTP	River Road
Riverside Water Quality Control Plant	Riverside
RM Clayton Water Reclamation Center	RM Clayton
Sacramento Regional Wastewater Treatment Plant	Sacramento
Salina Wastewater Treatment Plant	Salina
San Jose-Santa Clara Regional Wastewater Facility	San Jose-Santa Clara
Sausalito-Marin City Sanitary District	Sausalito-Marin
Seaford Wastewater Treatment Facility	Seaford
Sewer Authority Mid-Coastside	Mid-Coastside
Sewerage Agency of Southern Marin Wastewater Treatment Plant	Southern Marin
Silicon Valley Clean Water	Silicon Valley
SJRA WWTF No.1	SJRA No. 1
SJRA WWTF No.2	SJRA No. 2
SJRA WWTF No.3	SJRA No. 3
Soscol Water Recycling Facility	Soscol
South Bay International Wastewater Treatment Plant	South Bay
South Burlington-Airport Parkway WWTF	South Burlington
South Columbus Water Resources Facility	South Columbus
South County Regional Wastewater Authority	South County
South Laredo WWTP	South Laredo
South Monmouth Regional Sewerage Authority	South Monmouth
South River Water Reclamation Center	South River
South Water Reclamation Facility	South Water
Southeast San Francisco	San Francisco
St. Cloud Nutrient, Energy and Water Recovery Facility	St. Cloud
The Somerset Raritan Valley Sewerage Authority	Somerset Raritan
Town of Hillsville Wastewater Treatment Plant	Hillsville
Township of Ocean Sewerage Authority	Ocean
Traverse City Regional Waste Water Treatment Plant	Traverse City
Turkey Creek Water Reclamation Facility	Turkey Creek
Turlock Regional Water Quality Control Facility	Turlock
Upper Blackstone Clean Water	Upper Blackstone
Utoy Creek Water Reclamation Center	Utoy Creek
Vallejo Flood and Wastewater District Wastewater Treatment Plant	Vallejo
Valley Creek Water Reclamation Facility	Valley Creek
Valley Sanitary District	Valley
Village Creek Water Reclamation Facility	Village Creek
West Boise Water Renewal Facility	West Boise
West County Wastewater District	West County
Wheaton Sanitary District	Weaton
Wichita Falls Resource Recovery Facility	Wichita Falls
Windsor Wastewater Treatment, Reclamation, and Disposal Facility	Windsor
Winters - East Street Pump Station	Winters
Wolcott Wastewater Treatment Facility	Wolcott
Woodland Water Pollution Control Facility	Woodland
York Sewer District	York
Zacate Creek WWTP	Zacate Creek

Table S12: Number of observations used in autocorrelation calculations by site and by lag for lags of 0 to 14 days.

Site Name	lag=0	lag=1	lag=2	lag=3	lag=4	lag=5	lag=6	lag=7	lag=8	lag=9	lag=10	lag=11	lag=12	lag=13	lag=14
Akron	85	0	79	48	46	80	0	83	0	78	46	46	80	0	84
Altamonte Springs	117	2	116	71	69	112	5	114	5	112	69	67	110	5	113
Ann Arbor	174	32	141	105	117	134	66	144	70	131	115	104	137	62	151
Aquia	73	4	71	46	44	71	6	71	6	69	45	43	69	6	71
Archie Elledge	139	9	131	76	78	126	20	128	16	124	71	75	120	16	128
Bangor	30	20	18	16	14	14	18	30	16	16	14	10	10	14	28
Bayshore	48	0	47	32	30	47	0	48	0	45	30	28	45	0	48
Big Creek	173	170	115	0	3	110	168	171	167	108	2	2	106	165	169
Boege Alvarado (Fremont)	75	4	64	2	2	62	5	72	7	62	2	2	62	5	70
Boege Alvarado (Newark)	76	0	68	2	2	68	2	75	2	66	2	2	66	2	73
Boege Alvarado (Union City)	74	2	64	0	0	64	3	70	3	64	0	0	64	3	71
Brunswick	100	96	62	0	2	62	93	99	90	56	2	2	56	88	98
Cahaba River	132	4	124	84	80	122	6	129	6	116	76	75	114	6	128
Calera Creek	125	21	112	71	70	113	25	120	26	111	76	74	109	25	117
Camp Creek	175	168	112	11	10	115	166	169	164	112	11	11	115	165	166
Capital Region	163	6	158	107	105	158	9	160	9	156	105	103	156	9	160
Carmel	46	5	42	27	28	41	6	44	5	41	26	26	39	6	44
Central Contra Costa	200	122	18	172	176	20	132	187	130	18	171	172	20	130	188
Central Marin	95	6	82	0	4	72	19	90	17	70	2	6	68	22	84
Central Marin (W Railroad)	97	2	88	0	2	86	6	96	5	84	0	4	80	11	94
Central Valley	125	12	117	79	80	113	22	119	22	112	77	79	112	23	115
Clark County	62	2	61	39	39	60	3	61	3	59	37	37	58	3	61
Clinton	91	0	91	58	58	90	0	91	0	89	56	56	88	0	91
Coastal	103	0	103	56	73	88	26	95	24	90	63	69	84	30	89
CODIGA	600	581	597	579	574	595	581	594	580	592	578	579	591	580	595
Coeur d'Alene	226	18	202	131	133	197	36	211	32	198	131	131	195	36	211
Coralville	86	0	85	54	54	83	0	86	0	82	52	52	81	0	86
Cumberland	64	0	64	38	36	63	0	64	0	62	34	34	60	0	63
Davis	678	582	678	645	644	678	584	678	585	676	644	643	676	586	677
Deer Island	101	3	94	62	63	94	3	100	3	91	61	64	92	3	98
DELCORA	110	6	102	60	56	97	11	102	11	94	60	53	99	8	101
Dillman Road	147	98	96	76	74	100	92	140	96	96	72	76	100	91	138
Dover	104	69	66	52	52	70	64	95	64	76	50	46	72	65	91
Duck Creek	171	14	148	103	108	149	18	160	21	147	100	104	146	22	157
E.W. Blom Point Loma	154	103	96	98	96	94	100	154	99	96	94	94	92	98	152
East Bay	238	235	160	8	14	158	236	233	236	158	12	14	156	234	230
Eastern	195	10	174	120	121	166	31	178	31	165	117	116	161	30	176

Table S12: Number of observations used in autocorrelation calculations by site and by lag for lags of 0 to 14 days.

Site Name	lag=0	lag=1	lag=2	lag=3	lag=4	lag=5	lag=6	lag=7	lag=8	lag=9	lag=10	lag=11	lag=12	lag=13	lag=14
Ellis Creek	141	109	90	47	50	94	114	119	112	93	57	51	100	108	117
Esparto	103	0	99	62	61	98	4	98	5	95	59	57	96	2	100
Essex	65	0	60	40	38	59	0	63	0	57	38	36	59	0	65
Fairfield-Suisun	137	2	135	89	87	135	3	136	3	133	87	85	133	3	136
Five Mile Creek	123	0	120	72	77	117	3	121	2	117	68	71	112	2	122
Gainesville	91	0	88	54	53	86	4	91	4	80	49	51	78	8	84
Garland Rowlett Creek	223	18	211	146	142	210	23	212	27	210	149	134	210	23	211
Glenbard	150	87	102	86	84	102	84	147	87	100	82	81	100	82	144
Grandville	108	7	103	72	71	102	7	105	7	102	70	67	102	7	105
Hagerstown	100	10	92	60	53	95	16	90	15	89	59	56	85	15	93
Hall Street	132	2	131	85	83	131	3	131	3	129	83	81	129	3	131
Hamlin	106	2	93	69	60	93	10	97	10	88	61	58	83	8	95
Harrison	48	4	40	26	33	35	17	39	17	35	31	28	36	16	39
Hillsville	105	13	98	61	65	94	18	98	19	95	61	58	91	20	96
Hollister	133	0	129	81	79	126	3	132	3	123	81	74	124	3	132
Hollywood Road	106	101	68	6	6	68	105	102	102	66	4	8	66	103	101
Hyperion	151	111	100	76	76	100	118	142	116	100	74	76	98	117	140
Jackson	206	16	194	134	131	192	30	197	29	188	133	128	190	34	190
JB Latham	103	0	103	56	73	88	26	95	24	90	63	69	84	30	89
Jeffersonville	121	2	120	73	70	120	3	120	2	118	69	67	118	3	119
John M. Asplund	34	30	18	6	8	26	28	32	23	18	8	8	22	26	26
Johnnie Mosley	103	26	83	52	58	79	35	92	33	78	50	54	77	40	81
Johns Creek	172	168	113	2	4	110	166	169	165	106	4	4	106	165	167
Joint	248	164	162	160	160	162	164	246	164	160	160	158	160	162	246
Kansas City	95	95	62	0	6	62	94	91	93	60	6	4	60	92	94
Kaw Point	94	94	60	0	6	62	93	90	91	58	6	4	58	90	93
Lancaster	145	101	88	90	90	92	102	136	101	91	88	88	90	98	140
Lander Street	92	0	92	60	60	91	0	92	0	90	58	58	89	0	92
Las Gallinas	163	79	132	65	79	127	86	146	84	130	75	75	131	82	150
Lawrence Kansas	150	0	144	88	86	142	0	149	0	142	86	86	142	0	149
Little Falls Run	72	4	70	44	42	70	6	70	6	68	43	41	68	6	70
Little River	172	172	112	0	0	110	168	172	166	106	0	0	106	165	172
Lompoc	162	27	145	99	95	145	32	154	34	144	97	93	145	36	148
Los Banos	113	77	76	70	72	74	82	109	77	74	68	70	72	81	108
Loxahatchee	142	0	138	94	92	138	2	140	2	136	92	90	137	2	141
Madera	68	0	65	44	44	63	0	68	0	62	42	42	61	0	68
Mankato	149	35	126	88	79	127	49	129	47	127	85	83	125	46	136

Table S12: Number of observations used in autocorrelation calculations by site and by lag for lags of 0 to 14 days.

Site Name	lag=0	lag=1	lag=2	lag=3	lag=4	lag=5	lag=6	lag=7	lag=8	lag=9	lag=10	lag=11	lag=12	lag=13	lag=14
Markshalltown	87	7	84	51	52	83	8	86	8	81	48	51	80	9	84
Marlay Taylor	83	0	78	46	42	78	0	79	0	74	42	44	73	0	82
MDWASD Central	79	77	52	4	14	51	73	69	67	46	13	12	40	65	68
MDWASD North	91	8	83	58	58	82	12	86	11	82	55	56	81	10	87
MDWASD South	88	10	74	58	56	74	14	82	15	71	56	55	70	14	83
Merced	303	195	300	264	263	301	195	301	195	299	264	261	298	195	302
Mid-Coastside	182	16	165	106	98	166	25	165	27	159	101	93	162	28	166
Modesto's Sutter	294	261	258	245	245	256	265	287	264	256	242	243	253	261	289
Montpelier	67	42	40	42	42	40	40	67	40	38	40	40	38	38	66
Monterey One	107	27	86	60	63	82	45	85	43	77	63	60	76	54	88
Morris Forman	75	0	0	0	0	8	14	62	25	6	0	2	12	18	50
Mt. Pleasant	52	0	51	28	30	46	4	49	6	45	29	27	44	4	47
Muscatine	107	73	68	61	61	67	78	99	74	66	63	57	66	73	94
Norhtwest Water	196	7	182	124	121	182	17	187	17	179	115	117	170	17	183
North Water	119	2	116	71	68	116	3	117	2	114	67	65	114	3	118
Novato	176	4	174	108	106	173	8	173	8	171	105	104	169	7	172
Ocean	37	0	36	0	0	34	0	37	0	34	0	0	32	0	37
Oceanside	776	774	773	776	775	775	773	776	775	773	773	771	773	773	769
Ottumwa	104	20	88	64	61	90	27	93	27	89	62	62	89	25	95
Palo Alto	805	805	803	805	805	805	805	805	805	804	804	804	804	804	804
Parker North	191	186	122	5	12	121	183	184	185	118	13	13	119	180	184
Parker South	192	189	122	5	12	119	187	185	188	118	13	13	117	184	185
Paso Robles	249	162	166	164	164	164	162	249	160	164	162	162	162	160	249
Passaic Valley	146	103	87	54	51	96	103	115	109	89	52	46	92	104	124
Penacook	130	2	128	83	81	127	3	128	3	125	81	79	124	3	129
Portland	111	19	85	56	51	85	28	83	31	77	44	54	72	33	80
Provo City	95	8	84	2	2	84	14	90	15	80	4	4	78	17	88
Red Wing	36	11	29	19	18	29	13	31	13	28	17	18	24	11	28
Regional	102	0	102	54	71	87	26	93	24	88	61	67	83	30	88
Regional No. 1	193	134	124	112	113	122	137	190	132	123	108	108	118	136	185
River Road	103	98	68	2	2	64	93	99	93	64	2	0	62	92	101
Riverside	88	8	84	54	52	84	12	84	12	82	53	51	82	12	83
RM Clayton	116	9	106	71	69	104	11	109	14	102	66	66	103	14	109
Rochester	122	8	117	76	74	117	12	117	12	115	75	73	114	10	118
Sacramento	812	812	812	812	812	812	812	812	812	812	812	812	812	812	812
Salina	91	0	90	60	60	89	0	91	0	88	58	58	87	0	91
San Francisco	449	449	449	449	449	449	449	449	449	449	449	448	449	449	449

Table S12: Number of observations used in autocorrelation calculations by site and by lag for lags of 0 to 14 days.

Site Name	lag=0	lag=1	lag=2	lag=3	lag=4	lag=5	lag=6	lag=7	lag=8	lag=9	lag=10	lag=11	lag=12	lag=13	lag=14
San Jose-Santa Clara	810	810	810	810	810	810	810	810	810	809	809	809	809	809	809
San Leandro	141	141	94	0	0	92	139	141	139	92	0	0	90	137	141
San Mateo & Estero	162	13	149	94	93	150	16	150	18	148	90	89	147	17	153
Santa Cruz (City)	209	12	198	127	129	197	24	199	26	195	126	127	193	25	195
Santa Cruz (County)	210	12	199	129	131	198	26	199	28	196	128	129	194	27	197
Santa Rosa	155	117	90	85	90	104	104	138	98	93	91	91	102	104	134
Sausalito-Marin	134	130	82	4	12	76	122	126	123	76	8	10	68	118	126
Seaford	81	0	81	52	50	81	0	81	0	79	50	48	79	0	81
Silicon Valley	808	808	808	808	808	808	808	808	808	807	807	807	807	806	807
SJRA No. 1	77	0	77	50	50	76	0	77	0	75	48	48	74	0	77
SJRA No. 2	77	0	77	50	50	76	0	77	0	75	48	48	74	0	77
SJRA No. 3	77	0	77	50	50	76	0	77	0	75	48	48	74	0	77
Somerset Raritan	39	0	38	24	24	36	2	38	2	36	22	22	34	2	38
Soscol	134	2	131	83	85	128	3	132	3	127	81	81	124	3	132
South Bay	36	27	20	19	14	24	24	30	29	17	14	15	21	22	30
South Bend	130	4	125	80	82	120	6	127	6	120	78	78	118	6	127
South Burlington	39	16	16	2	2	20	17	34	18	18	2	2	18	17	31
South Columbus	118	10	102	64	60	99	18	104	18	93	57	54	93	16	98
South County	810	810	810	810	810	810	810	810	810	809	809	809	809	809	809
South Laredo	94	2	89	54	50	90	2	91	2	89	44	42	86	3	88
South Monmouth	109	21	100	60	58	99	29	102	32	97	62	55	97	31	100
South River	115	6	106	69	68	104	9	109	11	102	65	65	103	11	109
South Water	195	2	182	125	118	181	12	187	13	178	116	117	171	13	183
Southern Marin	107	79	58	61	61	70	77	92	81	72	60	59	64	80	91
St. Cloud	59	14	52	33	33	51	15	55	15	53	29	35	49	15	52
Sunnyvale	805	805	805	805	805	805	805	805	805	804	804	804	804	804	804
Traverse City	88	58	56	56	54	54	58	85	58	54	54	52	52	56	86
Turkey Creek	125	3	116	80	77	112	2	119	3	111	75	73	111	3	122
Turlock	111	0	110	74	72	110	0	111	0	108	72	70	108	0	111
Upper Blackstone	69	2	67	39	40	64	7	65	7	63	38	38	62	7	66
Utoy Creek	115	6	106	69	68	104	9	109	11	102	65	65	103	11	109
Vallejo	143	142	96	2	4	94	139	141	139	94	4	4	92	137	141
Valley	154	0	154	102	100	154	0	154	0	152	100	98	152	0	154
Valley Creek	158	0	158	104	104	157	0	158	0	156	102	102	155	0	158
Village Creek	150	13	136	92	91	134	19	141	18	135	92	90	133	20	144
Warren	137	97	91	74	74	99	93	124	90	99	69	70	94	90	132
Weaton	141	0	141	88	86	141	0	141	0	139	86	84	139	0	140

Table S12: Number of observations used in autocorrelation calculations by site and by lag for lags of 0 to 14 days.

Site Name	lag=0	lag=1	lag=2	lag=3	lag=4	lag=5	lag=6	lag=7	lag=8	lag=9	lag=10	lag=11	lag=12	lag=13	lag=14
West Boise	92	0	92	60	60	91	0	92	0	90	58	58	89	0	92
West County	151	92	82	64	63	75	91	130	92	73	61	63	76	85	133
Wheeling	52	0	52	28	28	50	0	52	0	50	28	26	48	0	52
Wichita Falls	107	15	95	63	65	92	19	103	20	95	63	62	90	21	99
Windsor	95	0	92	54	53	89	7	89	9	84	54	50	84	10	88
Winters	101	17	83	61	56	82	23	93	20	85	58	51	84	19	90
Wolcott	93	91	58	4	10	60	90	84	87	58	14	10	60	88	86
Woodland	108	0	103	72	70	103	0	108	0	100	70	68	101	0	107
Yankton	47	4	44	28	26	43	7	46	8	40	25	23	40	9	42
York	88	65	56	46	46	54	65	85	64	52	42	44	52	64	81
Youngstown	103	13	93	63	58	95	18	95	18	90	60	60	89	17	98
Zacate Creek	92	2	85	54	48	86	2	89	2	85	42	40	83	2	84

Table S13: Number of observations used in autocorrelation calculations by site and by lag for lags of 15 to 30 days.

Site Name	lag=15	lag=16	lag=17	lag=18	lag=19	lag=20	lag=21	lag=22	lag=23	lag=24	lag=25	lag=26	lag=27	lag=28	lag=29	lag=30
Akron	0	77	46	44	79	0	85	0	73	44	40	75	0	83	0	71
Altamonte Springs	5	109	65	65	108	5	113	5	104	63	61	104	5	107	5	101
Ann Arbor	61	142	97	112	133	64	139	67	129	111	100	133	58	145	60	131
Aquia	6	67	42	40	67	5	73	5	65	40	38	65	6	71	6	62
Archie Elledge	19	118	80	66	123	16	130	17	121	75	69	117	15	129	17	119
Bangor	12	12	10	8	8	10	26	8	8	6	6	6	8	15	6	6
Bayshore	0	43	28	26	43	0	48	0	41	26	24	41	0	48	0	39
Big Creek	165	106	2	2	104	162	168	162	104	2	0	102	160	170	160	102
Boege Alvarado (Fremont)	7	58	2	0	60	7	69	8	56	2	2	56	6	69	8	56
Boege Alvarado (Newark)	2	66	2	2	64	2	72	2	66	0	2	64	2	74	2	62
Boege Alvarado (Union City)	2	64	0	0	60	3	70	3	60	0	0	58	2	70	3	62
Brunswick	86	54	2	2	54	83	91	83	52	2	2	52	83	96	84	52
Cahaba River	6	113	72	72	111	6	123	5	109	69	66	110	4	125	4	105
Calera Creek	28	109	74	70	108	29	115	26	102	70	71	105	29	112	28	100
Camp Creek	163	113	7	11	111	161	168	161	105	9	10	109	161	167	160	105
Capital Region	9	154	104	103	153	9	159	9	152	101	100	151	9	159	8	150
Carmel	6	38	25	25	36	6	42	6	35	22	24	35	6	41	5	33
Central Contra Costa	126	20	169	167	20	126	183	124	20	167	166	18	128	186	124	18
Central Marin	19	66	2	6	68	24	81	20	64	3	4	67	22	85	21	60
Central Marin (W Railroad)	7	80	0	4	78	13	94	9	76	0	6	74	17	93	11	72
Central Valley	23	109	77	76	109	23	115	19	110	70	76	110	19	116	20	108
Clark County	3	57	35	35	56	3	61	3	55	33	33	54	3	61	3	53
Clinton	0	87	54	54	86	0	91	0	85	52	52	84	0	91	0	83
Coastal	27	85	64	64	85	28	91	24	86	62	64	83	26	88	22	82
CODIGA	579	589	575	572	587	579	593	578	587	574	574	583	578	592	578	581
Coeur d'Alene	34	191	132	121	191	37	202	30	196	125	124	189	37	203	39	187
Coralville	0	80	50	50	78	0	84	0	78	48	48	76	0	83	0	76
Cumberland	0	59	32	32	59	0	63	0	57	32	30	56	0	64	0	56
Davis	587	675	644	644	676	590	678	591	675	645	644	675	592	677	593	673
Deer Island	3	90	59	60	90	2	100	2	91	57	58	88	2	100	2	88
DELCORA	9	96	48	59	94	10	101	10	91	52	51	96	8	101	11	96
Dillman Road	93	102	70	70	98	87	137	88	96	70	68	96	93	136	84	92
Dover	61	72	46	46	70	58	93	59	64	44	46	70	55	93	65	64
Duck Creek	20	147	98	103	142	18	166	17	146	94	97	141	18	163	18	145
E.W. Blom Point Loma	98	92	92	92	90	96	151	96	88	90	90	88	94	152	94	86
East Bay	234	156	14	14	154	231	232	231	154	14	14	152	230	229	230	152
Eastern	33	158	116	118	161	28	171	31	158	113	120	151	31	173	25	156

Table S13: Number of observations used in autocorrelation calculations by site and by lag for lags of 15 to 30 days.

Site Name	lag=15	lag=16	lag=17	lag=18	lag=19	lag=20	lag=21	lag=22	lag=23	lag=24	lag=25	lag=26	lag=27	lag=28	lag=29	lag=30
Ellis Creek	112	93	55	57	93	111	122	110	91	51	52	93	104	119	109	82
Esparto	5	94	57	55	92	5	99	5	91	55	53	90	3	99	5	89
Essex	0	54	36	34	55	0	64	0	52	34	32	54	0	62	0	50
Fairfield-Suisun	3	130	86	83	131	3	135	3	128	84	81	129	3	135	3	126
Five Mile Creek	3	111	62	67	106	3	121	3	106	59	61	102	3	120	3	101
Gainesville	8	75	49	50	75	8	84	6	76	47	48	75	4	86	4	75
Garland Rowlett Creek	28	203	145	132	206	17	215	32	199	149	119	208	23	212	35	196
Glenbard	82	98	82	82	96	80	145	80	96	78	78	96	78	143	78	94
Grandville	7	100	68	65	100	7	104	7	98	66	63	98	7	104	7	96
Hagerstown	16	91	56	53	87	14	98	16	86	60	50	88	16	91	17	84
Hall Street	3	127	81	79	127	3	131	3	125	79	77	125	3	131	3	123
Hamlin	11	83	61	55	84	10	92	11	79	56	59	78	9	94	7	78
Harrison	18	34	25	27	33	17	35	17	36	23	22	33	15	37	17	32
Hillsville	20	92	65	57	92	17	95	18	92	58	58	87	16	97	19	90
Hollister	3	119	75	73	119	3	130	3	118	73	71	117	3	131	3	116
Hollywood Road	100	62	6	8	64	100	103	97	60	6	8	62	98	98	95	60
Hyperion	114	98	72	76	98	115	143	112	96	70	74	96	117	138	111	94
Jackson	34	185	132	124	188	31	190	33	183	131	126	183	33	190	33	180
JB Latham	27	85	64	64	85	28	91	24	86	62	64	83	26	88	22	82
Jeffersonville	3	115	68	65	115	3	117	3	114	65	63	113	3	120	3	111
John M. Asplund	23	18	6	8	22	22	23	21	20	4	6	16	19	28	24	16
Johnnie Mosley	38	75	52	50	70	39	80	40	63	53	45	67	34	80	37	68
Johns Creek	164	104	4	4	104	162	167	162	104	4	2	102	160	168	159	100
Joint	162	158	156	155	161	159	245	160	156	154	154	156	158	246	158	154
Kansas City	91	58	4	4	58	90	94	88	56	4	6	56	89	91	85	54
Kaw Point	90	56	4	4	56	88	93	87	54	4	6	56	88	90	83	52
Lancaster	99	90	88	86	88	98	138	98	88	86	84	86	96	138	97	84
Lander Street	0	88	56	56	87	0	92	0	86	54	54	85	0	92	0	84
Las Gallinas	79	130	69	82	124	82	144	82	127	74	73	125	83	144	85	123
Lawrence Kansas	0	140	82	84	139	0	148	0	138	82	80	134	0	145	0	136
Little Falls Run	6	65	40	38	66	5	71	5	63	38	36	64	6	69	6	60
Little River	164	104	0	0	104	162	169	161	102	0	0	102	160	169	159	100
Lompoc	36	142	94	93	136	35	150	37	137	96	94	134	34	149	39	136
Los Banos	74	73	66	68	70	78	107	74	70	64	67	66	76	106	72	66
Loxahatchee	2	134	90	88	135	2	141	2	133	86	86	132	2	141	2	130
Madera	0	60	40	40	60	0	68	0	58	38	38	57	0	68	0	56
Mankato	45	125	85	83	122	45	133	46	122	84	76	121	46	129	48	122

Table S13: Number of observations used in autocorrelation calculations by site and by lag for lags of 15 to 30 days.

Site Name	lag=15	lag=16	lag=17	lag=18	lag=19	lag=20	lag=21	lag=22	lag=23	lag=24	lag=25	lag=26	lag=27	lag=28	lag=29	lag=30
Markshalltown	9	80	47	49	79	9	83	8	77	47	46	76	8	83	9	75
Marlay Taylor	0	74	42	42	74	0	83	0	70	40	42	70	0	77	0	71
MDWASD Central	65	43	10	6	36	57	62	62	45	10	9	38	62	65	60	43
MDWASD North	10	83	52	54	78	11	85	11	78	51	51	79	10	86	11	76
MDWASD South	14	70	53	54	67	14	80	15	68	51	52	66	12	83	10	68
Merced	195	297	262	259	298	194	302	194	295	260	257	296	194	302	194	293
Mid-Coastside	29	153	102	96	153	25	161	28	153	93	96	146	28	162	29	153
Modesto's Sutter	259	251	239	237	250	261	284	258	249	236	237	248	261	280	256	247
Montpelier	38	38	38	38	36	36	66	36	36	36	38	36	34	65	34	32
Monterey One	48	84	54	57	72	52	86	47	70	55	58	70	54	80	60	65
Morris Forman	28	8	2	2	18	14	47	25	12	2	4	18	15	50	15	18
Mt. Pleasant	6	43	27	25	43	4	47	6	40	28	22	42	0	46	2	39
Muscatine	74	68	61	58	62	75	93	76	66	60	57	64	72	95	68	62
Norhtwest Water	20	171	117	114	169	20	179	19	169	114	120	162	20	179	18	163
North Water	3	111	66	63	112	3	117	3	111	63	61	111	3	116	3	107
Novato	8	169	103	104	168	7	170	7	167	104	98	169	8	171	8	165
Ocean	0	32	0	0	30	0	37	0	30	0	0	28	0	36	0	28
Oceanside	769	772	770	770	772	770	771	771	770	772	771	773	773	774	772	774
Ottumwa	26	87	60	62	86	23	97	20	90	59	55	86	24	90	26	82
Palo Alto	804	804	805	805	805	805	805	805	805	805	805	805	805	804	805	805
Parker North	183	117	10	12	115	180	184	179	113	9	12	117	179	181	177	110
Parker South	185	117	10	12	117	184	184	181	113	9	12	115	180	182	181	113
Paso Robles	158	162	160	160	160	158	249	156	160	158	158	158	156	249	154	158
Passaic Valley	111	78	58	56	85	107	114	110	79	57	51	82	112	108	102	85
Penacook	3	123	81	77	124	3	129	3	121	79	75	122	3	129	3	119
Portland	36	62	48	43	66	35	71	30	75	44	50	68	31	81	34	75
Provo City	17	78	4	4	74	18	87	18	76	4	4	72	18	85	17	78
Red Wing	12	26	18	13	24	13	29	12	21	19	13	26	10	30	11	20
Regional	26	85	62	64	82	28	89	24	85	60	62	81	26	86	22	80
Regional No. 1	135	118	107	106	116	134	181	131	116	107	104	114	131	179	130	112
River Road	92	62	2	2	60	89	99	90	62	0	2	58	86	96	85	58
Riverside	12	80	50	49	78	10	85	11	78	48	47	77	12	84	10	74
RM Clayton	14	100	67	66	100	14	109	13	100	63	66	96	12	109	11	99
Rochester	12	113	72	70	113	11	120	11	111	72	66	113	12	118	12	108
Sacramento	812	812	812	812	812	812	812	812	812	812	812	812	812	812	812	812
Salina	0	86	56	56	85	0	91	0	84	54	54	83	0	91	0	82
San Francisco	449	449	449	449	449	449	449	449	448	449	449	448	448	448	448	448

Table S13: Number of observations used in autocorrelation calculations by site and by lag for lags of 15 to 30 days.

Site Name	lag=15	lag=16	lag=17	lag=18	lag=19	lag=20	lag=21	lag=22	lag=23	lag=24	lag=25	lag=26	lag=27	lag=28	lag=29	lag=30
San Jose-Santa Clara	809	809	810	810	810	810	810	810	810	810	810	810	810	810	810	810
San Leandro	137	90	0	0	88	135	141	135	88	0	0	86	133	141	133	86
San Mateo & Estero	19	146	87	91	146	18	151	19	145	88	85	142	18	153	17	143
Santa Cruz (City)	24	193	127	123	189	26	194	27	190	124	123	188	24	198	24	188
Santa Cruz (County)	26	194	128	125	190	28	196	29	191	126	125	189	27	197	26	189
Santa Rosa	101	103	84	91	93	101	135	105	100	83	91	96	98	135	101	98
Sausalito-Marin	118	74	12	10	60	110	120	113	70	14	6	51	105	111	109	71
Seaford	0	77	48	46	77	0	81	0	75	46	44	75	0	81	0	73
Silicon Valley	807	806	807	808	808	808	808	808	808	808	808	808	808	808	807	808
SJRA No. 1	0	73	46	46	72	0	77	0	71	44	44	70	0	77	0	69
SJRA No. 2	0	73	46	46	72	0	77	0	71	44	44	70	0	77	0	69
SJRA No. 3	0	73	46	46	72	0	77	0	71	44	44	70	0	77	0	69
Somerset Raritan	2	33	20	18	32	0	36	2	30	18	18	29	2	34	2	29
Soscol	3	125	81	79	124	3	133	3	123	81	78	124	3	133	3	121
South Bay	26	17	13	15	21	23	30	24	15	11	14	21	21	27	22	11
South Bend	6	115	74	75	113	6	124	6	111	70	69	111	2	126	5	108
South Burlington	18	22	0	2	20	20	29	19	18	2	0	20	17	28	18	20
South Columbus	22	92	56	58	86	22	92	21	80	59	47	84	20	88	19	78
South County	809	809	809	810	810	810	810	810	810	810	810	810	810	810	810	810
South Laredo	2	88	45	46	86	3	90	3	85	45	48	83	3	90	3	83
South Monmouth	32	91	62	57	94	29	97	32	89	58	58	88	30	90	32	90
South River	11	100	66	65	100	11	109	10	100	62	65	96	10	109	8	99
South Water	16	172	118	114	171	15	180	16	169	114	118	164	14	180	12	164
Southern Marin	79	68	62	54	64	79	90	75	62	61	57	64	72	87	74	60
St. Cloud	15	51	28	32	47	14	55	15	46	26	33	44	14	51	16	44
Sunnyvale	804	804	804	805	805	805	805	805	805	805	805	805	805	805	805	805
Traverse City	56	52	52	50	50	55	84	54	50	50	48	48	53	83	52	48
Turkey Creek	3	110	73	71	107	3	120	3	104	67	67	105	3	122	3	102
Turlock	0	106	70	68	106	0	111	0	104	68	66	104	0	111	0	102
Upper Blackstone	7	61	36	36	60	7	65	7	58	35	35	57	7	64	7	55
Utoy Creek	11	100	66	65	100	11	109	10	100	62	65	96	10	109	8	99
Vallejo	137	92	4	4	90	135	141	135	90	4	4	88	133	141	133	88
Valley	0	150	98	96	150	0	154	0	148	96	94	148	0	154	0	146
Valley Creek	0	154	100	100	153	0	158	0	152	98	98	151	0	158	0	150
Village Creek	16	135	90	89	134	18	143	19	129	91	86	131	19	141	19	127
Warren	88	97	67	72	95	91	126	89	90	66	69	95	86	122	89	94
Weaton	0	137	84	82	136	0	140	0	135	82	80	134	0	140	0	133

Table S13: Number of observations used in autocorrelation calculations by site and by lag for lags of 15 to 30 days.

Site Name	lag=15	lag=16	lag=17	lag=18	lag=19	lag=20	lag=21	lag=22	lag=23	lag=24	lag=25	lag=26	lag=27	lag=28	lag=29	lag=30
West Boise	0	88	56	56	87	0	92	0	86	54	54	85	0	92	0	84
West County	90	73	67	60	78	84	131	93	71	57	60	72	87	124	86	69
Wheeling	0	48	28	24	48	0	52	0	45	28	22	48	0	52	0	43
Wichita Falls	20	92	62	59	88	22	97	21	86	64	59	87	16	98	19	90
Windsor	11	81	52	50	84	10	89	9	82	51	49	79	9	87	9	80
Winters	21	84	55	51	81	19	83	24	84	55	53	81	19	84	19	77
Wolcott	86	58	10	6	56	87	87	86	53	12	8	54	84	87	84	54
Woodland	0	98	68	68	100	0	107	0	98	66	66	97	0	107	0	96
Yankton	9	37	24	24	36	9	40	9	35	21	21	34	8	42	7	33
York	63	50	40	40	50	62	78	61	48	38	38	48	61	80	59	48
Youngstown	17	89	59	59	90	17	98	14	88	61	54	91	17	94	18	84
Zacate Creek	2	84	43	44	82	3	87	3	81	45	46	78	3	88	3	82

Table S14: Site summary statistics. Sites ordered alphabetically by abbreviation. PMMoV values are in $\log_{10}$ gene copies per gram dry weight. Precipitation values are in inches. (pop = population; obs = observations; prcp = precipitation)

Site	Location	Sewer	Pop Served	Obs	Min PMMoV	Max PMMoV	Med PMMoV	Mean PMMoV	Min Prcp	Max Prcp	Med Prcp	Mean Prcp
Akron	Akron, OH	Combined	365000	85.00	7.37	9.31	8.49	8.49	0.00	1.26	0.01	0.10
Altamonte Springs	Altamonte Springs, FL	Combined	95000	117.00	8.03	9.16	8.49	8.49	0.00	1.72	0.00	0.12
Ann Arbor	Ann Arbor, MI	Separated	125000	174.00	6.63	9.70	8.67	8.60	0.00	0.94	0.01	0.08
Aquia	Stafford, VA	Separated	100000	73.00	7.96	8.87	8.64	8.63	0.00	0.56	0.01	0.06
Archie Elledge	Winston-Salem, NC	Separated	92000	139.00	7.80	9.77	8.61	8.62	0.00	1.80	0.00	0.15
Bangor	Bangor, ME	Combined	40000	30.00	6.45	8.55	8.09	7.91	0.00	1.94	0.02	0.19
Bayshore	Union Beach, NJ	Separated	100000	48.00	7.97	9.22	8.47	8.48	0.00	2.22	0.00	0.17
Big Creek	Roswell, GA	Separated	189593	173.00	7.69	9.84	8.63	8.63	0.00	3.25	0.01	0.15
Boege Alvarado (Fremont)	Union City, CA	Separated	229476	75.00	8.33	9.81	8.71	8.76	0.00	1.06	0.00	0.09
Boege Alvarado (Newark)	Union City, CA	Separated	47229	76.00	8.09	10.14	8.74	8.81	0.00	1.96	0.00	0.11
Boege Alvarado (Union City)	Union City, CA	Separated	68150	74.00	8.36	10.03	8.72	8.74	0.00	1.96	0.00	0.12
Brunswick	Brunswick, ME	Separated	10000	100.00	7.60	8.86	8.17	8.18	0.00	2.07	0.01	0.15
CODIGA	CODIGA, CA	Separated	10000	600.00	7.29	11.10	8.73	8.83	0.00	3.00	0.00	0.06
Cahaba River	Birmingham, AL	Separated	95000	132.00	8.06	9.06	8.58	8.57	0.00	3.26	0.02	0.16
Calera Creek	Pacifica, CA	Separated	40000	125.00	8.00	9.74	8.66	8.66	0.00	1.67	0.00	0.12
Camp Creek	College Park, GA	Separated	73821	175.00	7.76	9.83	8.46	8.45	0.00	3.25	0.01	0.14
Capital Region	Harrisburg, PA	Combined	125000	163.00	7.84	9.60	8.49	8.49	0.00	2.42	0.01	0.11
Carmel	Carmel, IN	Separated	86000	46.00	8.43	9.68	8.80	8.83	0.00	0.71	0.00	0.08
Central Contra Costa	Martinez, CA	Separated	484800	200.00	8.47	9.60	8.95	8.95	0.00	2.25	0.00	0.06
Central Marin	San Rafael, CA	Separated	104250	95.00	7.83	9.65	8.63	8.64	0.00	1.71	0.00	0.12
Central Marin (W Railroad)	San Rafael, CA	Separated	25000	97.00	8.42	10.24	8.93	8.96	0.00	2.55	0.00	0.14
Central Valley	Salt Lake City, UT	Separated	600000	125.00	8.47	10.34	8.98	8.99	0.00	1.11	0.04	0.13
Clark County	Las Vegas, NV	Separated	990000	62.00	8.03	9.26	8.63	8.65	0.00	0.38	0.00	0.02
Clinton	Clinton, IA	Combined	29300	91.00	7.55	8.34	8.14	8.10	0.00	1.29	0.00	0.10
Coastal	Laguna Niguel, CA	Separated	48000	103.00	7.94	9.48	8.78	8.79	0.00	2.16	0.00	0.10
Coeur d'Alene	Coeur D Alene, ID	Separated	50540	226.00	7.52	9.62	8.65	8.65	0.00	1.19	0.01	0.09
Coralville	Coralville, IA	Separated	23000	86.00	7.94	9.52	8.62	8.59	0.00	1.57	0.00	0.10
Cumberland	Bridgeton, NJ	Separated	50000	64.00	8.25	9.27	8.77	8.74	0.00	1.14	0.00	0.10
DELCORA	Chester, PA	Combined	220000	110.00	7.54	8.99	8.31	8.31	0.00	1.90	0.00	0.13
Davis	Davis, CA	Combined	68000	678.00	8.23	10.34	8.93	8.94	0.00	3.87	0.00	0.06
Deer Island	Boston Metropolitan Area, MA	Combined	2400000	101.00	7.87	9.05	8.42	8.42	0.00	1.65	0.02	0.18
Dillman Road	Bloomington, IN	Separated	56090	147.00	7.82	9.46	8.51	8.49	0.00	1.90	0.00	0.11
Dover	Dover, NH	Separated	30000	104.00	7.75	9.78	8.32	8.31	0.00	1.71	0.02	0.17
Duck Creek	Sunnyvale, TX	Separated	186000	171.00	8.36	9.29	8.83	8.84	0.00	2.02	0.00	0.08
E.W. Blom Point Loma	San Diego, CA	Separated	2200000	154.00	8.11	10.16	9.11	9.08	0.00	1.93	0.00	0.10

Table S14: Site summary statistics. Sites ordered alphabetically by abbreviation. PMMoV values are in $\log_{10}$ gene copies per gram dry weight. Precipitation values are in inches. (pop = population; obs = observations; prcp = precipitation)

Site	Location	Sewer	Pop Served	Obs	Min PMMoV	Max PMMoV	Med PMMoV	Mean PMMoV	Min Prcp	Max Prcp	Med Prcp	Mean Prcp
East Bay	Oakland, CA	Separated	740000	238.00	7.41	9.56	8.77	8.75	0.00	1.16	0.00	0.06
Eastern	Orlando, FL	Separated	195299	195.00	7.66	9.70	8.21	8.26	0.00	3.36	0.02	0.16
Ellis Creek	Petaluma, CA	Separated	65000	141.00	8.26	9.43	8.76	8.77	0.00	2.01	0.00	0.15
Esparto	Esparto, CA	Separated	4006	103.00	7.95	10.57	9.02	9.09	0.00	1.52	0.00	0.11
Essex	Essex Junction, VT	Separated	30000	65.00	7.90	9.48	8.50	8.49	0.00	1.35	0.02	0.15
Fairfield-Suisun	Fairfield, CA	Separated	155000	137.00	8.50	9.85	8.95	8.95	0.00	2.17	0.00	0.12
Five Mile Creek	Fultondale, AL	Separated	77000	123.00	6.91	8.84	8.45	8.42	0.00	3.26	0.01	0.17
Gainesville	Gainesville, TX	Separated	17300	91.00	8.11	9.42	8.79	8.79	0.00	1.91	0.00	0.13
Garland Rowlett Creek	Garland, TX	Separated	200000	223.00	8.29	11.17	8.99	9.00	0.00	4.34	0.00	0.11
Glenbard	Glen Ellyn, IL	Combined	86000	150.00	7.90	9.42	8.63	8.66	0.00	2.48	0.00	0.12
Grandville	Jenison, MI	Separated	75000	108.00	8.07	9.39	8.65	8.64	0.00	0.84	0.01	0.10
Hagerstown	Hagerstown, MD	Separated	90000	100.00	7.18	9.60	8.45	8.45	0.00	1.21	0.00	0.09
Hall Street	Concord, NH	Separated	45000	132.00	7.88	9.28	8.27	8.29	0.00	1.78	0.01	0.18
Hamlin	Winter Garden, FL	Separated	50000	106.00	7.69	9.77	8.12	8.18	0.00	3.36	0.01	0.16
Harrison	Harrison, AR	Combined	15000	48.00	7.37	8.71	8.23	8.21	0.00	1.02	0.00	0.13
Hillsville	Hillsville, VA	Separated	3000	105.00	7.73	8.51	8.26	8.25	0.00	1.95	0.00	0.11
Hollister	Hollister, CA	Combined	42000	133.00	8.66	10.54	9.21	9.21	0.00	1.76	0.00	0.09
Hollywood Road	Amarillo, TX	Separated	60000	106.00	5.92	9.91	8.63	8.57	0.00	0.92	0.00	0.04
Hyperion	Playa Del Rey, CA	Separated	4000000	151.00	8.37	9.86	9.00	9.02	0.00	2.54	0.00	0.10
JB Latham	Laguna Niguel, CA	Separated	120000	103.00	8.22	10.12	8.78	8.80	0.00	2.16	0.00	0.10
Jackson	Jackson, MI	Separated	90000	206.00	8.06	9.09	8.56	8.56	0.00	1.81	0.01	0.12
Jeffersonville	Jeffersonville, IN	Combined	25000	121.00	7.63	9.04	8.31	8.32	0.00	1.81	0.00	0.16
John M. Asplund	Anchorage, AK	Separated	220000	34.00	8.03	9.40	8.48	8.48	0.00	0.88	0.04	0.12
Johnnie Mosley	Kinston, NC	Separated	25000	103.00	7.27	8.67	8.03	8.04	0.00	1.47	0.00	0.10
Johns Creek	Roswell, GA	Separated	84486	172.00	7.68	9.39	8.61	8.62	0.00	3.25	0.01	0.15
Joint	Carson, CA	Separated	3500000	248.00	7.98	9.78	9.13	9.14	0.00	2.00	0.00	0.06
Kansas City	Kansas City, KS	Separated	35000	95.00	8.14	9.58	8.74	8.76	0.00	1.57	0.00	0.13
Kaw Point	Kansas City, KS	Combined	90000	94.00	7.60	9.18	8.79	8.77	0.00	1.57	0.00	0.13
Lancaster	Lancaster, CA	Separated	200000	145.00	8.29	10.25	8.94	8.96	0.00	1.35	0.00	0.06
Lander Street	Boise, ID	Separated	108556	92.00	7.83	8.99	8.47	8.48	0.00	0.32	0.00	0.03
Las Gallinas	San Rafael, CA	Separated	30000	163.00	7.89	9.83	8.70	8.71	0.00	2.95	0.00	0.16
Lawrence Kansas	Lawrence, KS	Separated	80000	150.00	7.60	9.40	8.65	8.64	0.00	3.48	0.00	0.09
Little Falls Run	Fredericksburg, VA	Separated	50000	72.00	8.17	8.89	8.64	8.62	0.00	0.56	0.01	0.06
Little River	Roswell, GA	Separated	12818	172.00	7.42	9.41	8.32	8.33	0.00	3.25	0.01	0.15
Lompoc	Lompoc, CA	Separated	69290	162.00	8.38	9.93	9.10	9.10	0.00	2.87	0.00	0.08

Table S14: Site summary statistics. Sites ordered alphabetically by abbreviation. PMMoV values are in $\log_{10}$ gene copies per gram dry weight. Precipitation values are in inches. (pop = population; obs = observations; prcp = precipitation)

Site	Location	Sewer	Pop Served	Obs	Min PMMoV	Max PMMoV	Med PMMoV	Mean PMMoV	Min Prcp	Max Prcp	Med Prcp	Mean Prcp
Los Banos	Los Banos, CA	Separated	42000	113.00	8.50	10.12	9.04	9.09	0.00	0.78	0.00	0.06
Loxahatchee	Jupiter, FL	Separated	90000	142.00	7.56	9.87	8.30	8.36	0.00	3.81	0.03	0.21
MDWASD Central	Key Biscayne, FL	Separated	829725	79.00	7.66	8.87	8.18	8.19	0.00	2.84	0.10	0.25
MDWASD North	North Miami, FL	Separated	776150	91.00	6.57	8.75	8.10	8.03	0.00	1.54	0.05	0.22
MDWASD South	Miami, FL	Separated	920528	88.00	7.88	9.00	8.28	8.29	0.00	2.84	0.05	0.25
Madera	Madera, CA	Separated	67944	68.00	8.74	9.74	9.11	9.11	0.00	2.08	0.00	0.09
Mankato	Mankato, MN	Separated	70000	149.00	8.08	9.77	8.63	8.62	0.00	1.93	0.00	0.10
Markshalltown	Marshalltown, IA	Separated	27400	87.00	8.00	9.37	8.79	8.82	0.00	1.02	0.00	0.06
Marlay Taylor	Hollywood, MD	Separated	55000	83.00	7.79	9.28	8.36	8.36	0.00	1.20	0.00	0.08
Merced	Merced, CA	Separated	91000	303.00	8.48	10.23	9.24	9.25	0.00	1.45	0.00	0.05
Mid-Coastside	Half Moon Bay, CA	Separated	28000	182.00	8.08	10.86	8.79	8.83	0.00	1.87	0.00	0.07
Modesto's Sutter	Modesto, CA	Separated	230000	294.00	8.61	9.72	9.18	9.16	0.00	2.23	0.00	0.06
Montpelier	Montpelier, VT	Combined	10100	67.00	7.54	9.37	8.52	8.48	0.00	2.66	0.02	0.20
Monterey One	Marina, CA	Separated	262000	107.00	8.50	10.13	9.12	9.13	0.00	1.64	0.00	0.12
Morris Forman	Louisville, KY	Combined	423913	75.00	7.67	8.66	8.20	8.18	0.00	2.60	0.00	0.13
Mt. Pleasant	Mt. Pleasant, MI	Separated	21690	52.00	7.76	8.96	8.39	8.41	0.00	1.43	0.00	0.09
Muscatine	Muscatine, IA	Combined	24400	107.00	7.59	9.55	8.61	8.64	0.00	1.68	0.00	0.11
Norhtwest Water	Orlando, FL	Separated	66690	196.00	7.36	9.95	8.25	8.29	0.00	3.36	0.02	0.17
North Water	Jeffersonville, IN	Separated	25000	119.00	7.93	8.83	8.42	8.41	0.00	1.81	0.00	0.16
Novato	Novato, CA	Separated	53000	176.00	7.85	9.61	8.69	8.70	0.00	2.96	0.00	0.13
Ocean	Oakhurst, NJ	Separated	50000	37.00	8.15	9.26	8.71	8.70	0.00	0.41	0.00	0.05
Oceanside	San Francisco, CA	Combined	250000	776.00	8.02	10.83	8.75	8.75	0.00	3.15	0.00	0.08
Ottumwa	Ottumwa, IA	Combined	25529	104.00	7.84	9.00	8.36	8.35	0.00	0.71	0.00	0.07
Palo Alto	Palo Alto, CA	Separated	236000	805.00	8.35	11.31	9.01	9.04	0.00	3.00	0.00	0.06
Parker North	Parker, CO	Separated	35000	191.00	8.14	10.69	8.60	8.63	0.00	1.42	0.00	0.06
Parker South	Parker, CO	Separated	25000	192.00	7.86	9.42	8.54	8.55	0.00	1.42	0.00	0.06
Paso Robles	Paso Robles, CA	Separated	31037	249.00	8.56	9.89	9.21	9.20	0.00	2.83	0.00	0.07
Passaic Valley	Newark, NJ	Separated	1500000	146.00	6.79	9.74	8.44	8.44	0.00	2.15	0.02	0.16
Penacook	Concord, NH	Separated	4000	130.00	7.79	9.43	8.28	8.30	0.00	1.78	0.01	0.18
Portland	Portland, ME	Combined	65000	111.00	7.81	8.94	8.42	8.40	0.00	1.88	0.01	0.13
Provo City	Provo, UT	Separated	115000	95.00	8.51	9.52	8.90	8.91	0.00	0.82	0.02	0.10
RM Clayton	Atlanta, GA	Separated	294660	116.00	8.09	9.28	8.60	8.60	0.00	2.01	0.02	0.15
Red Wing	Red Wing, MN	Separated	16000	36.00	8.45	8.91	8.63	8.66	0.00	1.30	0.00	0.06
Regional	Laguna Niguel, CA	Separated	129000	102.00	8.10	10.00	8.72	8.73	0.00	2.16	0.00	0.10
Regional No. 1	Ontario, CA	Separated	890000	193.00	8.40	10.02	9.03	9.04	0.00	0.90	0.00	0.04

Table S14: Site summary statistics. Sites ordered alphabetically by abbreviation. PMMoV values are in $\log_{10}$ gene copies per gram dry weight. Precipitation values are in inches. (pop = population; obs = observations; prcp = precipitation)

Site	Location	Sewer	Pop Served	Obs	Min PMMoV	Max PMMoV	Med PMMoV	Mean PMMoV	Min Prcp	Max Prcp	Med Prcp	Mean Prcp
River Road	Amarillo, TX	Separated	140000	103.00	8.13	9.47	8.75	8.74	0.00	0.92	0.00	0.04
Riverside	Riverside, CA	Separated	350000	88.00	8.47	9.90	9.07	9.09	0.00	1.05	0.00	0.04
Rochester	Rochester, MN	Separated	120000	122.00	8.22	9.92	8.68	8.70	0.00	0.92	0.00	0.09
SJRA No. 1	Woodlands, TX	Separated	65000	77.00	7.79	9.82	8.35	8.35	0.00	1.94	0.00	0.11
SJRA No. 2	Woodlands, TX	Separated	70000	77.00	7.90	9.21	8.34	8.38	0.00	1.94	0.00	0.11
SJRA No. 3	Woodlands, TX	Separated	15000	77.00	7.91	9.04	8.47	8.44	0.00	1.94	0.00	0.11
Sacramento	Sacramento, CA	Combined	1480000	812.00	8.33	10.14	8.98	8.99	0.00	4.26	0.00	0.06
Salina	Salina, KS	Separated	47000	91.00	8.54	9.45	9.07	9.07	0.00	1.44	0.00	0.08
San Francisco	San Francisco, CA	Combined	750000	449.00	8.13	9.78	8.89	8.89	0.00	3.14	0.00	0.08
San Jose-Santa Clara	San Jose, CA	Separated	1500000	810.00	8.60	10.57	9.17	9.18	0.00	3.00	0.00	0.06
San Leandro	San Leandro, CA	Separated	50000	141.00	7.66	9.64	8.82	8.78	0.00	1.96	0.00	0.10
San Mateo & Estero	San Mateo, CA	Separated	150000	162.00	8.11	9.86	8.88	8.87	0.00	1.67	0.00	0.10
Santa Cruz (City)	Santa Cruz, CA	Separated	160000	209.00	7.90	11.04	8.70	8.75	0.00	2.93	0.00	0.13
Santa Cruz (County)	Santa Cruz, CA	Separated	160000	210.00	8.23	10.64	8.69	8.73	0.00	2.93	0.00	0.13
Santa Rosa	Santa Rosa, CA	Separated	230000	155.00	8.29	9.88	9.16	9.16	0.00	2.27	0.00	0.13
Sausalito-Marin	Sausalito, CA	Separated	18000	134.00	7.29	9.29	8.54	8.54	0.00	1.71	0.00	0.11
Seaford	Seaford, DE	Separated	13172	81.00	7.95	9.49	8.59	8.60	0.00	1.69	0.00	0.10
Silicon Valley	Silicon Valley, CA	Separated	199000	808.00	7.98	10.37	8.98	8.99	0.00	3.71	0.00	0.08
Somerset Raritan	Bridgewater, NJ	Separated	130000	39.00	7.88	8.97	8.41	8.39	0.00	1.95	0.00	0.20
Soscol	Napa, CA	Combined	83300	134.00	8.85	9.91	9.27	9.29	0.00	2.20	0.00	0.12
South Bay	San Diego, CA	Separated	1600000	36.00	8.91	9.93	9.65	9.62	0.00	0.68	0.00	0.11
South Bend	South Bend, IN	Combined	130000	130.00	8.08	9.36	8.63	8.63	0.00	1.28	0.01	0.11
South Burlington	South Burlington, VT	Separated	16000	39.00	8.21	8.73	8.47	8.48	0.00	1.83	0.02	0.20
South Columbus	Columbus, GA	Combined	278000	118.00	7.69	9.16	8.49	8.49	0.00	2.32	0.00	0.14
South County	Gilroy, CA	Separated	110338	810.00	8.38	10.69	8.98	9.01	0.00	3.00	0.00	0.06
South Laredo	Laredo, TX	Separated	120000	94.00	8.63	10.05	9.39	9.38	0.00	1.58	0.00	0.03
South Monmouth	Belmar, NJ	Separated	52672	109.00	7.73	10.06	8.46	8.49	0.00	2.22	0.00	0.13
South River	Atlanta, GA	Separated	105160	115.00	7.71	9.04	8.40	8.37	0.00	2.01	0.02	0.16
South Water	Orlando, FL	Separated	183009	195.00	7.91	9.18	8.39	8.40	0.00	3.36	0.03	0.17
Southern Marin	Mill Valley, CA	Separated	30000	107.00	7.73	8.86	8.47	8.47	0.00	2.95	0.00	0.19
St. Cloud	St. Cloud, MN	Separated	120000	59.00	8.10	9.44	8.52	8.50	0.00	1.95	0.01	0.10
Sunnyvale	Sunnyvale, CA	Separated	153000	805.00	8.46	10.73	9.29	9.30	0.00	3.00	0.00	0.06
Traverse City	Traverse City, MI	Separated	30623	88.00	8.01	9.02	8.43	8.43	0.00	1.33	0.00	0.08
Turkey Creek	Pinson, AL	Separated	30000	125.00	7.71	10.14	8.40	8.42	0.00	3.26	0.01	0.16
Turlock	Turlock, CA	Separated	86000	111.00	7.98	10.16	8.67	8.70	0.00	1.51	0.00	0.09

Table S14: Site summary statistics. Sites ordered alphabetically by abbreviation. PMMoV values are in $\log_{10}$ gene copies per gram dry weight. Precipitation values are in inches. (pop = population; obs = observations; prcp = precipitation)

Site	Location	Sewer	Pop Served	Obs	Min PMMoV	Max PMMoV	Med PMMoV	Mean PMMoV	Min Prcp	Max Prcp	Med Prcp	Mean Prcp
Upper Blackstone	Millbury, MA	Separated	250000	69.00	7.66	8.77	8.21	8.24	0.00	1.60	0.02	0.19
Utoy Creek	Atlanta, GA	Separated	70887	115.00	7.78	9.30	8.30	8.32	0.00	2.01	0.02	0.16
Vallejo	Vallejo, CA	Separated	121000	143.00	8.20	10.70	8.95	8.95	0.00	1.62	0.00	0.11
Valley	Indio, CA	Separated	91765	154.00	8.61	10.93	9.31	9.34	0.00	1.05	0.00	0.05
Valley Creek	Bessemer, AL	Separated	225000	158.00	7.75	9.07	8.51	8.52	0.00	3.26	0.02	0.18
Village Creek	Birmingham, AL	Separated	200000	150.00	7.85	10.11	8.47	8.47	0.00	1.93	0.01	0.12
Warren	Warren, MI	Separated	140000	137.00	7.87	9.26	8.43	8.44	0.00	1.11	0.01	0.11
Weaton	Wheaton, IL	Separated	63000	141.00	8.05	9.85	8.76	8.75	0.00	1.40	0.00	0.09
West Boise	Boise, ID	Separated	186901	92.00	8.06	9.56	8.49	8.48	0.00	0.32	0.00	0.03
West County	Richmond, CA	Separated	100000	151.00	7.80	10.36	8.83	8.84	0.00	1.60	0.00	0.07
Wheeling	Wheeling, WV	Combined	100000	52.00	8.02	8.68	8.38	8.38	0.00	1.33	0.01	0.13
Wichita Falls	Wichita Falls, TX	Separated	90000	107.00	8.32	9.22	8.89	8.89	0.00	0.90	0.00	0.07
Windsor	Windsor, CA	Separated	28000	95.00	8.20	10.60	8.90	8.89	0.00	3.31	0.00	0.21
Winters	Winters, CA	Separated	7286	101.00	8.22	10.70	8.79	8.83	0.00	1.61	0.00	0.12
Wolcott	Kansas City, KS	Separated	15000	93.00	7.26	9.25	8.60	8.60	0.00	1.57	0.00	0.12
Woodland	Woodland, CA	Separated	59000	108.00	8.54	9.98	9.00	9.07	0.00	1.52	0.00	0.11
Yankton	Yankton, SD	Separated	20000	47.00	7.96	9.59	8.40	8.42	0.00	2.13	0.00	0.11
York	York Beach, ME	Separated	10000	88.00	7.51	9.13	8.04	8.07	0.00	2.74	0.01	0.23
Youngstown	Youngstown, OH	Combined	174000	103.00	7.26	8.97	8.37	8.33	0.00	1.02	0.00	0.09
Zacate Creek	Laredo, TX	Separated	140000	92.00	8.76	10.46	9.31	9.35	0.00	1.58	0.00	0.03

References

[S1] Dragulescu, A.; Arendt, C. xlsx: Read, Write, Format Excel 2007 and Excel 97/2000/XP/2003 Files. 2020; R package version 0.6.5.

[S2] Wickham, H.; François, R.; Henry, L.; Müller, K.; Vaughan, D. dplyr: A Grammar of Data Manipulation. 2023; R package version 1.1.4.

[S3] Bache, S. M.; Wickham, H. magrittr: A Forward-Pipe Operator for R. 2022; R package version 2.0.3.

[S4] Rozzi, G. C. zipcodeR: Advancing the analysis of spatial data at the ZIP code level in R. *Software Impacts* **2021**, *9*, 100099.

[S5] Ramsay, J. fda: Functional Data Analysis. 2023; R package version 6.1.4.

[S6] R Core Team R: A Language and Environment for Statistical Computing. R Foundation for Statistical Computing: Vienna, Austria, 2021.

[S7] Koenker, R. quantreg: Quantile Regression. 2022; R package version 5.94.

[S8] Koenker, R. W.; D'Orey, V. Computing Regression Quantiles. *Journal of the Royal Statistical Society Series C* **1987**, *36*, 383–393.

[S9] Koenker, R.; d'Orey, V. A Remark on Algorithm as 229: Computing Dual Regression Quantiles and Regression Rank Scores. *Journal of the Royal Statistical Society Series C* **1994**, *43*, 410–414.

[S10] Hagemann, A. Cluster-Robust Bootstrap Inference in Quantile Regression Models. *Journal of the American Statistical Association* **2017**, *112*, 446–456.

[S11] Geraci, M. Linear Quantile Mixed Models: The lqmm Package for Laplace Quantile Regression. *Journal of Statistical Software* **2014**, *57*, 1–29.

[S12] Geraci, M.; Bottai, M. Linear quantile mixed models. *Statistics and Computing* **2014**, *24*, 461–479.

[S13] Hijmans, R. J. geosphere: Spherical Trigonometry. 2022; R package version 1.5-18.

[S14] Stan Development Team RStan: the R interface to Stan. 2024; https://mc-stan.org/, R package version 2.32.6.

[S15] Tarroso, P.; Carvalho, S. B.; Velo-Anton, G. phylin 2.0: Extending the phylogeographic interpolation method to include uncertainty and user-defined distance metrics. *Molecular Ecology Resources* **2019**, *19*, 1081–1094.

[S16] Tarroso, P.; Velo-Anton, G.; Carvalho, S. B. phylin: an R package for phylogeographic interpolation. *Molecular Ecology Resources* **2015**, *15*, 349–357.

[S17] Wickham, H. *ggplot2: Elegant Graphics for Data Analysis*; Springer-Verlag New York, 2016.

[S18] Slowikowski, K. ggrepel: Automatically Position Non-Overlapping Text Labels with 'ggplot2'. 2021; R package version 0.9.1.

[S19] Wickham, H.; Pedersen, T. L.; Seidel, D. scales: Scale Functions for Visualization. 2023; R package version 1.3.0.

[S20] Kassambara, A. ggpubr: 'ggplot2' Based Publication Ready Plots. 2020; R package version 0.4.0.

[S21] Garnier; Simon; Ross; Noam; Rudis; Robert; Camargo; Pedro, A.; Sciaini; Marco; Scherer; Cédric viridis - Colorblind-Friendly Color Maps for R. 2021; R package version 0.6.2.

[S22] Wilke, C. O. cowplot: Streamlined Plot Theme and Plot Annotations for 'ggplot2'. 2020; R package version 1.1.1.

[S23] Pebesma, E. Simple Features for R: Standardized Support for Spatial Vector Data. *The R Journal* **2018**, *10*, 439–446.

[S24] Pebesma, E.; Bivand, R. *Spatial Data Science: With applications in R*; Chapman and Hall/CRC, 2023; p 352.

[S25] Gombin, J.; Vaidyanathan, R.; Agafonkin, V. concaveman: A Very Fast 2D Concave Hull Algorithm. 2020; R package version 1.1.0.

[S26] Google Maps. 2024; https://www.google.com/maps/place/Verily+Life+Sciences/@37.647493,-122.4326917,11155m/data=!3m1!1e3!4m10!1m2!2m1!1sVerily+Life+Sciences!3m6!1s0x808f784de278121f:0xaea30a44ff39aa66!8m2!3d37.6538123!4d-122.3950354!15sChRWZXJpbHkgTGlmZSBTY2llbmNlc1oWIhR2ZXJpbHkgbGlmZSBzY2llbmNlc5IBFWJpb3RlY2hub2xv16s%2Fg%2F11cn5qqmsn?entry=ttu&g_ep=EgoyMDI0MDkwMi4wIKXMDSoASAFQAw%3D%3D, Map of Verily Life Sciences.

[S27] Google Maps. 2024; https://www.google.com/maps/place/El+Paso,+TX/@31.8110291,-106.5893211,85126m/data=!3m2!1e3!4b1!4m6!3m5!1s0x86e73f8bc5fe3b69:0xe39184e3ab9d0222!8m2!3d31.7618778!4d-106.4850217!16zL20vMDEwMG10?entry=ttu&g_ep=EgoyMDI0MDkwMi4wIKXMDSoASAFQAw%3D%3D, Map of El Paso, Texas.

[S28] Brockwell, P.; Davis, R. *Introduction to Time Series and Forecasting*; Springer Texts in Statistics; Springer New York, 2006.
